# Bis(methylene)-λ^5^-phosphane anions†

**DOI:** 10.1039/d4sc07246d

**Published:** 2024-11-14

**Authors:** Akihiro Nomoto, Koh Sugamata, Takahiro Sasamori

**Affiliations:** a Graduate School of Science and Technology, University of Tsukuba 1-1-1 Tennoudai Tsukuba Ibaraki 305-8571 Japan sasamori@chem.tsukuba.ac.jp; b Department of Chemistry, College of Science, Rikkyo University 3-34-1 Nishi-Ikebukuro, Toshima-ku Tokyo 171-8501 Japan sugamata@rikkyo.ac.jp; c Division of Chemistry, Institute of Pure and Applied Sciences, University of Tsukuba 1-1-1 Tennoudai Tsukuba Ibaraki 305-8571 Japan; d Tsukuba Research Center for Energy Materials Sciences (TREMS), University of Tsukuba 1-1-1 Tennoudai Tsukuba Ibaraki 305-8571 Japan

## Abstract

Bis(methylene)-λ^5^-phosphane anions, *i.e.*, anionic phosphorus-centered heteroallene-type molecules, were obtained from the desilylation of a bis(silyl)methyl-substituted phosphaalkene. Their molecular structures, which were determined using spectroscopic techniques and single-crystal X-ray diffraction analysis, suggest that the central di-coordinated P atom is engaged in cumulative C=P=C π-bonds with the neighboring C atoms. The π-bond character of the C=P=C moieties was examined on the basis of the experimental results in combination with theoretical calculations; the results obtained suggest that multiple silyl substitutions at the C atom weaken the C=P π-bonding character.

## Abstract 2

Novel anionic phosphorus-centered heteroallene type molecules, bis(methylene)-λ^5^-phosphane anions, were synthesized and fully characterized.

## Graphical abstract

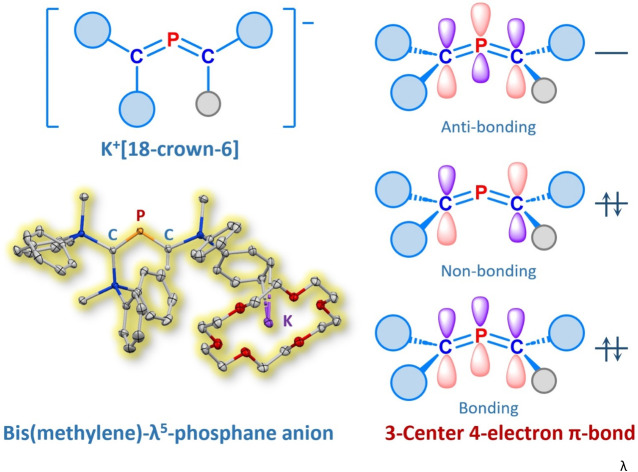

## Introduction

**Fig. 1:**
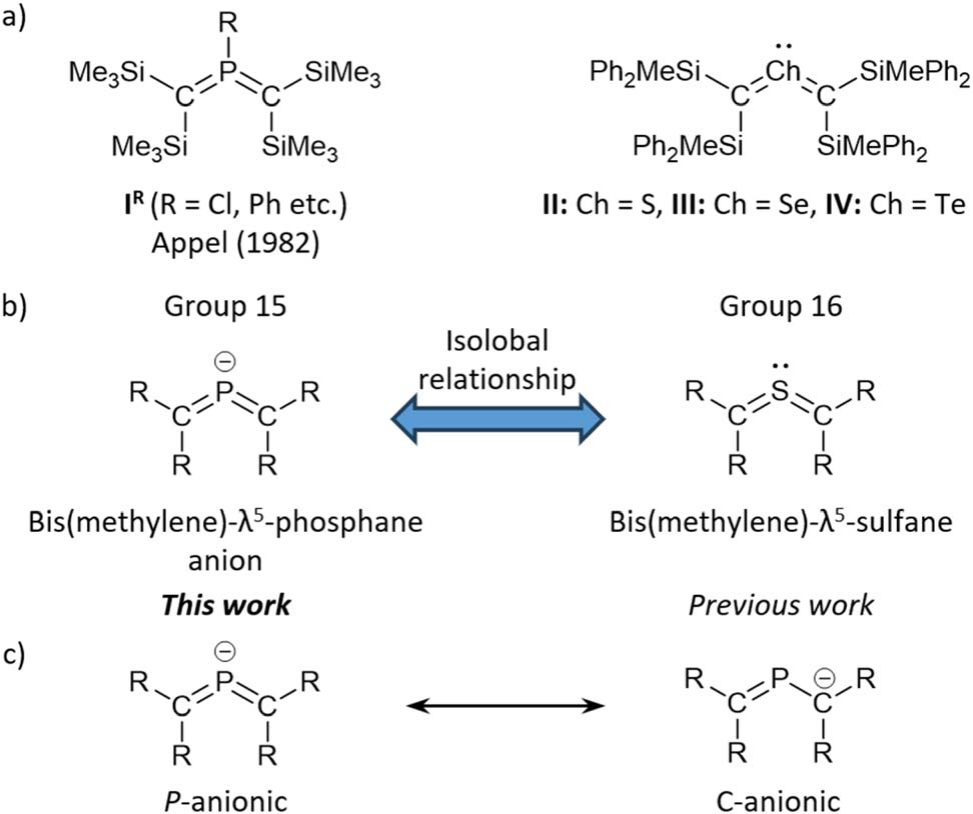
(a) Isolated bis(methylene)-λ^5^-phosphanes I^R^ and bis(methylene)-λ^4^-chalcogenanes II–IV. (b) Isolobal relationship between bis(methylene)-λ^5^-phosphane anion and bis(methylene)-λ^4^-sulfane. (c) Canonical resonance structures of bis(methylene)-λ^5^-phosphane anions.

Bis(methylene)-λ^5^-phosphanes, *i.e.*, phosphorus-centered heteroallene-type molecules, have attracted much attention as valence isomers of *σ*^3^,λ^5^-phosphiranes owing to their expected unique bonding character due to the cumulative C=P π-bonds. In 1982,^1^ Appel reported the first example of isolable bis(methylene)-λ^5^-phosphanes (I^R^, R = C_6_H_11_, Ph, and Me_2_N), whose structural characterization revealed a cumulative C=P=C π-bond with a tri-coordinated P(v) atom. This *σ*^3^,λ^5^-phosphane, *i.e.*, a phosphorus(v)-centered allene, represented a milestone in main-group-element chemistry as an unprecedented low-coordinated organophosphorus compound alongside West's disilene and Yoshifuji's diphosphene.^2,3^ The hitherto isolated bis(methylene)-λ^5^-phosphanes exhibit a distinct bent-allenic structure that is significantly different from the linear structure of all-carbon allenes.^4^ Bis(methylene)-λ^5^-phosphanes have so far been obtained by treating the corresponding carbenes or carbenoids with the corresponding phosphaalkenes.^5^ Furthermore, the *P*-chloro-substituted analogues of bis(methylene)-λ^5^-phosphanes (I^Cl^, Fig. 1) can be expected to serve as suitable precursors to a variety of functionalized bis(methylene)-λ^5^-phosphanes, given that I^Cl^ can be easily functionalized at the phosphorus atom *via* nucleophilic substitution reactions (Fig. 1a).^6–11^ The diverse reactivity of bis(methylene)-λ^5^-phosphanes prompted us to focus our attention on the synthesis of a *P*-anionic bis(methylene)-λ^5^-phosphane, which is expected to work as a nucleophilic building block for hitherto unknown types of bis(methylene)-λ^5^-phosphanes.

Recently, we have successfully synthesized stable bis(methylene)-λ^4^-sulfane (II, Fig. 1), which represents the first example of a group-16-element-centered heteroallene, using steric stabilization afforded by silyl groups.^12^ Moreover, its heavier-element analogues, *i.e.*, bis(methylene)-λ^4^-selane III and -tellane IV, have been synthesized and fully characterized.^13,14^ These bis(methylene)-λ^4^-chalcogenanes, which exhibit pseudo-*C*_2v_ symmetric coordination geometries with bent allene-type electronic structures, can be interpreted as “2-heteroallenes”,^15^ characterized by the three-center-four-electron π-bond on the C=Ch=C (Ch = S, Se, Te) moiety. Given that bis(methylene)-λ^4^-chalcogenanes are isolobal to anionic group-15-element-centered 2-heteroallenes (Fig. 1b), we expected that anionic bis(methylene)-λ^5^-phosphanes could potentially be synthesized using sterically demanding silyl groups as in the cases of II–IV, which may prevent the isomerization to the corresponding phosphiranides.^16,17^ As described above, bis(methylene)-λ^5^-phosphane anions should be of great interest not only with respect to their expected unique electronic properties, but also with regard to their potential as precursors to further functionalized bis(methylene)-λ^5^-phosphanes upon treatment with electrophiles or electron-deficient metals. Moreover, a detailed examination of the intrinsic nature of these species, *i.e.*, whether they should be considered *P*-anionic bis(methylene)-λ^5^-phosphanes or C-anionic phosphaalkenes, would be of great importance and interest (Fig. 1c). Here, we present the successful isolation of bis(methylene)-λ^5^-phosphane anions and their structural characterization.

## Results and discussion

**Scheme 1:**
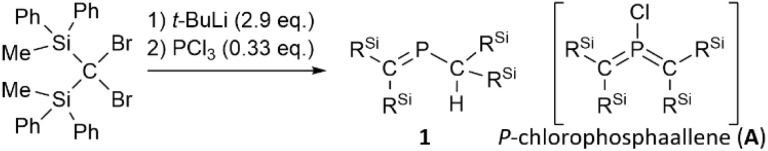
The synthesis of phosphaalkene 1.

**Fig. 2:**
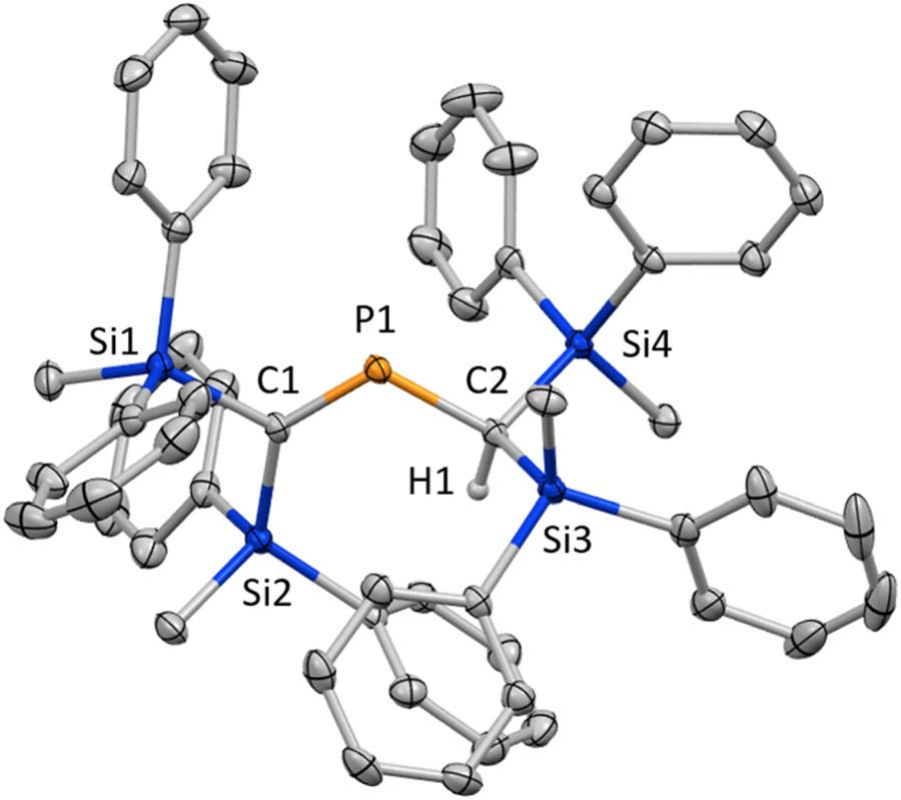
Molecular structure of 1 in the crystalline state with thermal ellipsoids at 50% probability; all hydrogen atoms except for H1 are omitted for clarity. Selected bond lengths [Å] and angles [°]: C1–P1 1.675(2), P1–C2 1.835(2), and C1–P1–C2 112.63(9).^19^

**Scheme 2:**
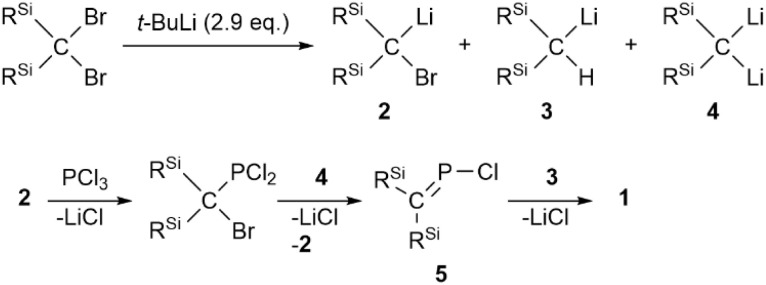
Plausible reaction mechanism for the formation of phosphaalkene 1.

To start with, we attempted the synthesis of *P*-chlorophosphaallene A as a precursor for the bis(methylene)-λ^5^-phosphane anions according to a previously reported protocol.^18^ The reaction of bis(silyl)carbenoid R^Si^_2_CBrLi (R^Si^ = SiMePh_2_) with 0.33 equivalents of PCl_3_ was carried out. Specifically, the treatment of a THF/Et_2_O solution of the bis(silyl)carbenoid, which was prepared *via* the lithiation of R^Si^_2_CBr_2_ using *t*-BuLi at −110 °C, with 0.33 equivalents of PCl_3_ in Et_2_O afforded phosphaalkene 1 in 31% yield without the formation of the expected product (A) (Scheme 1). Compound 1, which is air- and moisture-stable, was purified by column chromatography on SiO_2_ using CH_2_Cl_2_/hexane as the eluent. The molecular structure of 1 was characterized by spectroscopic techniques and single-crystal X-ray diffraction (SCXRD) analysis (Fig. 2).^19^ The ^31^P NMR chemical shift of 1 (436.5 ppm) is close to those of related phosphaalkenes (*ca.* 378–439 ppm).^20–22^ The theoretically estimated value of the chemical shift of 1 (*δ*_P_ = 470 ppm) based on gauge-independent atomic orbital (GIAO) NMR calculations is consistent with the experimental results,^23^ suggesting π-bond character for the C=P bond in solution and a negligible solvent effect. The P1–C1 bond length of 1 (1.675(2) Å) is almost identical to those of the related phosphaalkenes (*ca.* 1.66 Å)^20,21,24^ and considerably shorter than the P1–C2 bond of 1 (1.835(2) Å), suggesting a double- and single-bond character for the P1=C1 and P1–C2 bonds, respectively. In contrast to the tetrahedral geometry of the C2 atom, the C1 atom features a trigonal planar geometry with a bond-angle sum of 360° around the C1 atom. To elucidate the reaction mechanism, we examined the trapping reactions of the lithiated species generated *in situ* by the addition of CH_3_I in the reaction of R^Si^_2_CBr_2_ with *t*-BuLi.^25^ The treatment of R^Si^_2_CBr_2_ with 2.9 eq. of *t*-BuLi followed by the addition of an excess amount of CH_3_I resulted in the formation of three methylated compounds derived from the intermediates of bis(silyl)carbenoid 2, lithium bis(silyl)methanide 3, and dilithio compound 4 (Scheme S1†). The mechanism for the formation of 1 remains unclear at this stage, even though it seems feasible to assume that 1 is formed *via* the generation of *P*-chlorophosphaalkene 5 as a reactive intermediate, followed by its reaction with lithium bis(silyl)methanide 3 together with the elimination of LiCl (Scheme 2).

**Scheme 3:**
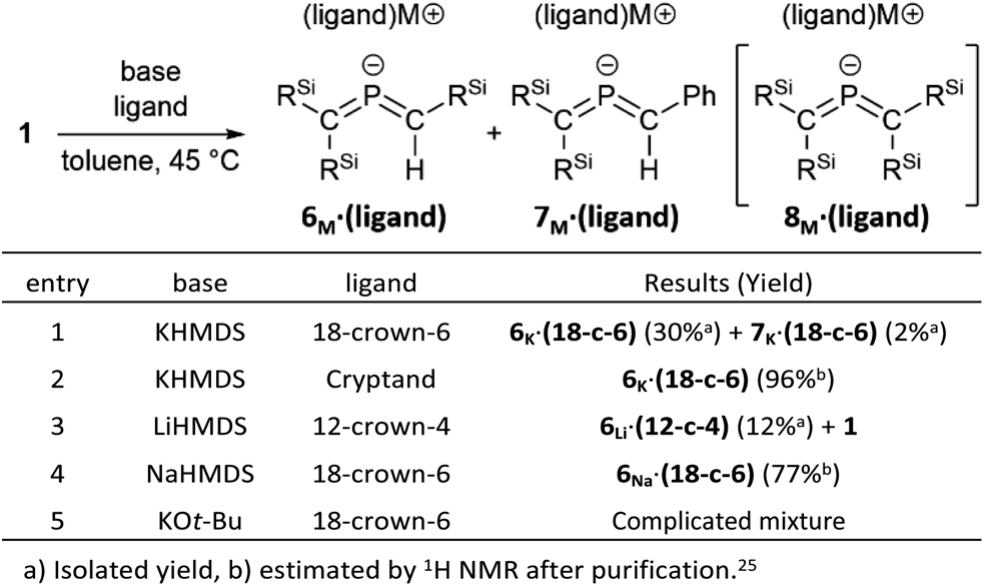
The synthesis of bis(methylene)-λ^5^-phosphane anions 6_M_·(ligand) and 7_M_·(ligand).

Subsequently, we attempted the deprotonation of 1 in the expectation of the formation of bis(methylene)-λ^5^-phosphane anion 8_K_·(18-C-6)*via* an E2-elimination using potassium hexamethyldisilazide (KHMDS) in the presence of 18-crown-6 in toluene. However, unexpectedly, desilylated compounds 6_K_·(18-c-6) (30%) and 7_K_·(18-c-6) (2%) were obtained as crystalline compounds (Scheme 3; entry 1). In the case of using a cryptand instead of 18-crown-6, only 6_K_·(cryptand) was formed (entry 2). Using LiHMDS or NaHMDS as a base also furnished 6_M_·(ligand) exclusively (entries 3 and 4). In contrast, when KO*t*-Bu in combination with 18-crown-6 was used instead of KHMDS/18-crown-6, a complicated mixture was obtained.

**Scheme 4:**
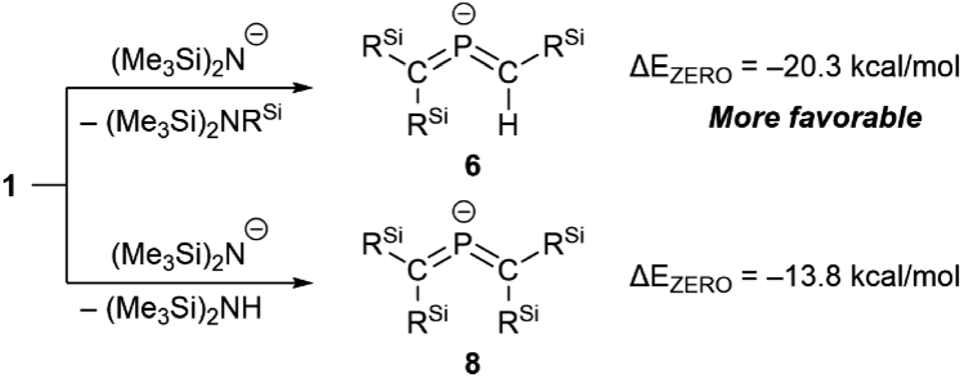
Comparison of Δ*E*_ZERO_ between the desilylation and deprotonation reactions of 1.

Accordingly, it can be concluded that it is crucial to use the HMDS anion for the generation of 6_M_·(ligand), and that the accompanied potassium cation causes the simultaneous formation of 7_K_·(ligand). The formation of 6_M_·(ligand) should most likely be interpreted in terms of a favorable nucleophilic attack of the HMDS anion on the electrophilic Si atom, even though the details of the mechanism for the formation of 7_K_·(ligand) remain unclear at this stage.^26^ As shown in Scheme 4, theoretical calculations suggested that the formation of 6 together with amino silane (Me_3_Si)_2_NSiMePh_2_ in the reaction of 1 with the HMDS anion should be thermodynamically more favorable (Δ*E*_ZERO_ = −20.3 kcal mol^−1^) compared to the reaction of phosphaalkene 1 with (Me_3_Si)_2_N^−^ to give tetrasilyl bis(met hylene)-λ^5^-phosphane anion 8 and (Me_3_Si)_2_NH (Δ*E*_ZERO_ = −13.8 kcal mol^−1^). To further investigate the reaction mechanism for the desilylation reaction, we performed theoretical calculations on the potential energy surface of both a deprotonation reaction and a desilylation reaction of the phosphaalkene (SM) using model compounds as shown in Fig. S55.† The reaction barrier for the deprotonation product *via*TS1 is smaller than that of the desilylation product *via*TS2, while the product of the desilylation reaction (Pr2) is significantly stable compared to that of the deprotonation reaction (Pr1). As a result, it was found that the formation of bis(methylene)-λ^5^-phosphane anion 6 is a thermodynamically favored reaction.

Bis(methylene)-λ^5^-phosphane anions 6_K_·(18-c-6) and 7_K_·(18-c-6) exhibit moderate thermal stability in the solid state (6_K_, m.p.: 93 °C (decomp); 7_K_, m.p.: 53 °C (decomp.)) and high thermal stability in solution, *i.e.*, the NMR spectra of 6_K_·(18-c-6) and 7_K_·(18-c-6) in C_6_D_6_ remained unchanged after 24 h at 80 °C.

**Fig. 3:**
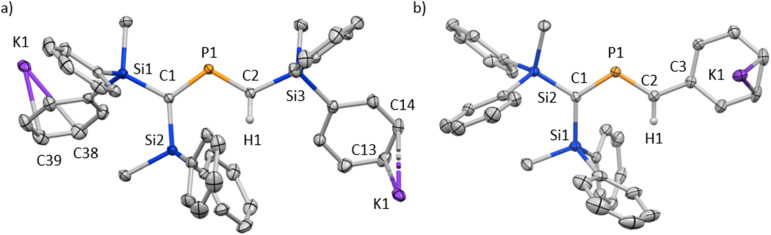
(a) Molecular structure of 6_K_·(18-c-6) in the crystalline state with thermal ellipsoids at 50% probability; all hydrogen atoms except for H1 and 18-crown-6 are omitted for clarity. Selected bond lengths [Å] and angles [°]: C1–P1 1.723(1), P1–C2 1.694(1), and C1–P1–C2 112.23(5). (b) Two independent molecules (7_K_-A and 7_K_-B) were found in the unit cell. Molecular structure of one of the two crystallographically independent molecules in the unit cell of 7_K_·(18-c-6) in the crystalline state with thermal ellipsoids at 50% probability; all hydrogen atoms except for H1 and 18-crown-6 are omitted for clarity. Selected bond lengths [Å] and angles [°] [7_K_-A]: C1–P1 1.717(2), P1–C2 1.690(3), C2–C3 1.449(3), C1–P1–C2 112.6(1) [7_K_-B], P2–C47 1.716(3), C48–P2 1.694(3), and C48–P2–C47 112.5(1).^19^

**Fig. 4:**
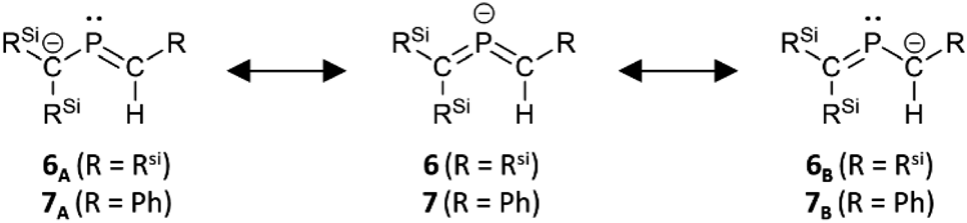
Canonical resonance structures of bis(methylene)-λ^5^-phosphane anions 6 and 7.

**Fig. 5:**
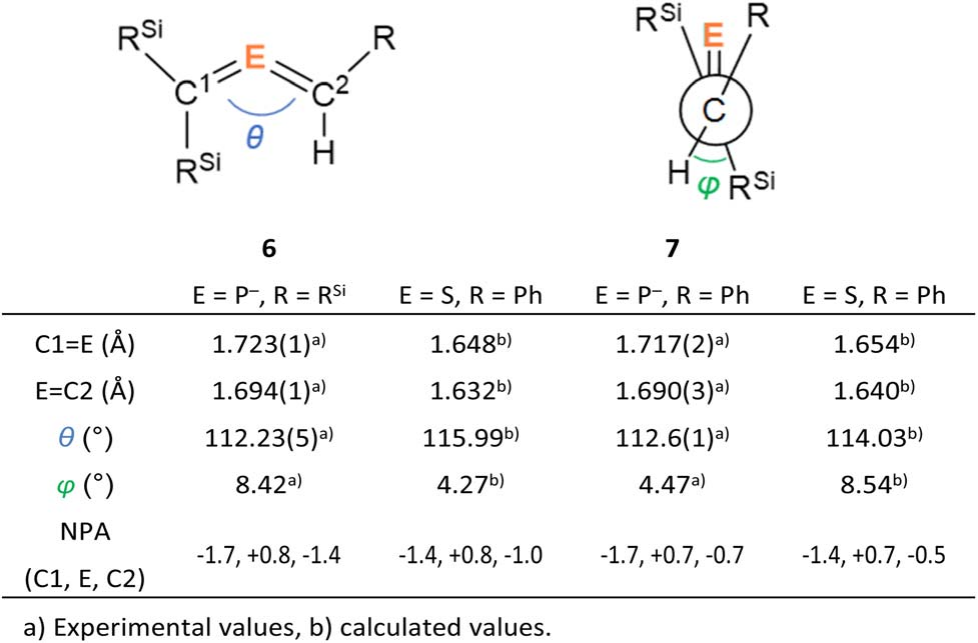
Comparison of structural parameters between bis(methylene)-λ^5^-phosphane anions and bis(methylene)-λ^4^-sulfanes.

The characterization of 6_K_·(18-c-6) and 7_K_·(18-c-6) was accomplished by multinuclear NMR and UV-vis spectroscopy, mass spectrometry, and SCXRD analysis.^19^ In the molecular structures of 6_K_·(18-c-6) and 7_K_·(18-c-6) (Fig. 3), both the protons on C2 of 6_K_·(18-c-6) and 7_K_·(18-c-6) were located based on the residual Q-peaks, which represent residual electron density peaks in the differential electron-density map, in the LSQ process (see the ESI†). In the crystal structure of 6_K_·(18-c-6), the potassium cation is coordinated by an 18-crown-6 ether and two phenyl rings, which results in the formation of an infinite chain structure in the solid state (Fig. 3a and S42†). In contrast, in 7_K_·(18-c-6), the potassium cation is coordinated by an 18-crown-6 ether and one phenyl ring connected to the allene moiety, resulting in a monomeric structure. It should also be noted here that the phenyl ring is co-planar to the C=P=C moiety, probably due to π-conjugation, which is indicative of the considerable π-bond character of both C=P bonds. The central C=P=C moiety in 6_K_·(18-c-6) is bent (C1–P1–C2: 112.23(5)°) with almost identical C=P bond lengths (C1–P1: 1.723(1) Å; P1–C2: 1.694(1) Å). In the case of 7_K_·(18-c-6), the allene moiety also shows a bent allene-type structure (C1–P1–C2: 112.6(1)°; C1–P1: 1.717(2) Å; P1–C2: 1.690(3) Å). The C–P bonds are considerably shorter than typical C–P single bonds (*e.g.*, 1.835(2) Å in 1) but slightly longer than typical C=P double bonds (*e.g.*, 1.675(2) Å in 1). The fact that the C1 = P1 bond (1.723(1) Å) is slightly longer than the C2 = P1 bond (1.694(1) Å) should most likely be rationalized in terms of the predominant contribution of the resonance structure of bis(methylene)-λ^5^-phosphane anion 6 bearing the C=P=C allenic π-bonds along with the partial contributions of 2-phosphapropenyl anion 6_A_ rather than 6_B_ (Fig. 4), wherein the anion charge is partially localized on the C1 atom probably due to the considerable α-effect of the two adjacent silyl groups. The C1–P1 bonds (1.716(3) Å and 1.717(2) Å) in 7_K_·(18-c-6) are slightly longer than the P1–C2 bonds (1.694(3) Å and 1.690(3) Å) and those of 6_K_·(18-c-6). These structural features are similar to those of a previously reported bis(methylene)-λ^4^-sulfane.^12^ In contrast, as shown in Fig. 5, the dihedral angles (*φ*) between the two terminal carbon planes of the allene moieties in 6_K_·(18-c-6) (8.4°) and 7_K_·(18-c-6) (4.5°/3.4°) are very small, suggesting an almost coplanar geometry, which is different from that of the reported bis(methylene)-λ^4^-sulfane (51.9°). Theoretical calculations indicated that the dihedral angles between the terminal carbon planes of the allene moieties (*φ*) tend to increase with increasing steric demand of the substituents on the terminal carbons (Fig. S46†). Thus, it can be concluded that a bis(methylene)-λ^5^-phosphane anion should exhibit an intrinsically coplanar geometry. Moreover, the C1–P1–C2 bond angles of 6_K_·(18-c-6) (112.23(5)°) and 7_K_·(18-c-6) (112.6(1)°) are almost the same as that of 1 (112.6(1)°), but significantly narrower than those of the hitherto reported bis(methylene)-λ^5^-phosphanes (127–137°),^7^ indicating high s-character for the lone pair on the phosphorus atoms of 6_K_·(18-c-6) and 7_K_·(18-c-6) as well as high p-character of R–P(=C)_2_ σ-/π-bonds.

**Fig. 6:**
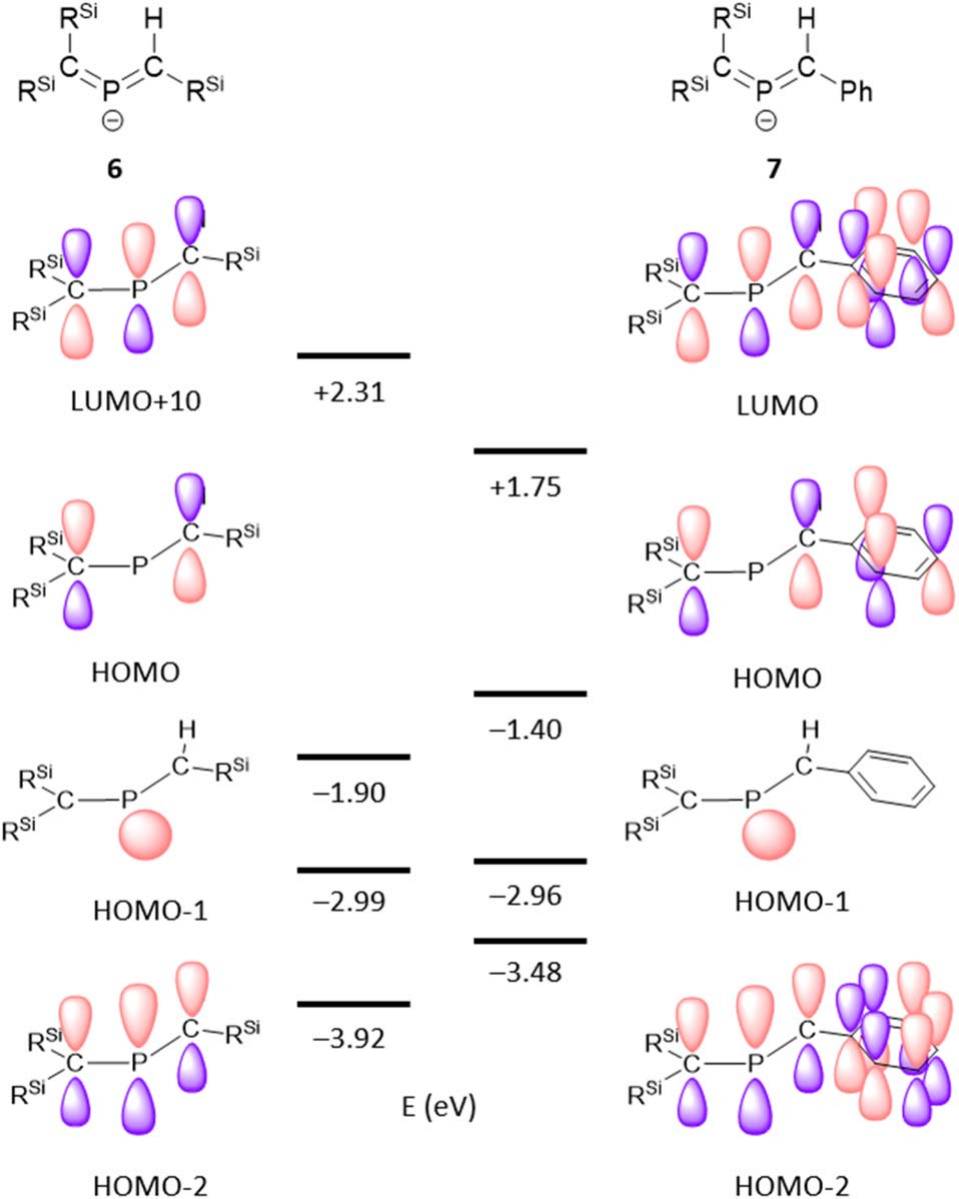
Kohn–Sham orbitals of 6 (left) and 7 (right), calculated at the B3PW91-D3(bj)/6-311G(3d) level.

The structural optimization of bis(methylene)-λ^5^-phosphane anions 6 and 7 using DFT calculations^23^ was able to closely reproduce the experimentally observed structures (Fig. S43 and S44†). Natural-bond-orbital (NBO) calculations on the optimized structure of 6 showed one lone pair at the P atom (HOMO−1), two C–P σ-bonds, and a 3-center-4-electron π-bond on the C–P–C moiety as the LUMO+10 (anti-bonding), HOMO (π*(P=C)), and HOMO−2 (π(P=C)) (Fig. 6).^23^ On the other hand, the 3-center-4-electron π-bond in 7 is composed of the LUMO (anti-bonding), HOMO (non-bonding), and HOMO−2 (bonding), whereby the HOMO–LUMO gap is narrowed by the π-conjugation with the attached phenyl group. The estimated bond orders of the C–P bonds in 6 and 7, based on their Wiberg bond indices (WBIs) of 1.48 (P1–C2) and 1.24 (C1–P1) for 6, as well as 1.45 (P1–C2) and 1.24 (C1–P1) for 7, are slightly smaller than that of the C=P double bond in 1 (1.66) and larger than the value for the C–P single bond in 1 (0.91), indicating π-bonding character for the C–P bonds in 6 and 7. The calculated natural population analysis (NPA) charge on the phosphorus atom was +0.8 for 6 and +0.7 for 7, while the charges on the adjacent carbon atoms were −1.7 on C1 for 6 and 7 and −1.4 on C2 for 6 and −0.7 for 7. The NPA charge distribution on 6 and 7 was similar to those of bis(methylene)-λ^4^-sulfanes, which have an isoelectronic relationship with the bis(methylene)-λ^5^-phosphane anions (Table 1). Considering the aforementioned results in their entirety, it should be concluded that the overall structure of bis(methylene)-λ^5^-phosphane anion 6 is characterized by not only 3-center-4-electron π-bonds in the C=P=C allene bonding but also a partial contribution of resonance structure 6_A_ rather than 6_B_.

**Table 1:** Comparison of NMR spectral data for 6_K_·(18-c-6) and 7_K_·(18-c-6)

	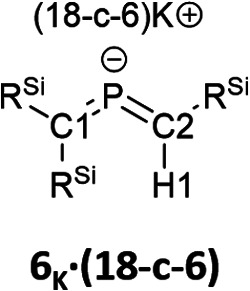	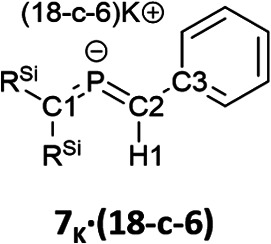	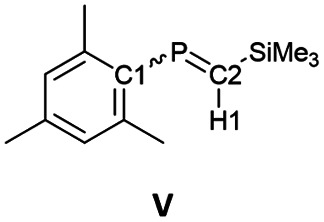	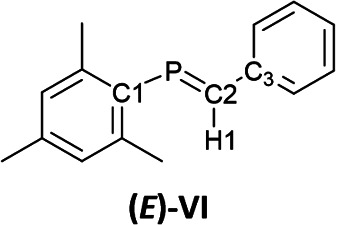
*δ* ^31^P	339.0	306.8	326.3, 334.1	256.6

***δ*** ^**13**^ **C**				
C1	75.0	72.4	—a, 141.2	139.0
(^1^*J*_C−P_ in Hz)	(82)	(74)	(—a, 67)	(54)
C3	108.1	128.0	177.8, 173.2	175.9
(^1^*J*_C−P_ in Hz)	(68)	(44)	(53, 66)	(35)
C3	—	121.2	—	140.2
(^1^*J*_C−P_ in Hz)	—	(19)	—	(14)

***δ*** ^**1**^ **H**				
H1	6.08	—b	7.89, 7.77	8.21
(^2^*J*_H−P_ in Hz)	(16.8)	(14.7)^c^	(25, 18)	(26)

a Unidentified.

b Could not be identified due to overlapping.

c ^2^ *J*_P−H_ in Hz.

The ^31^P NMR spectra of 6_K_·(18-c-6) in *o*-difluorobenzene and 7_K_·(18-c-6) in benzene-*d*_6_ at room temperature showed at 339.0 ppm for 6_K_·(18-c-6) and at 306.8 ppm for 7_K_·(18-c-6), which were significantly low-field shifted compared to that of potassium diphenylphosphide, Ph_2_PK (−10.0 ppm), but slightly high-field shifted compared to that of 1 (*δ* = 436.5). These chemical shifts thus fall within the reported range for bis(methylene)-λ^5^-phosphanes (120–347 ppm).^9^ The ^31^P NMR chemical shifts estimated by the GIAO calculations for 6 (350 ppm) and 7 (317 ppm) are consistent with the experimental results (for details, see the ESI†).^23^

The ^13^C NMR spectrum of 6_K_·(18-c-6) in *o*-difluorobenzene at 333 K showed two signals at 75.0 ppm (C1) and 108.1 ppm (C2). These values fall within the reported range for bis(methylene)-λ^5^-phosphanes (31.6–122.3 ppm).^9^ Furthermore, the phosphorus-carbon coupling constants (^1^*J*_C−P_ = 82 Hz for C1 and 68 Hz for C2) and 7_K_·(18-c-6) (^1^*J*_C−P_ = 74 Hz for C1 and 44 Hz for C2) are larger than those of previously reported bis(methylene)-λ^5^-phosphanes (25.6–74.7 Hz), phosphaalkenes^27^V and (*E*)-VI (^1^*J*_C−P_ = 34.5–78.1 Hz for >C=P– species), and those of the C–P single bond in V and (*E*)-VI (Table 1). This result indicates that the C–P bonds in 6_K_·(18-c-6) and 7_K_·(18-c-6) exhibit multiple-bond character with high p-character.

The ^1^H NMR spectrum of 6_K_·(18-c-6) showed that the proton on C2 (6.08 ppm) is significantly high-field shifted compared to that in 1 (3.72 ppm), indicating an increase in the effect of magnetic anisotropy due to the π-electrons of 6_K_·(18-c-6). The proton on C2 in 7_K_·(18-c-6) could not be observed due to significant overlap with the phenyl protons. The P–H coupling constant in 6·(18-c-6) (^2^*J*_H−P_ = 16.8 Hz) is larger than that in (Me_3_Si)_2_CHPCl_2_ (14.3 Hz) but smaller than those of phosphaalkenes V and (*E*)-VI (18–26 Hz; Table 1).

The UV-vis spectra of 6_K_·(18-c-6) in benzene and 7_K_·(18-c-6) in toluene at room temperature exhibited characteristic adsorptions at *λ*_max_ = 378 nm (*ε* = 7.8 × 10^3^ L mol^−1^ cm^−1^) and 474 nm (*ε* = 1.1 × 10^4^ L mol^−1^ cm^−1^), respectively. Time-dependent DFT calculations for 6 and 7 showed excitation energies of *λ* = 408 nm and 470 nm (Fig. S13 and S19†), respectively, for the HOMO–LUMO electron transitions (π–π*), indicating a bathochromic shift due to the π-conjugation of the phenyl group in 7_K_·(18-c-6). Taking these experimental and theoretical investigations into account, it can be concluded that both 6 and 7 contain two C=P π-bonds, *i.e.*, they should behave in solution as bent allene-type compounds with cumulative C=P π bonds.

**Scheme 5:**
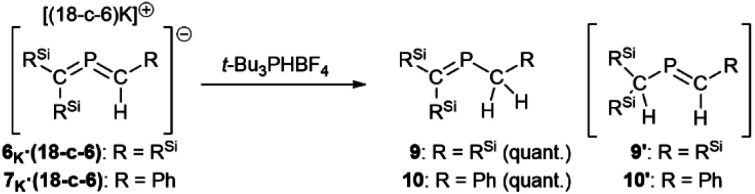
Protonation of 6_K_ and 7_K_ with *t*-Bu_3_PHBF_4_.

Finaly, reactions of 6_K_·(18-c-6) and 7_K_·(18-c-6) with *t*-Bu_3_PHBF_4_ as a protonating reagent were performed to investigate the nucleophilicity/basicity of the allene moieties as indicated by the canonical structures shown in Fig. 4. Both reactions proceeded selectively to produce the corresponding proton adducts 9 and 10 (Scheme 5). Although the anionic charges of 6 and 7 can be expected to be predominantly located on their C1 atoms as resonance structures 6_A_ and 7_A_, the protonation occurred at their C2 atoms, suggesting that the negative charges on the C1 atoms are significantly stabilized by the double-silyl-α-effect, and thus the C2 atoms should be more basic than the C1 atoms due to their reactive C=P π-bond character. In addition, the theoretical calculations of the products indicated that 9 is thermodynamically more stable than 9′ by Δ*E*_Zero_ = 3.4 kcal mol^−1^ (Fig. S47†), which would support the selective formation of the C2-protonated products 9 and 10.

## Conclusions

In summary, the first isolable bis(methylene)-λ^5^-phosphane anions (6_K_·(18-c-6) and 7_K_·(18-c-6)) were synthesized by the desilylation of the corresponding phosphaalkene with KHMDS. Spectroscopic and single-crystal X-ray diffraction analyses in combination with theoretical calculations revealed that 6 and 7 show a bent and planar allene structure with two cumulative P=C π-bonds in the C=P=C allene moiety forming a so-called three-center-four-electron π-bond. Further investigations into the reactivity of 6 and 7 are currently in progress in our laboratories and the results will be disclosed elsewhere in due course.

## Data availability

The data supporting this article have been included as part of the ESI.†

## Author contributions

The project was designed and conducted by K. S. and T. S. Experimental work such as synthesis and characterization was carried out by A. N. All authors contributed to writing the manuscript.

## Conflicts of interest

There are no conflicts to declare.

## Supplementary Material

SC-OLF-D4SC07246D-s001

SC-OLF-D4SC07246D-s002
