# Supplementary material for: Bis(methylene)-λ5-phosphane anions
Source: Chem Sci. 2024 Nov 14;15(48):20509–14. doi: 10.1039/d4sc07246d (PMC11583925; doi:10.1039/d4sc07246d)
Supplement: SC-015-D4SC07246D-s002 [file SC-015-D4SC07246D-s002.pdf]

Supplementary Information (SI) for Chemical Science.
This journal is © The Royal Society of Chemistry 2024

Supporting Information for

Bis(methylene)-λ^5-phosphane Anions

Akihiro Nomoto,[a] Koh Sugamata,*[b] and Takahiro Sasamori*[a,c,d]

a. Graduate School of Science and Technology, University of Tsukuba 1-1-1 Tennoudai, Tsukuba, Ibaraki 305-8571, Japan
b. Department of Chemistry, College of Science, Rikkyo University 3-34-1 Nishi-Ikebukuro, Toshima-ku, Tokyo 171-8501, Japan
c. Division of Chemistry, Institute of Pure and Applied Sciences, University of Tsukuba 1-1-1 Tennoudai, Tsukuba, Ibaraki 305-8571, Japan
d. Tsukuba Research Center for Energy Materials Sciences (TREMS), University of Tsukuba 1-1-1 Tennoudai, Tsukuba, Ibaraki 305-8571, Japan

E-mail: sugamata@rikkyo.ac.jp, sasamori@chem.tsukuba.ac.jp
Tel, Fax: +81-29-853-4412

Table of Contents

General Remarks 2
Experimental Procedure 2
X-Ray Crystallographic Analysis 26
Theoretical Calculations 28
Natural-Resonance-Theory (NRT) calculations [S6] 31
Estimation for ^{31}P NMR chemical shifts 32
Estimation of the rotation barriers for 6a and 7a 32
References 38

General Remarks

All manipulations were carried out under an argon atmosphere using either Schlenk line techniques or glove boxes. All solvents were purified by standard methods. Trace amounts of water and oxygen remaining in the solvents were thoroughly removed by bulb-to-bulb distillation from potassium mirror prior to use. All the crystallization was performed at room temperature unless otherwise indicated. ^{1}H, ^{13}C{^{1}H}, and ^{29}Si{^{1}H}, ^{31}P{^{1}H}, ^{31}P NMR spectra were measured on a Bruker AVANCE-400 spectrometer (^{1}H: 400 MHz, ^{13}C: 101 MHz, ^{29}Si: 79.5 MHz, ^{31}P: 162 MHz) and Bruker AVANCE-600 spectrometer (^{13}C{^{1}H}: 151 MHz, ^{29}Si{^{1}H}: 119 MHz). Signals arising from residual protons $CHCl_3$(7.26 ppm), C_6D_5H (7.16 ppm) or $CDHCl_2$(5.32 ppm) and C_6D_6 (128.0 ppm) in C_6D_6 were used as the internal standards for the ^{1}H and ^{13}C NMR spectra, respectively. The signal arising from $SiMe_4$ (0.0 ppm) was used as an external standard for the ^{29}Si NMR spectra. The signal arising from 85%H_3PO_4 (0.0 ppm) or Ph_3P (5.4 ppm) was used as an external standard for the ^{31}P NMR spectra. High-resolution mass spectra (HRMS) were obtained from a JEOL JMS-T100LP (DART) mass spectrometer. UV-Vis spectra were recorded on a SHIMADZU UV-3150 UV-Vis-NIR spectrometer under an argon atmosphere in 1 cm quartz cells. All melting points were determined on a Büchi Melting Point Apparatus M-565 and are uncorrected.

Experimental Procedure

·Synthesis of phosphaalkene 1

A solution of $(Ph_2MeSi)_2CBr_2$[S1] (3.95 g, 6.97 mmol) in THF (40 mL) and Et_2O (20 mL) at –110 ºC was treated with *t*-BuLi (12.0 mL, 1.69 M in pentane, 20.3 mmol). After stirring for 1 min at this temperature, a solution of PCl_3 in Et_2O (4.0 mL, 0.57 M in ether, 2.29 mmol) was added, and the reaction mixture was allowed to warm to room temperature over 1 h. All volatiles were removed under reduced pressure, yielding a crude red oil. The obtained oil was purified by column chromatography (SiO_2, hexane/DCM) to afford an orange oil, which was subsequently reprecipitated to give pure **1** in the form of pale-yellow crystals (615 mg, 0.728 mmol, 31% yield).

1: pale-yellow crystals, Mp. 153–154 ºC. ^{1}H NMR (400 MHz, CD_2Cl_2) δ 0.18 (s, 6H), 0.23 (s, 3H), 0.25 (s, 3H), 3.72 (s, 1H), 6.98 (dd, J = 8.2 Hz, J = 1.4 Hz, 4H), 7.11 (dd, J = 8.0 Hz, J = 1.2 Hz, 4H), 7.15-7.40 (m, 32H); ^{1}H NMR (400 MHz, C_6D_6) δ 0.31 (s, 6H), 0.46 (s, 3H), 0.47 (s, 3H), 4.00 (s, 1H), 7.05-7.29 (m, 32H), 7.49 (dd, J = 8.0 Hz, J = 1.2 Hz, 4H), 7.54-7.59 (m, 4H); ^{13}C{^{1}H} NMR (151 MHz, C_6D_6) δ –1.4 (CH_3), –0.2 (CH_3), 0.9 (CH_3), 1.0 (CH_3), 39.6 (d, J_{CP} = 94 Hz, CH), 127.8 (CH), 127.8 (CH),127.9 (CH), 128.2 (CH), 128.4 (CH), 129.0 (CH), 129.2 (CH), 129.5 (CH), 135.8 (CH), 136.0 (CH), 136.1 (CH), 136.4 (CH), 136.8(C), 137.4 (C), 139.2 (C), 139.6 (C), 178.0 (d, J_{CP} = 92 Hz, C); ^{29}Si{^{1}H} NMR (79.5 MHz, C_6D_6) δ –19.1 (d, J_{SiP} = 11 Hz), –12.4 (d, J_{SiP} = 33 Hz), –11.7 ; ^{31}P{^{1}H} NMR (162 MHz, C_6D_6) δ 436.5 (s); HRMS (DART-positive), *m/z*: Found: 845.3053 ($[M+H]^+$), calcd. for $C_{54}H_{53}PSi_4$ ($[M+H]^+$): 845.3040; UV/vis (benzene), 361 nm (ε = 6.1×10^{2}).

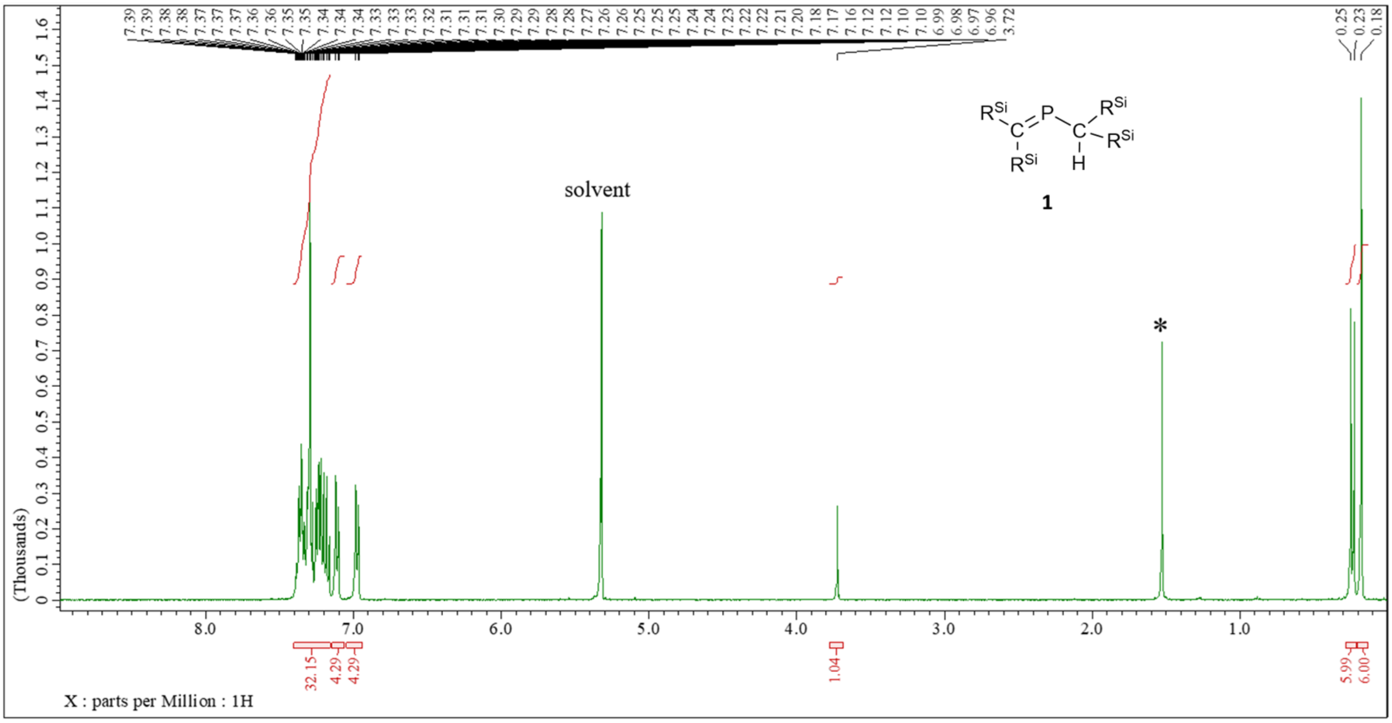

Figure S1. ^{1}H NMR spectrum of **1** in CD_2Cl_2. (*H_2O)

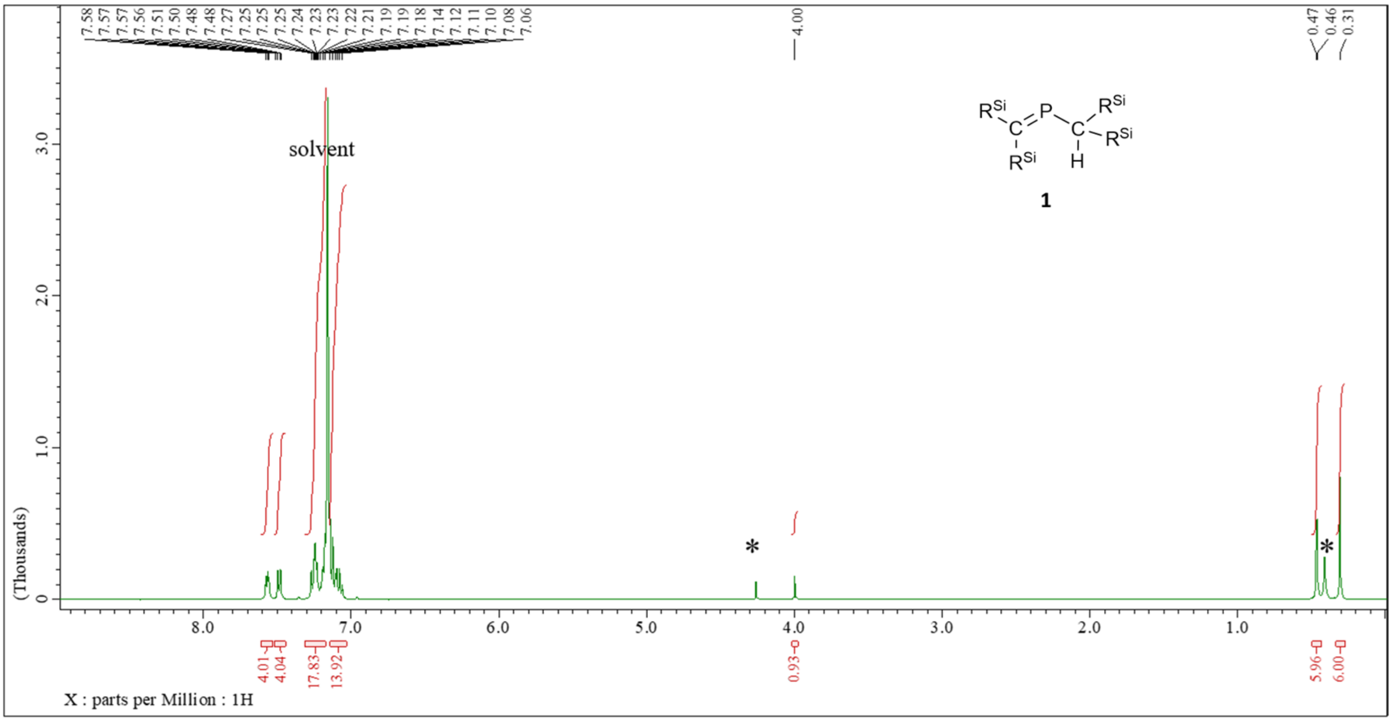

Figure S2. ^{1}H NMR spectrum of **1** in C_6D_6. (*H_2O/DCM)

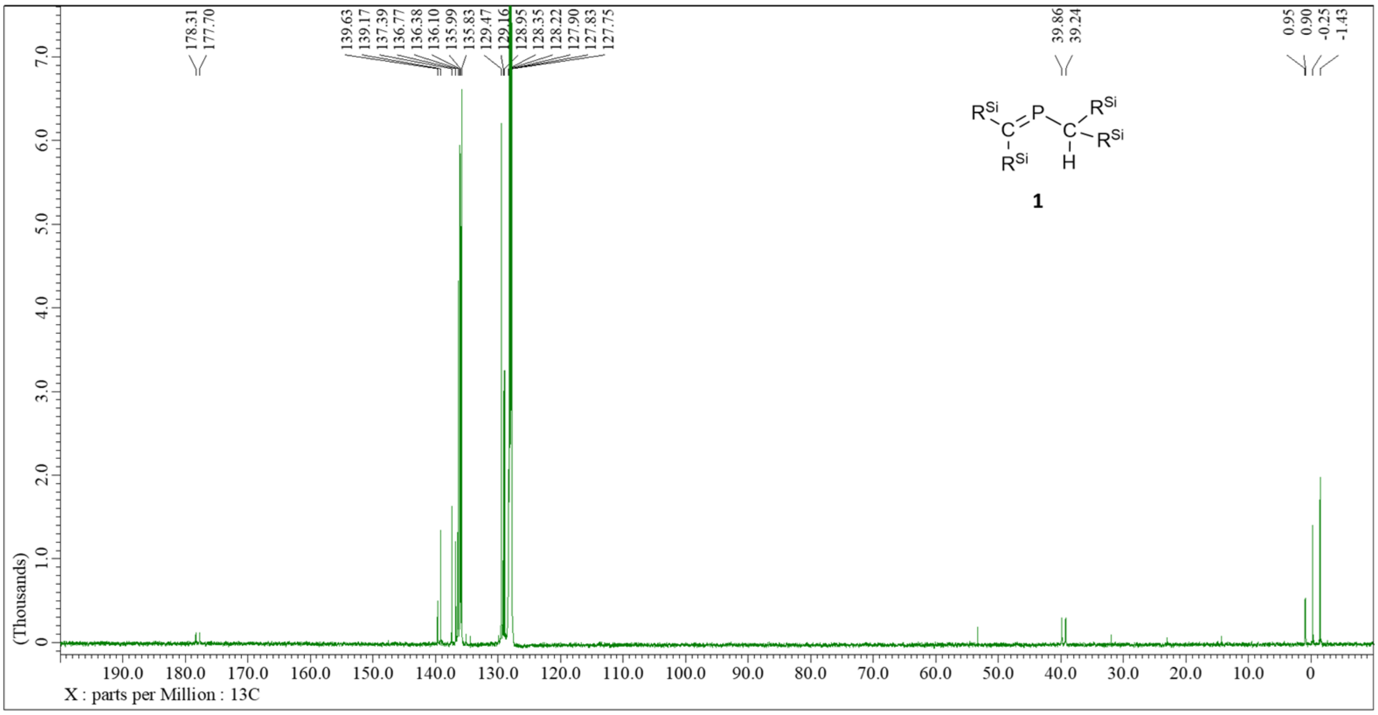

Figure S3. $^{13}C\{^{1}H\}$ NMR spectrum of **1** in C_6D_6.

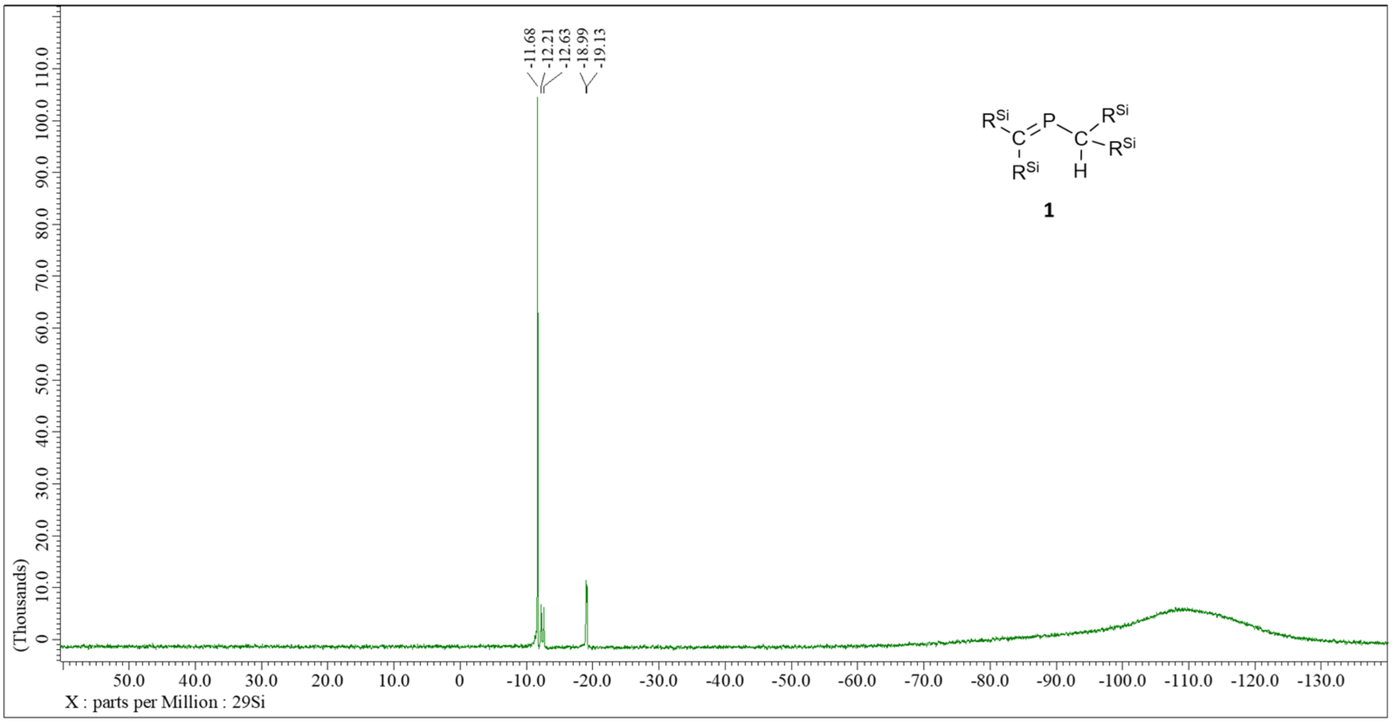

Figure S4. $^{29}Si\{^{1}H\}$ NMR spectrum of **1** in C_6D_6.

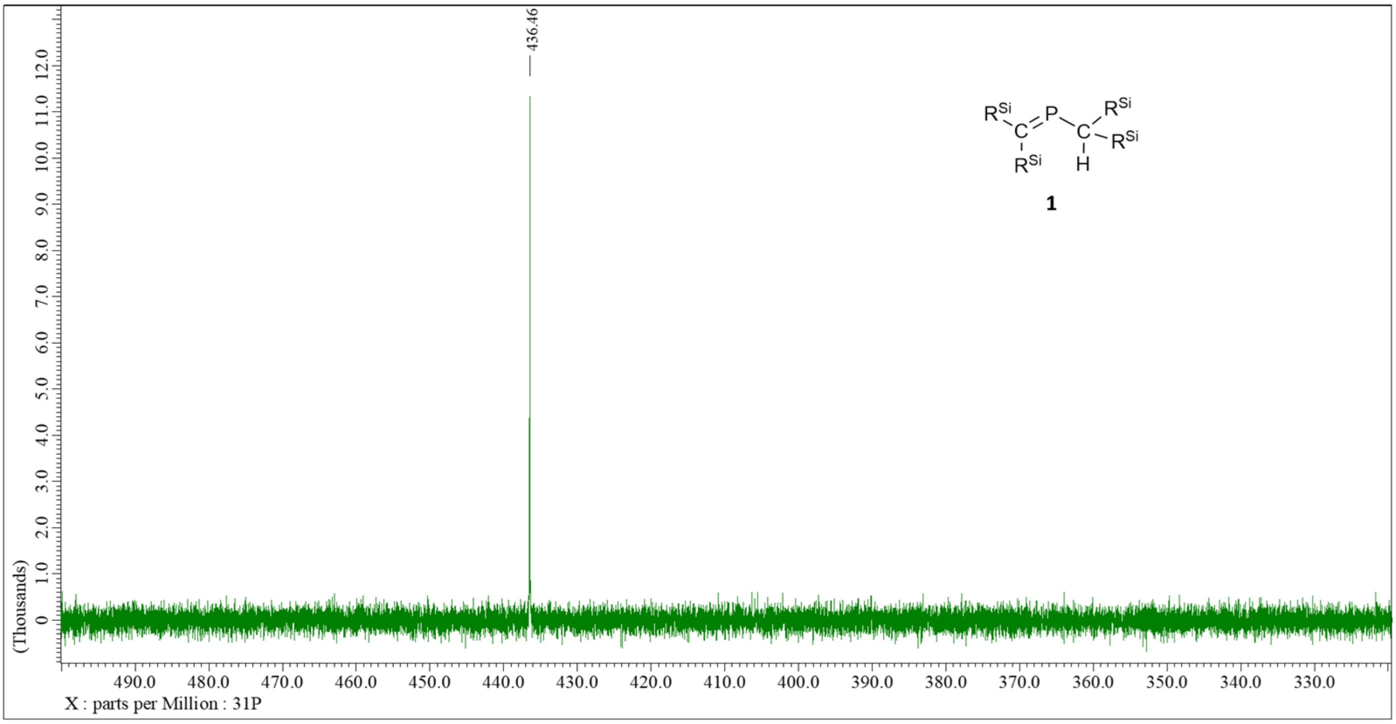

Figure S5. $^{31}P\{^1H\}$ NMR spectrum of **1** in C_6D_6.

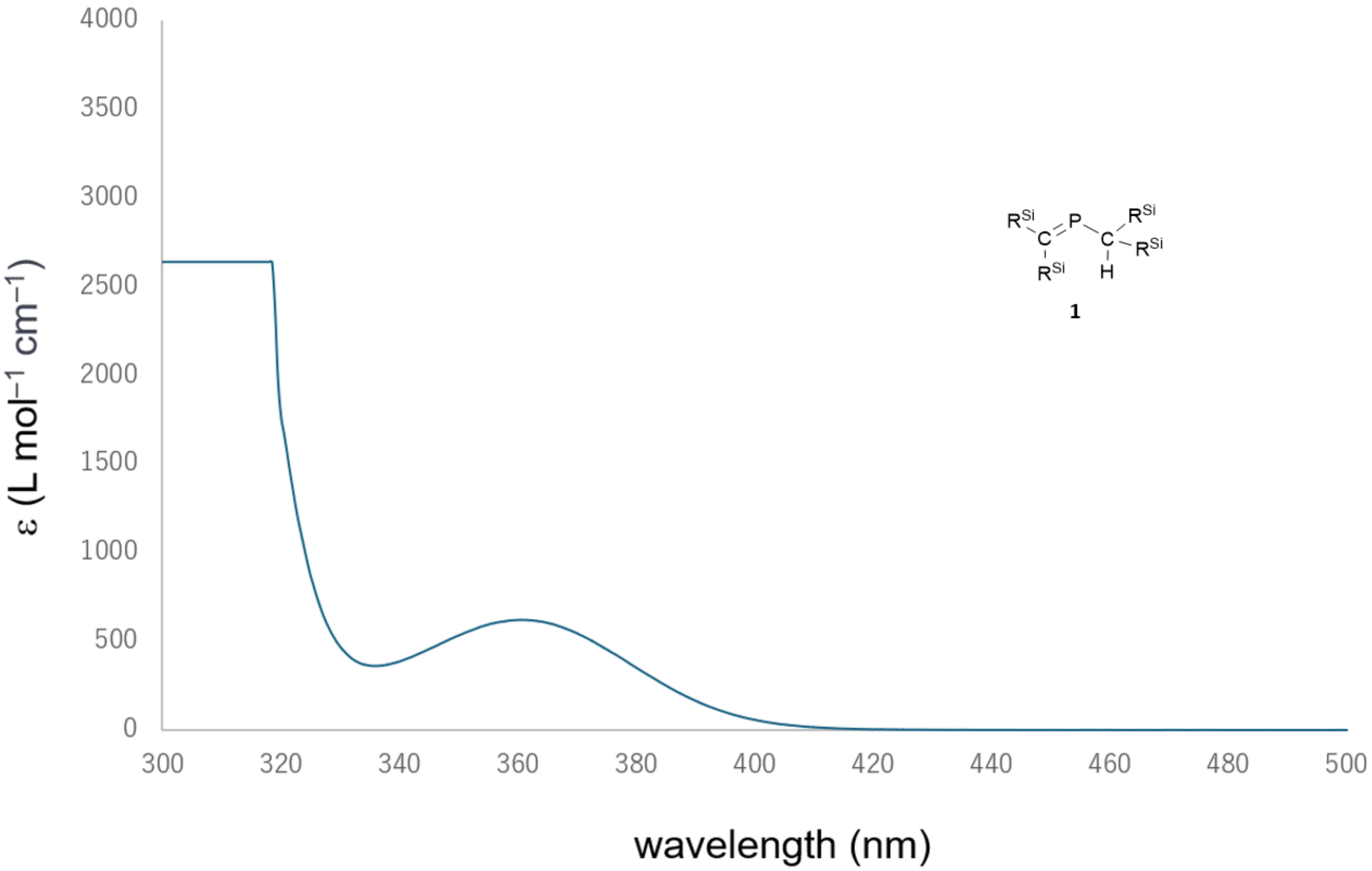

Figure S6. UV-vis spectrum of **1** in benzene (1.9×10^{-3} mol·L^{-1}) at room temperature.

・Trapping reaction of intermediates of the corresponding lithiated compounds generated by the reaction of $(Ph_2MeSi)_2CBr_2$ with *t*-BuLi

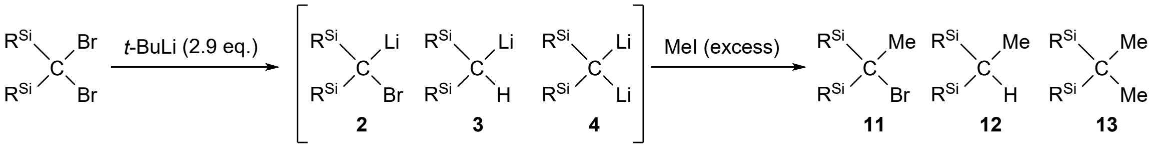

Scheme S1. Trapping reaction of lithiated compounds with MeI.

A mixture of THF (20 mL) and ether (10 mL) solution of $(Ph_2MeSi)_2CBr_2$ (1.98 g, 3.49 mmol) at –110 ºC was treated with *t*-BuLi (6.0 mL, 1.69 M in pentane, 10.1 mmol). After 1 min of stirring at this temperature, MeI (1.3 mL, 20.9 mmol) was added, and the reaction mixture was allowed to warm up to room temperature. The volatile materials of the solution were evaporated to afford the crude product. Three methylated compounds **11-13** were assigned to a 1:1:1 generation ratio as judged by the ^{1}H NMR spectra, as shown Figure S7.

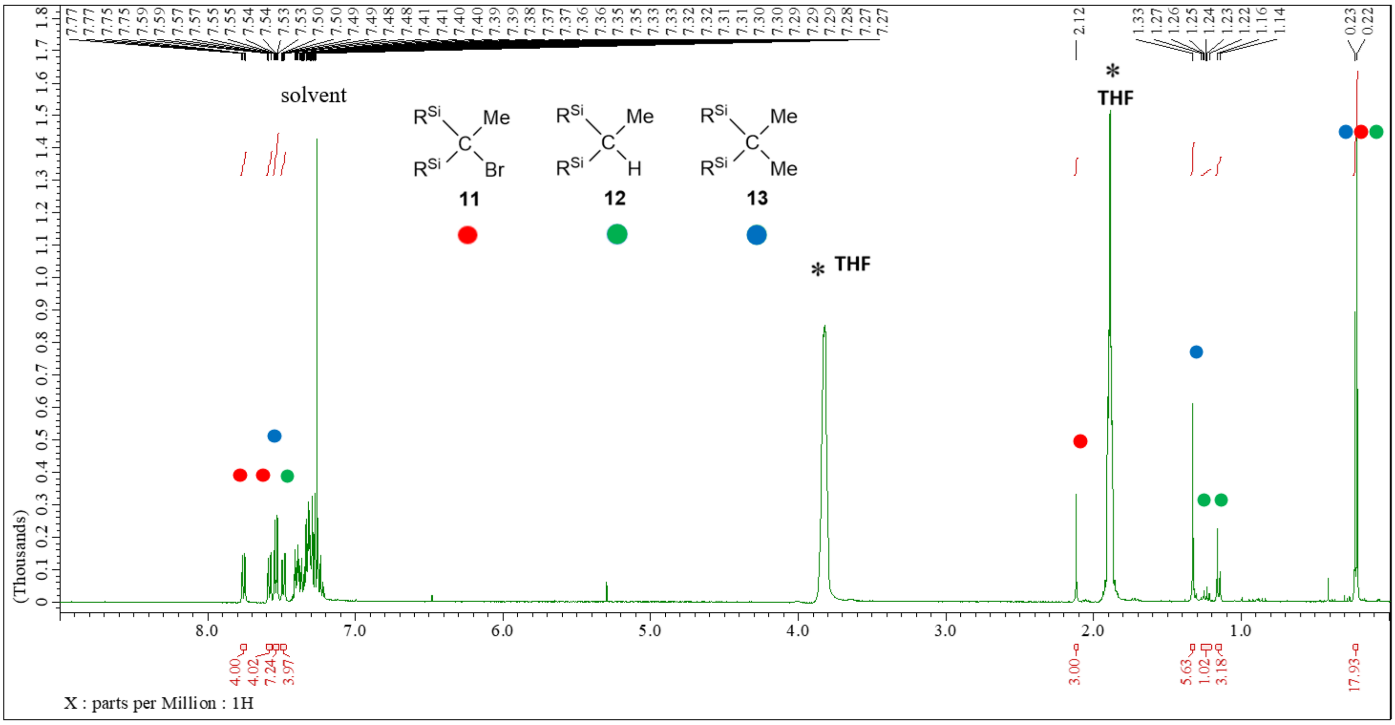

Figure S7. ^{1}H NMR spectrum of crude product in $CDCl_3$.

·Synthesis of bis(methylene)-λ^5-phosphane anion 6_K·(ligand) and 7_K·(ligand)

A mixture of **1** (550 mg, 0.651 mmol) and 18-crown-6 (344 mg, 1.30 mmol) in a J Young Schlenk bottle was dissolved in toluene (5.0 mL) at 45 °C. Then, a toluene solution of KHMDS (1.8 mL, 0.5 M in toluene, 0.9 mmol) was added dropwise, before the reaction mixture was stirred at 45 °C for 90 h. Subsequently, stirring was stopped, and the upper layer was removed. Fresh toluene was added to the flask, resulting in a phase separation; the upper layer was removed, and this procedure was repeated twice, before all volatiles were then removed under reduced pressure. The addition of toluene to the thus obtained residue yielded an orange powder that was reprecipitated to afford bis(methylene)-λ^5-phosphane anion **6_K·(18-c-6)** as a pale yellow solid (183 mg, 0.192 mmol, 30% yield). The filtrate was reprecipitated to afford bis(methylene)-λ^5-phosphane anion **7_K·(18-c-6)** as red crystals (10.3 mg, 0.0124 mmol, 2% yield).

6_K·(18-c-6): Mp. 93 ºC (dec.). ^{1}H NMR (400 MHz, *o*-difluorobenzene) δ 0.59 (br, 6H), 0.66(3H), 3.26 (s, 24H), 6.08 (d, J_{PH} = 16.8 Hz, 1H), 6.57-7.12 (m, 18H), 7.50-7.55 (m, 4H), 7.99 (br, 8H); ^{13}C{^{1}H} NMR (151 MHz, *o*-difluorobenzene, 333 K) δ –1.4 (d, J_{CP} = 14 Hz, CH_3), 3.1 (d, J_{CP} = 10 Hz, CH_3), 70.1 (CH_2), 75.0 (d, J_{CP} = 82 Hz, C), 108.1 (d, J_{CP} = 68 Hz, CH), 126.8 (CH), 126.8 (CH), 126.9 (CH), 127.0 (CH), 135.4(CH), 136.5(CH), 145.7(C), 145.8(C); ^{29}Si{^{1}H} NMR (79.5 MHz, C_6D_6) δ –16.5, –16.0; ^{31}P{^{1}H} NMR (162 MHz, *o*-difluorobenzene) δ 339.0 (s); HRMS(DART-negative), *m/z*: Found: 647.2190 ([6_K]$^-$), calcd. For $C_{41}H_{40}PSi_3$ ([6_K]$^-$): 647.2175; UV/vis (benzene), 378 nm (ε = 7.8×10^3).

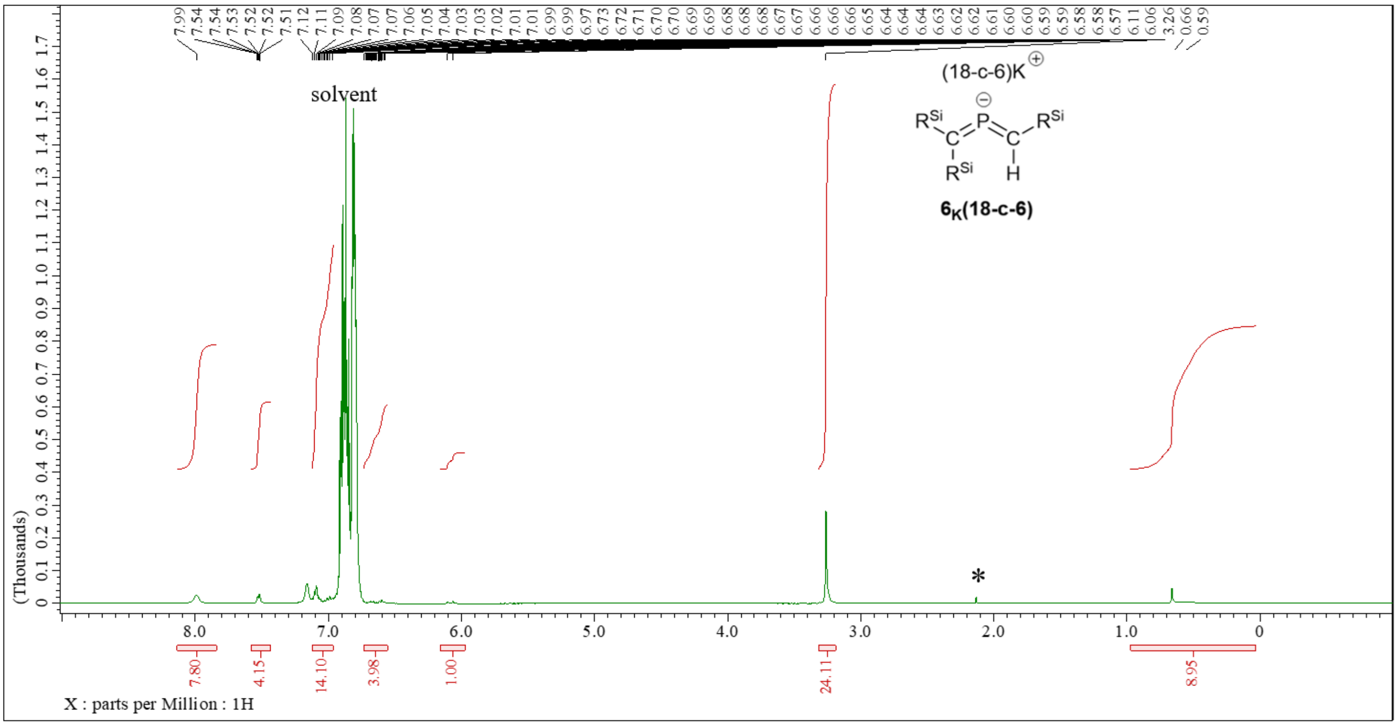

Figure S8. ^{1}H NMR spectrum of **6_K·(18-c-6)** in *o*-difluorobenzene. (*toluene)

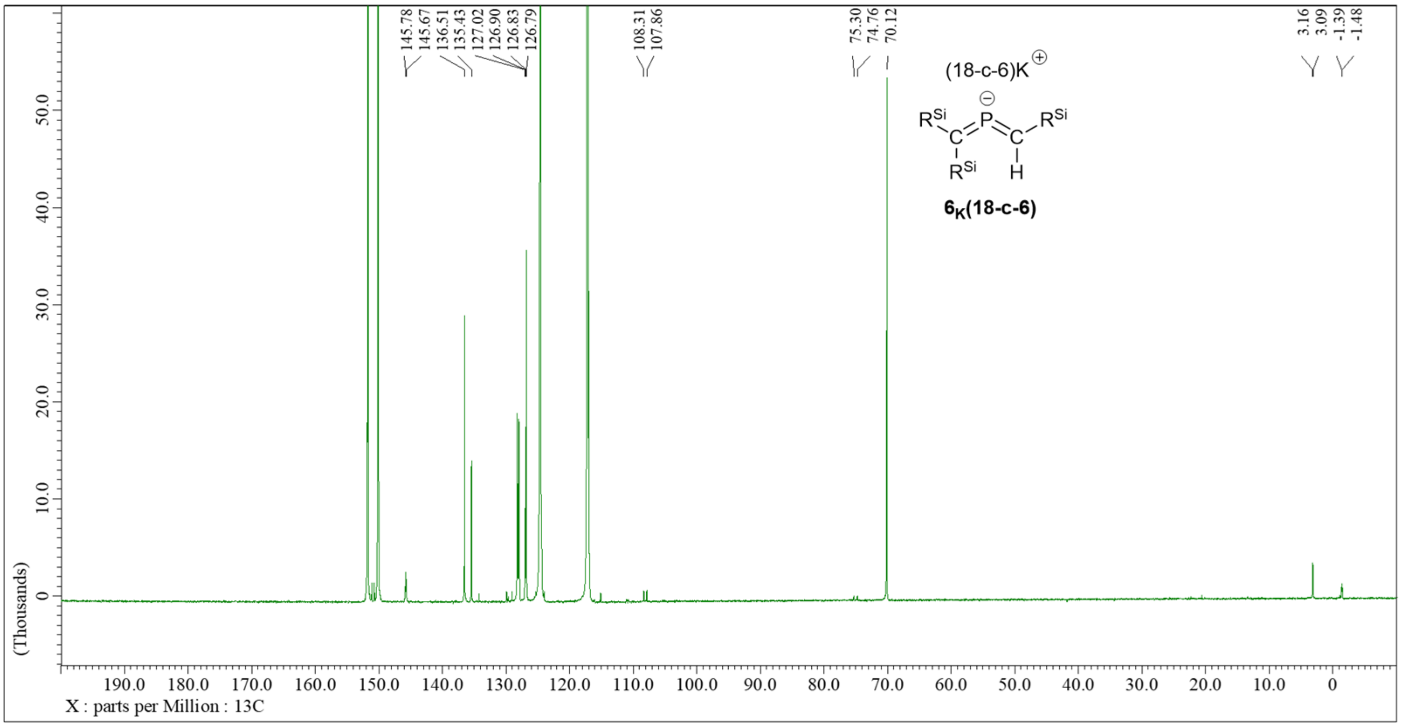

Figure S9. ^{13}C{^{1}H} NMR spectrum of **6_K·(18-c-6)** in *o*-difluorobenzene.

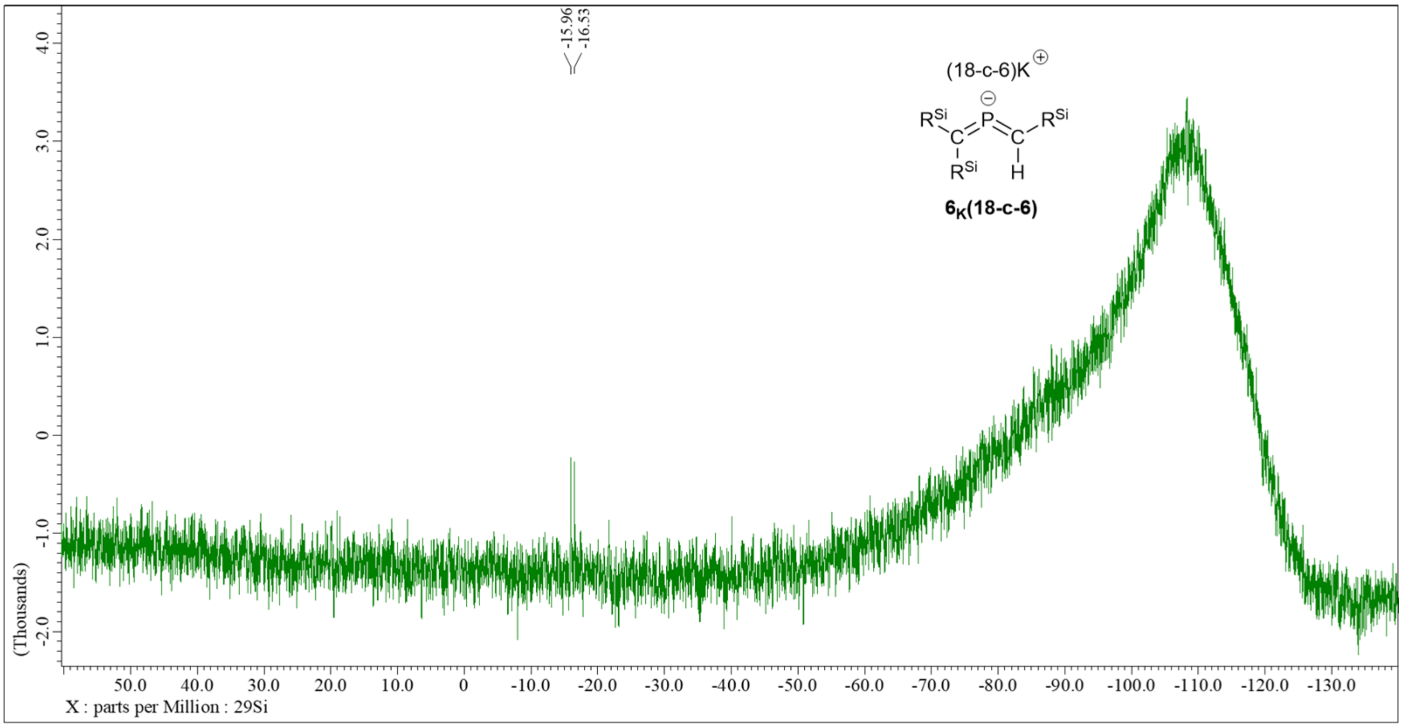

Figure S10. $^{29}Si\{^{1}H\}$ NMR spectrum of **6_K·(18-c-6)** in *o*-difluorobenzene.

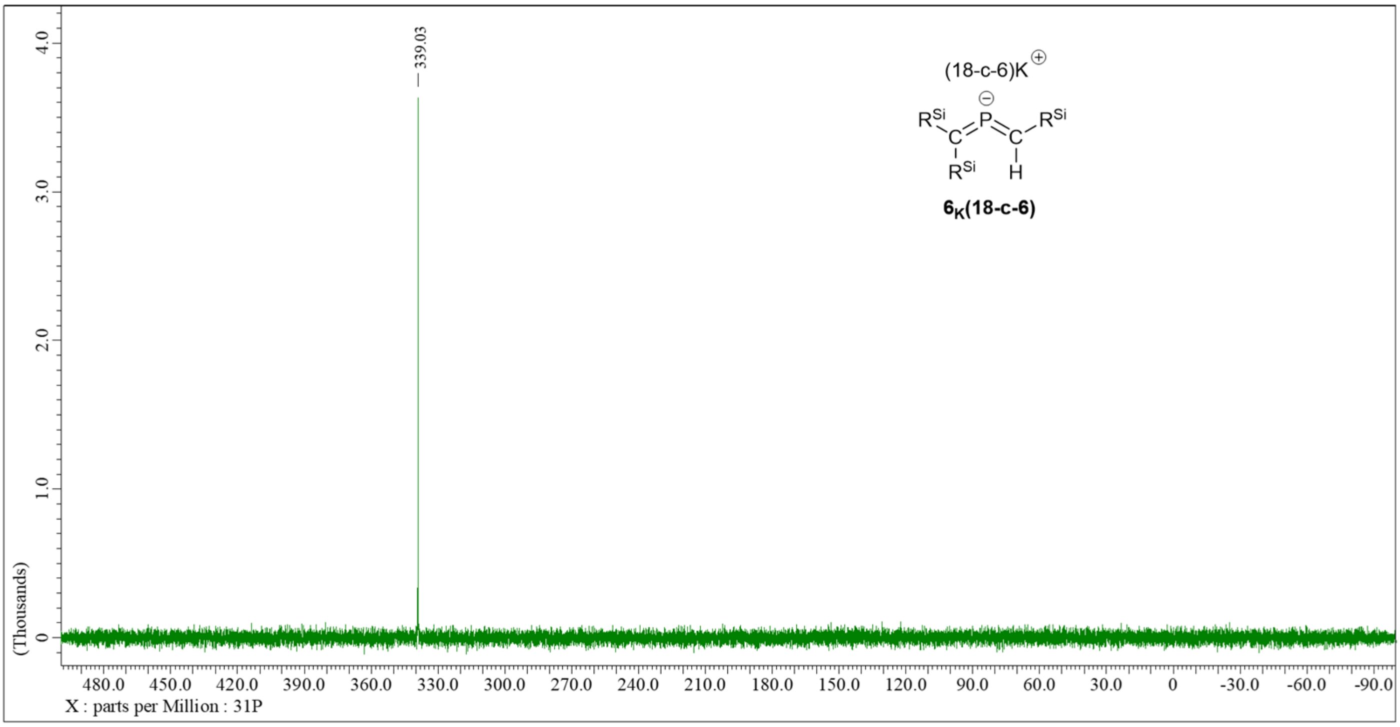

Figure S11. $^{31}P\{^{1}H\}$ NMR spectrum of **6_K·(18-c-6)** in *o*-difluorobenzene.

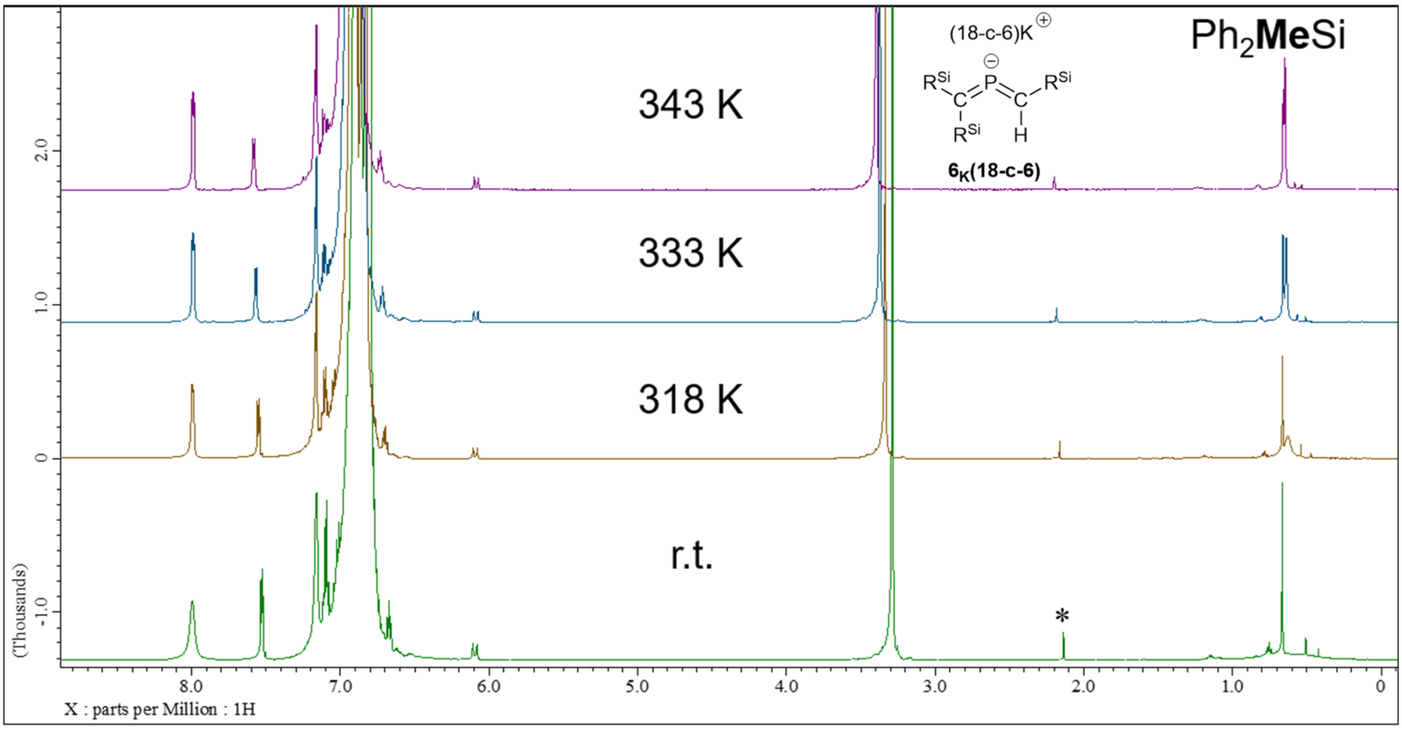

Figure S12. VT-NMR spectra for **6_K·(18-c-6)** in *o*-difluorobenzene. (*toluene)

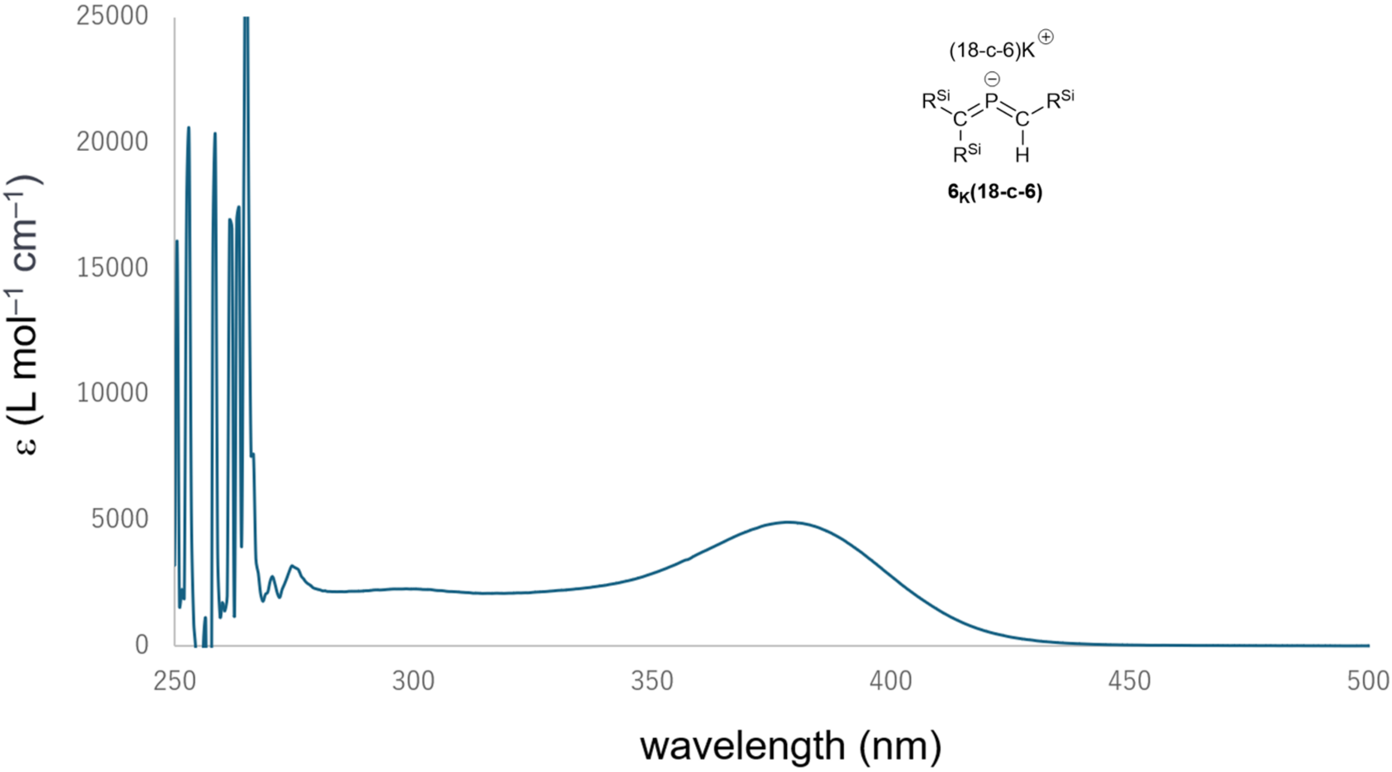

Figure S13. UV-vis spectrum of **6_K·(18-c-6)** in benzene (1.6×10^{-4} mol·L^{-1}) at room temperature.

7_K·(18-c-6): Mp. 53 ºC (dec.). ^{1}H NMR (400 MHz, C_6D_6) δ 0.94 (s, 6H), 2.96 (s, 24H), 6.41 (t, J = 7.4 Hz, 1H), 6.85 (dd, J = 7.4 Hz, J = 7.4 Hz, 2H), 7.21-7.32 (m, 15H), 8.10 (dd, J =7.8 Hz, J = 1.4 Hz, 8H); ^{13}C{^{1}H} NMR (151 MHz, C_6D_6, 333 K) δ 3.2 (d, J_{CP} = 8 Hz, CH_3), 70.1 (CH_2), 72.4 (d, J_{CP} = 74 Hz, C), 116.4 (CH), 121.2 (d, J_{CP} = 18 Hz, CH), 127.2 (CH), 127.4 (CH), 128.0 (d, J_{CP} = 44 Hz, CH), 128.4 (CH), 136.6 (CH), 145.3 (C), 150.4 (d, J_{CP} = 24 Hz, C); ^{29}Si{^{1}H} NMR (79.5 MHz, *o*-difluorobenzene) δ –15.0 (d, J_{SiP} = 21 Hz); ^{31}P NMR (162 MHz, C_6D_6) δ 306.8 (d, J_{PH} = 14.7 Hz) ; UV/vis (toluene), 474 nm (ε = 1.1×10^4).

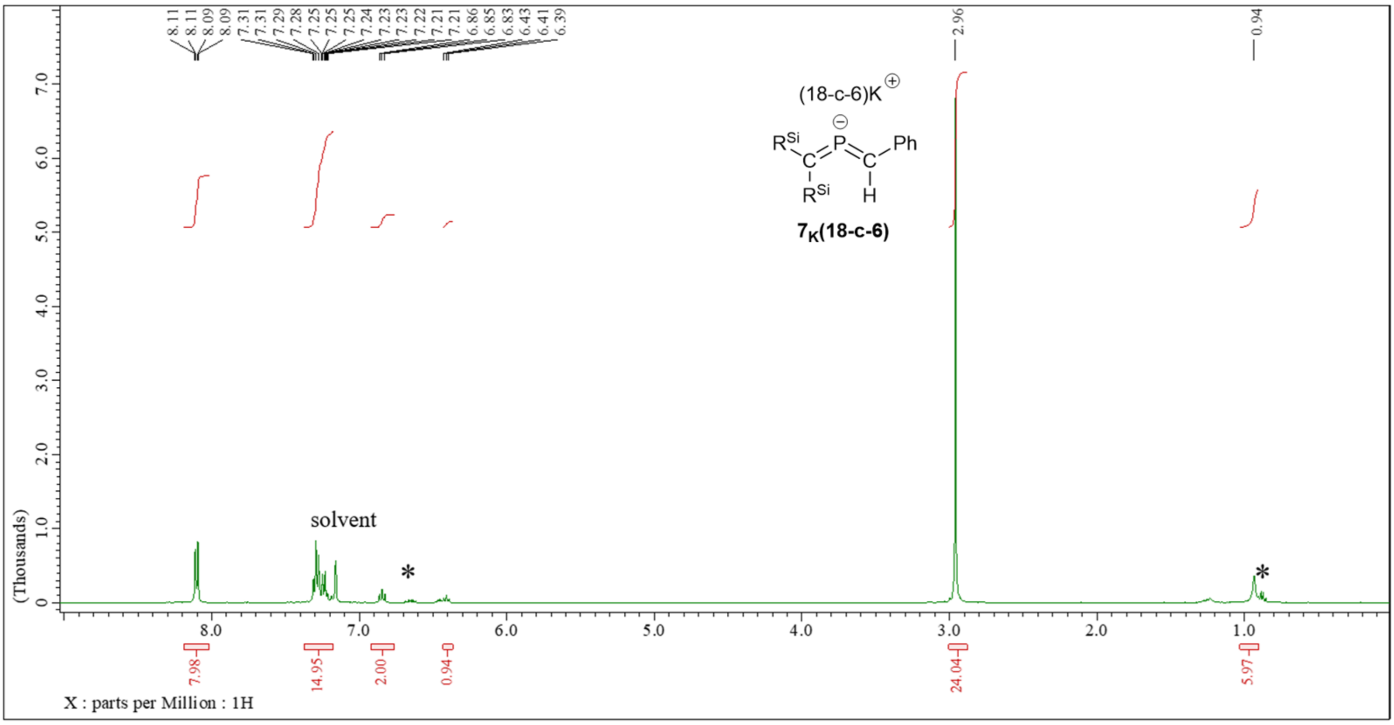

Figure S14. ^{1}H NMR spectrum of **7_K·(18-c-6)** in C_6D_6. (*hexane/*o*-defluorobenzene)

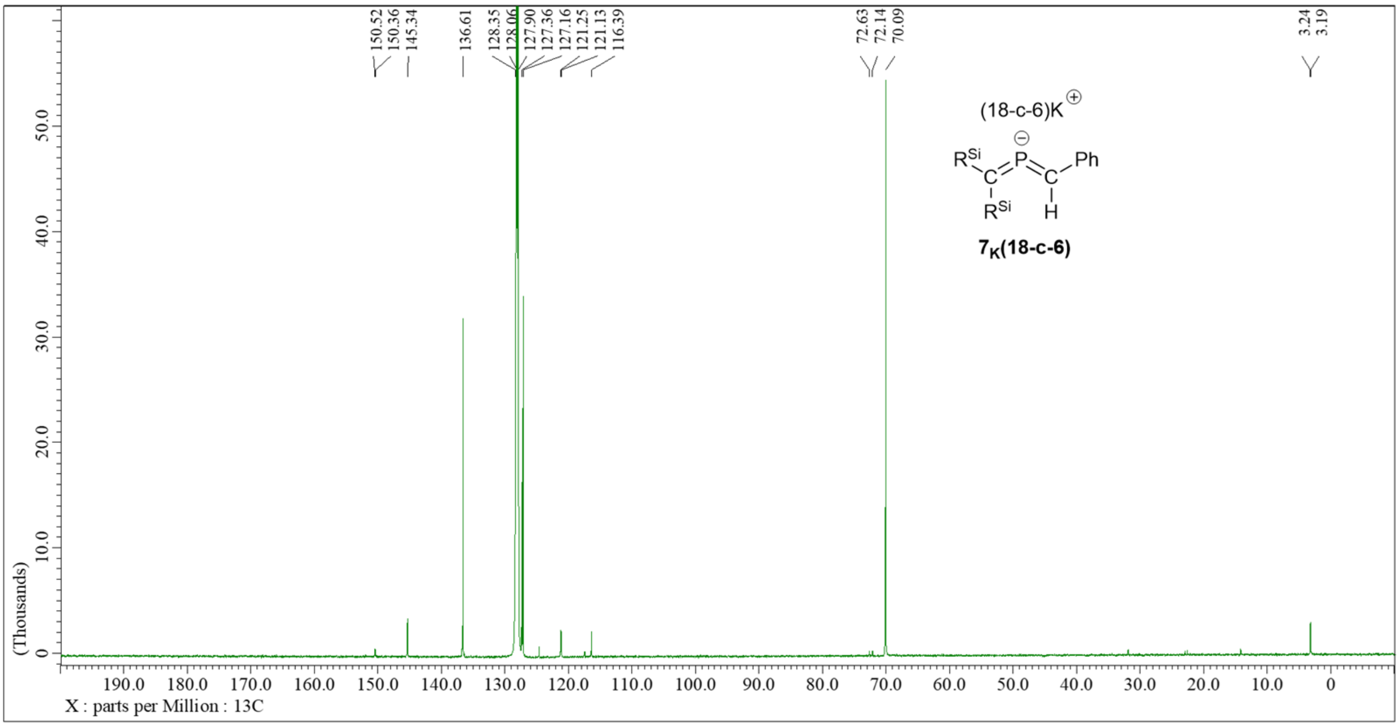

Figure S15. $^{13}C\{^1H\}$ NMR spectrum of **7_K·(18-c-6)** in C_6D_6.

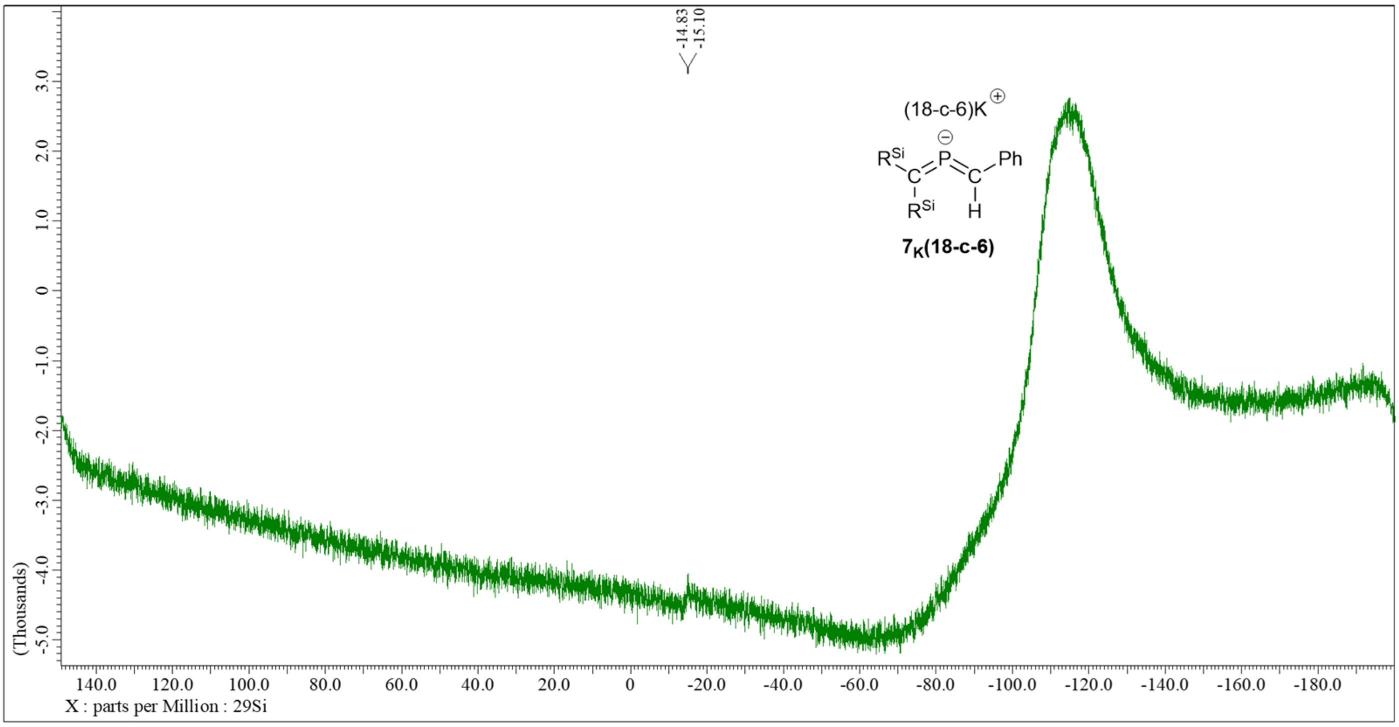

Figure S16. $^{29}Si\{^{1}H\}$ NMR spectrum of **7_K·(18-c-6)** in *o*-difluorobenzene.

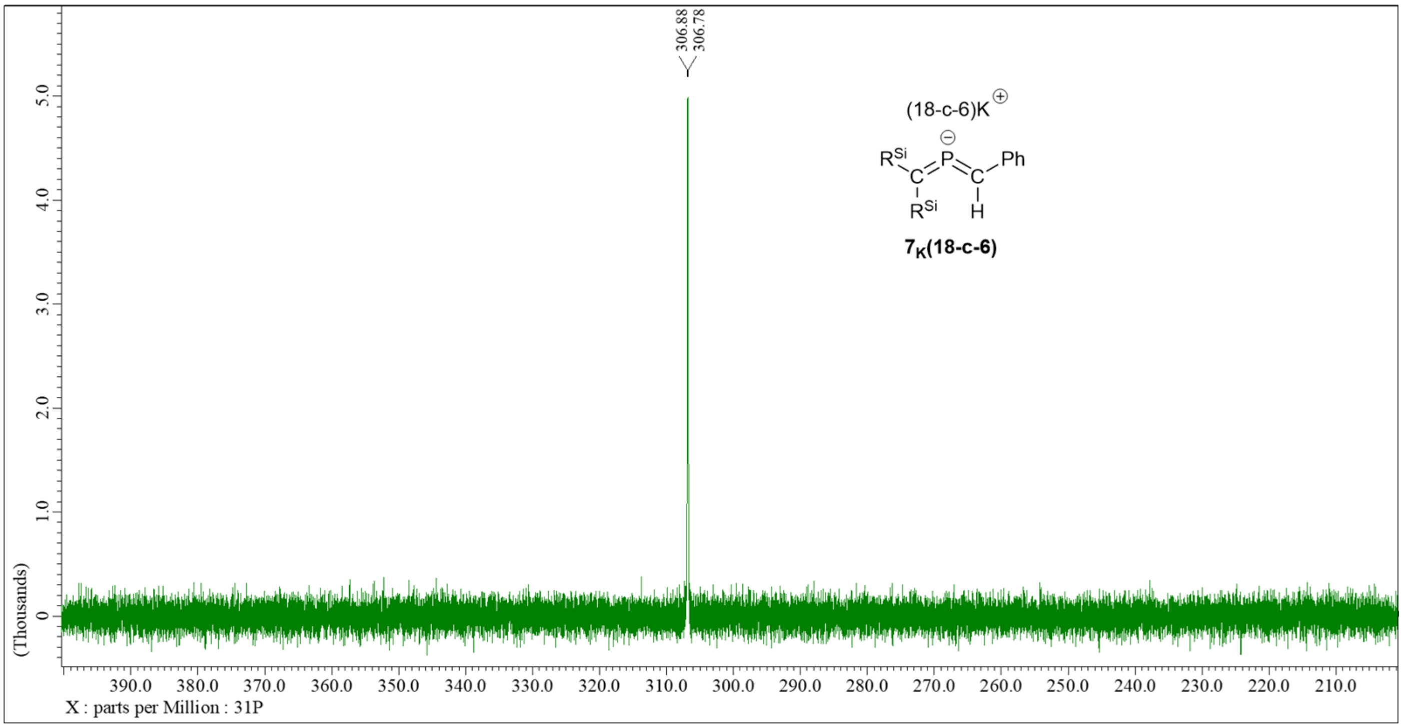

Figure S17. ^{31}P NMR spectrum of **7_K·(18-c-6)** in C_6D_6.

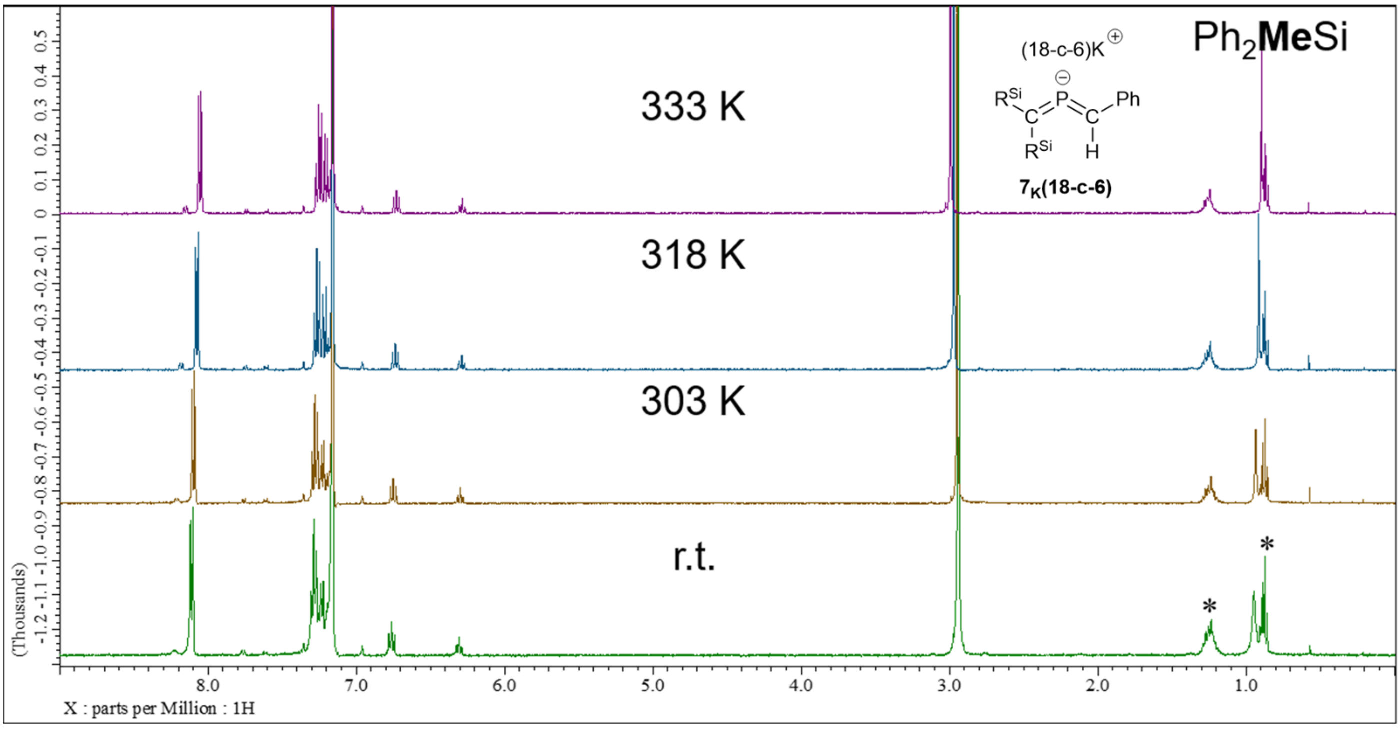

Figure S18. VT-NMR spectra for $\mathbf{7_K}$**·(18-c-6)** in C_6D_6. (*hexane)

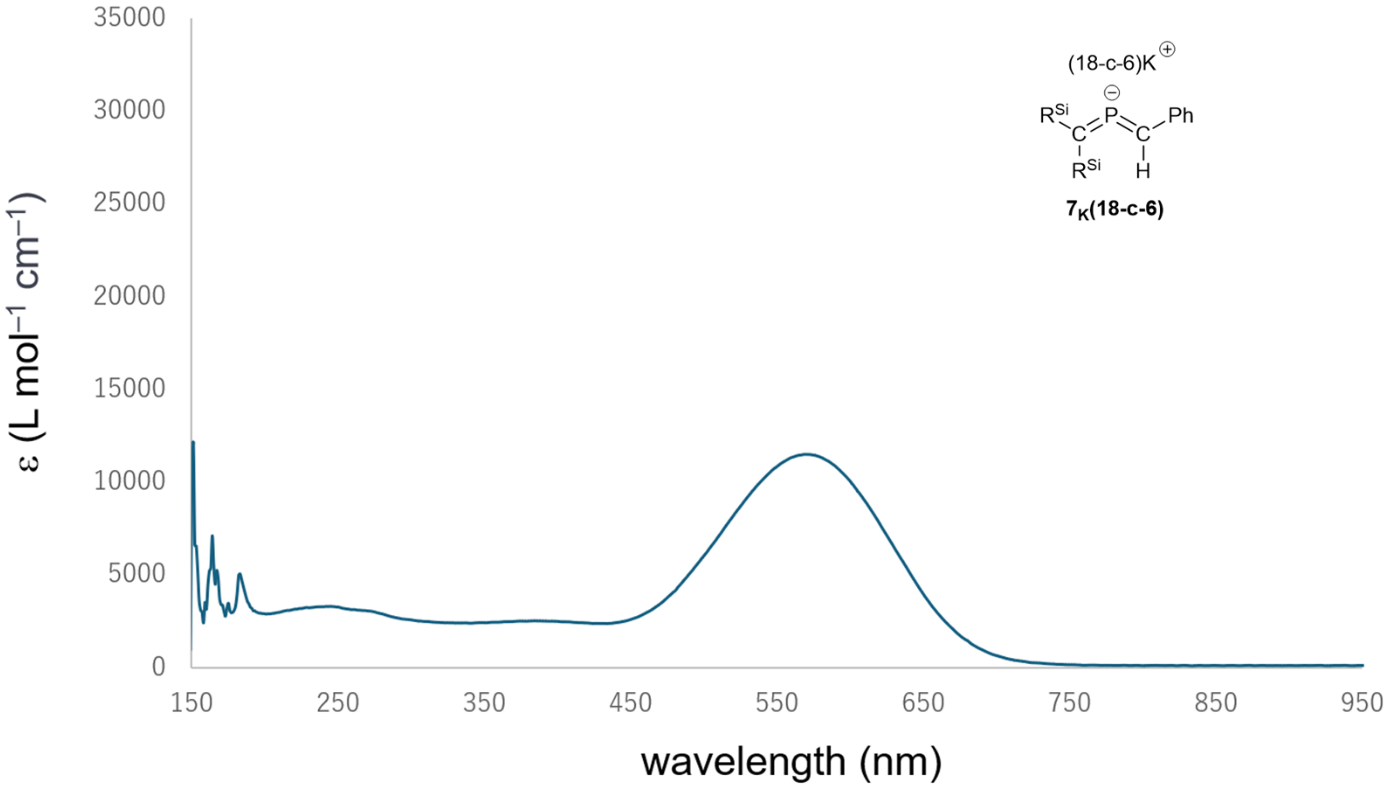

Figure S19. UV-vis spectrum of $\mathbf{7_K}$ in toluene (1.0×10^{-4} mol·L^{-1}) at room temperature.

·Synthesis of bis(methylene)-λ^5-phosphane anion $\mathbf{6_K}$·(cryptand)

A mixture of **1** (103 mg, 0.122 mmol) and 4,7,13,16,21,24-hexaoxa-1,10-diazabicyclo[8.8.8]hexaoxane (47.3 mg, 0.126 mmol) in a J Young Schlenk bottle was dissolved in toluene (1.0 mL) at 45 °C. Then, a toluene solution of KHMDS (0.4 mL, 0.5 M in toluene, 0.2 mmol) was added dropwise, and the reaction mixture was stirred at 45 °C for 6 d. Then, stirring was stopped, and the upper layer was removed. Fresh toluene was added to the flask, leading to a phase separation; the upper layer was removed, and this procedure was repeated three times. Then, all volatiles were then removed under reduced pressure, yielding a dark-red oil containing the bis(methylene)-λ^5-phosphane anion **6_K·(cryptand)** (156 mg, 96% yield judging from the ^{1}H NMR spectrum).

6_K·(cryptand): ^{1}H NMR (400 MHz, *o*-difluorobenzene) δ 0.56 (br, 6H), 0.66(3H), 2.17 (t, J = 4.4 Hz, 12H), 3.15 (t, J = 4.4 Hz, 12 H), 3.20 (s, 12H), 6.05 (d, J_{PH} = 16.8 Hz, 1H), 6.56-7.11 (m, 18H), 7.48-7.54 (m, 4H), 7.98 (br, 8H); ^{13}C{^{1}H} NMR (151 MHz, *o*-difluorobenzene, 333 K) δ –1.4 (d, J_{CP} = 14 Hz, CH_3), 3.2 (d, J_{CP} = 10 Hz, CH_3), 54.1 (CH_2), 67.7 (CH_2), 70.5 (CH_2), 74.8 (d, J_{CP} = 83 Hz, C), 108.0 (d, J_{CP} = 69 Hz, CH), 126.8 (CH), 126.8(CH), 126.9 (CH), 127.0 (CH), 135.4 (CH), 136. 5 (CH), 145.6(C), 145.8 (C); ^{29}Si{^{1}H} NMR (79.5 MHz, *o*-difluorobenzene) δ –16.6, –16.1; ^{31}P{^{1}H} NMR (162 MHz, *o*-difluorobenzene) δ 338.8 (s).

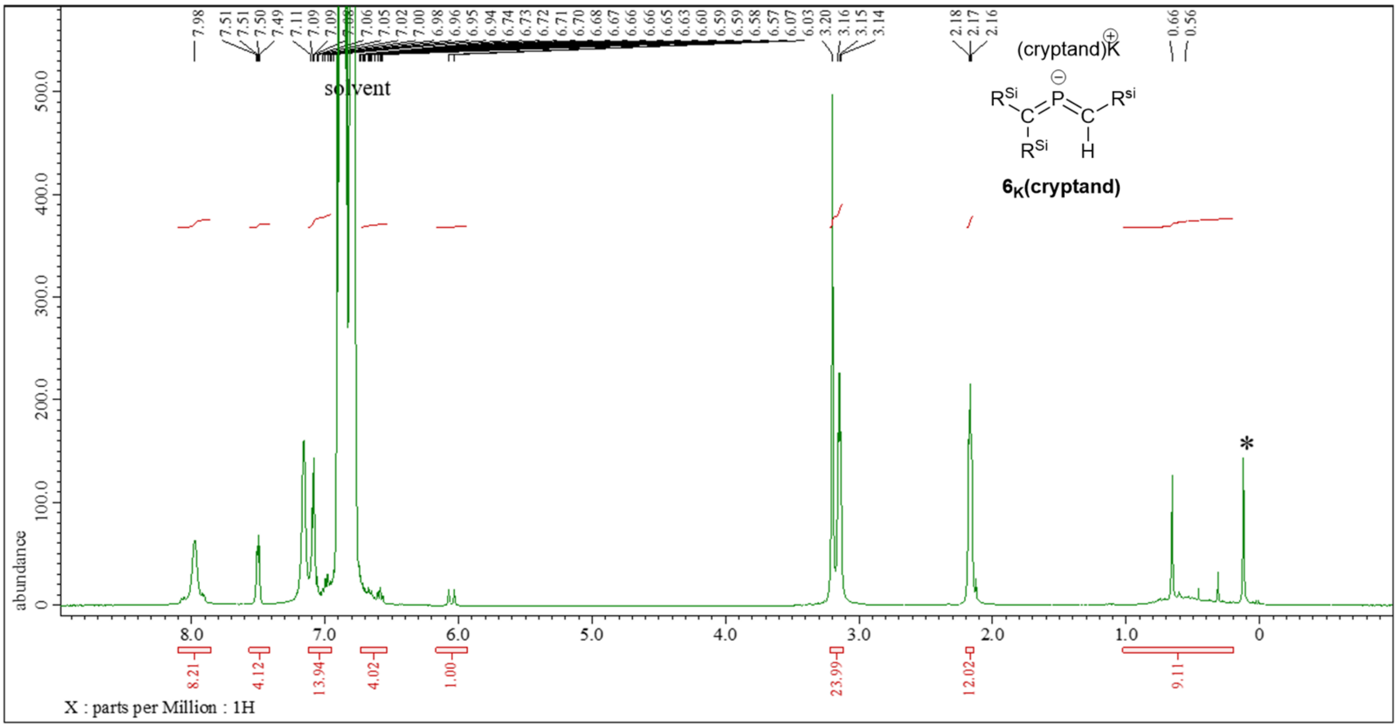

Figure S20. ^{1}H NMR spectrum of **6_K·(cryptand)** in *o*-difluorobenzene. (*KHMDS)

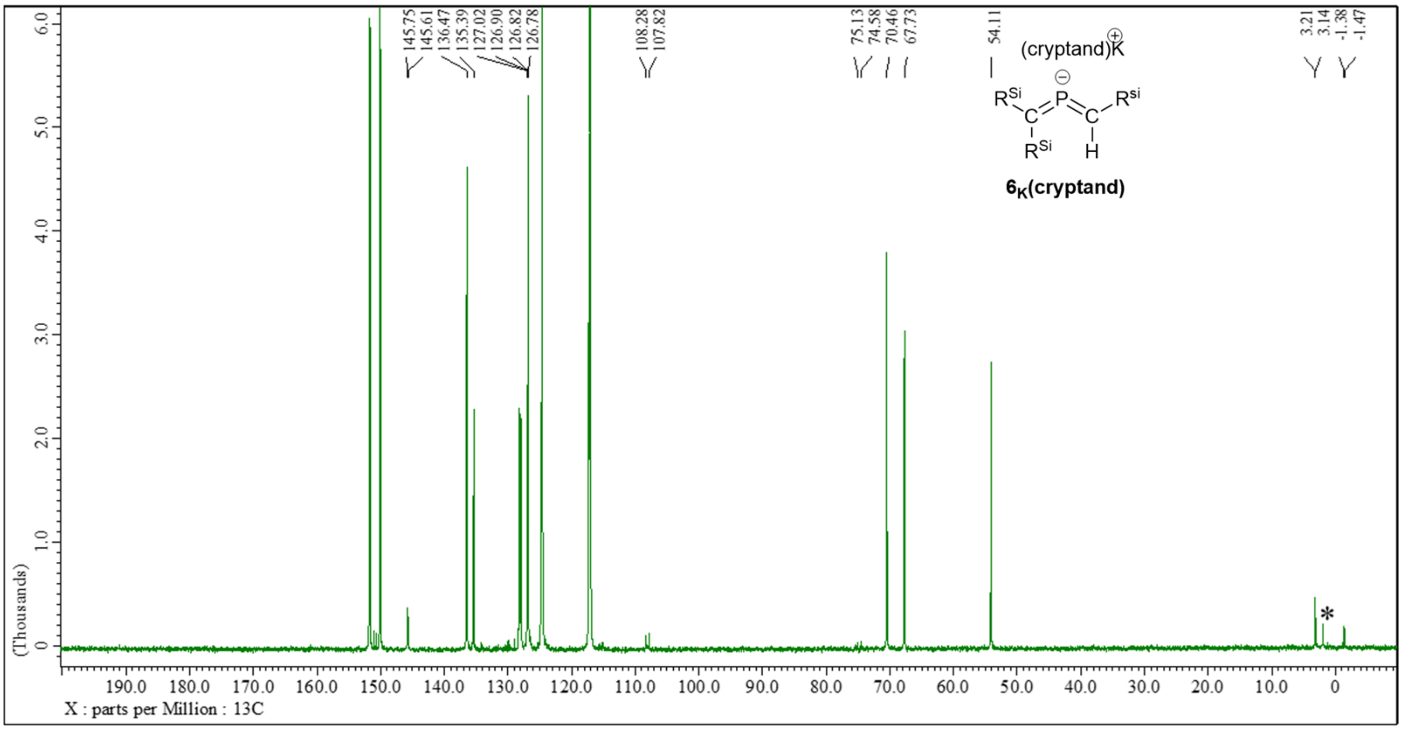

Figure S21. $^{13}C\{^{1}H\}$ NMR spectrum of $\mathbf{6_K}$**·(cryptand)** in *o*-difluorobenzene. (*KHMDS)

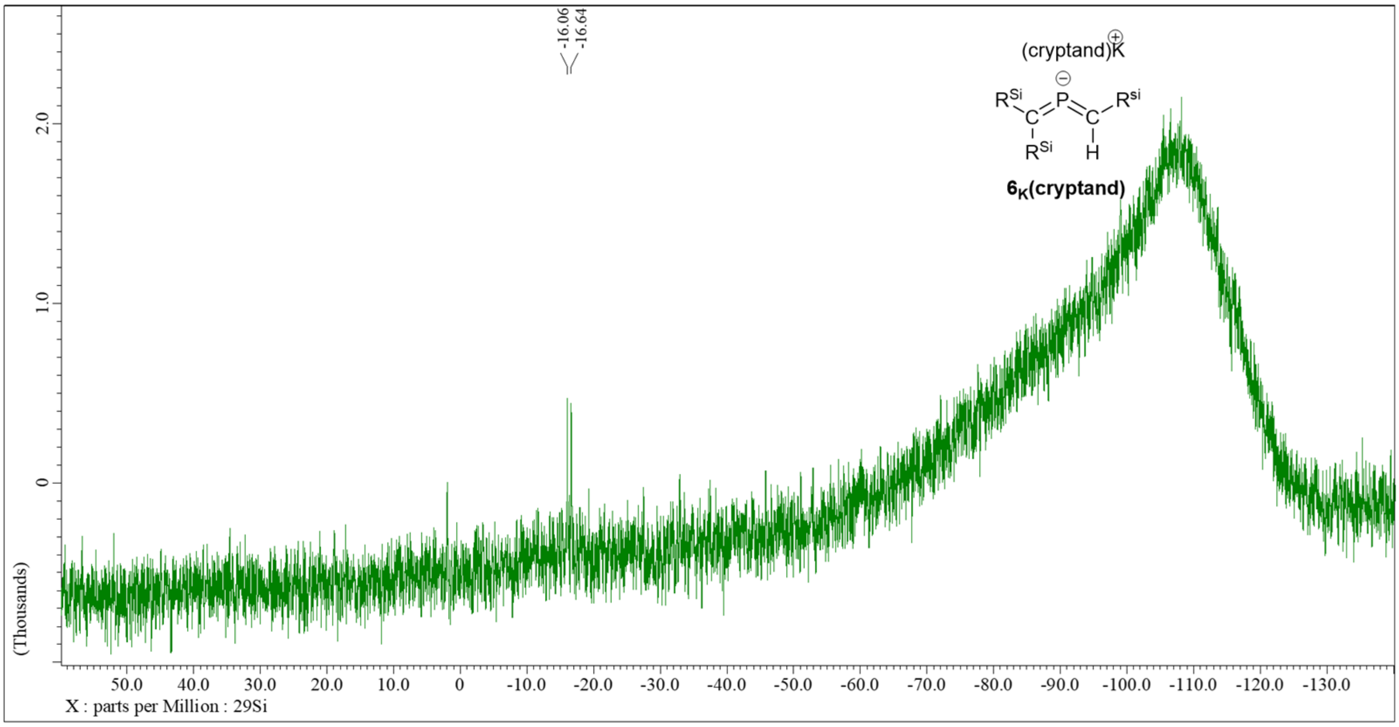

Figure S22. $^{29}Si\{^{1}H\}$ NMR spectrum of $\mathbf{6_K}$**·(cryptand)** in *o*-difluorobenzene.

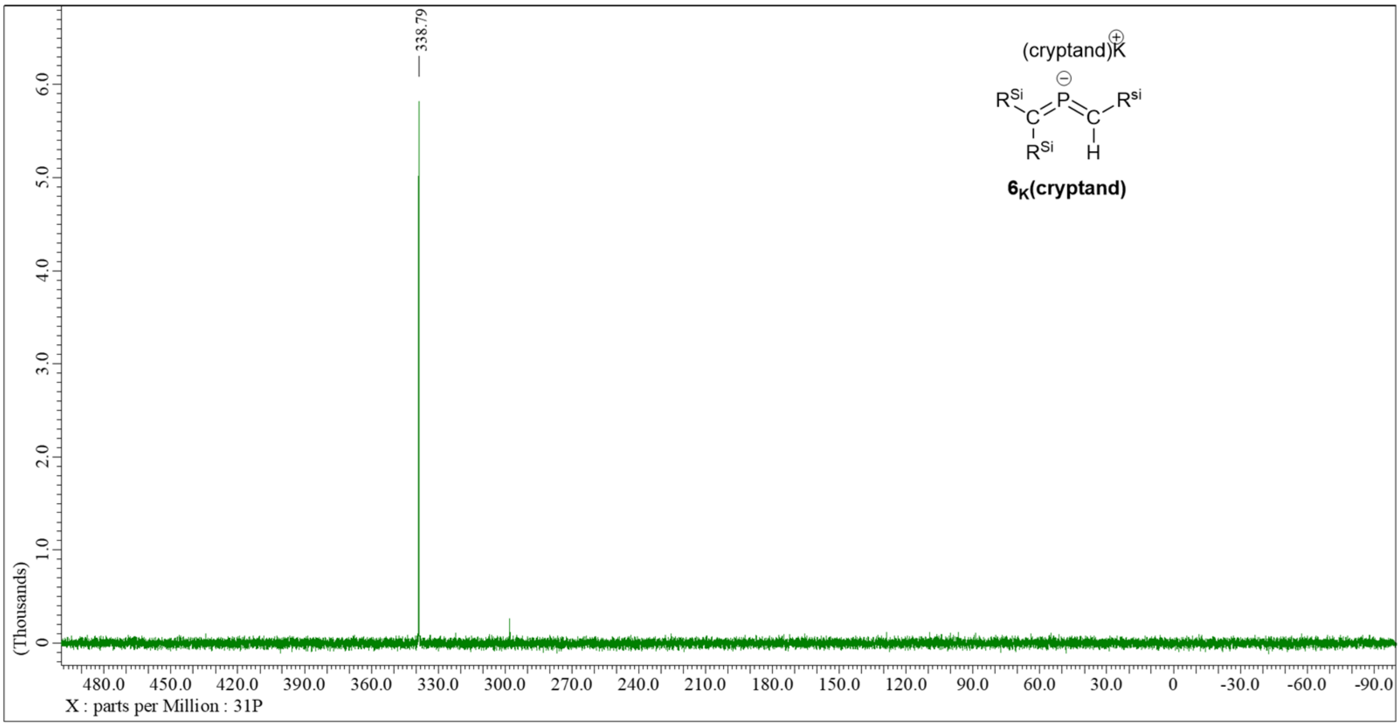

Figure S23. $^{31}P\{^1H\}$ NMR spectrum of **6_K·(cryptand)** in *o*-difluorobenzene.

- **Synthesis of bis(methylene)-λ^5-phosphane anion 6_{Li}·(12-c-4)**

To a mixture of **1** (112 mg, 0.132 mmol) and 12-crown-4 (0.08 mL, 0.5 mmol) in a J Young Schlenk bottle was added toluene (1.0 mL) at 60 ºC. Then, a toluene solution of LiHMDS (0.4 mL, 1 M in toluene, 0.4 mmol) was added dropwise, and the solution was stirred at 60 ºC for 8 d to form a pale-yellow precipitate that was isolated by decantation. The solids were washed four time with toluene, before all volatiles were removed under reduced pressure to yield bis(methylene)-λ^5-phosphane anion **6_{Li}·(12-c-4)** (16.4 mg, 0.0163 mmol, 12% yield) as a pale-yellow solid.

6_{Li}·(12-c-4): ^{1}H NMR (400 MHz, *o*-difluorobenzene) δ 0.57 (br, 6H), 0.67(3H), 3.33 (s, 32H), 6.09 (d, J_{PH} = 17.2 Hz, 1H), 6.55-7.12 (m, 18H), 7.50-7.56 (m, 4H), 7.99 (br, 8H); $^{13}C\{^1H\}$ NMR (151 MHz, *o*-difluorobenzene, 333 K) δ –1.5 (d, J_{CP} = 14 Hz, CH_3), 3.2 (d, J_{CP} = 9 Hz, CH_3), 67.3 (CH_2), 75.1 (d, J_{CP} = 82 Hz, C), 107.9 (d, J_{CP} = 69 Hz, CH), 126.8 (CH), 126.9(CH), 127.0 (CH), 127.1 (CH), 135.4 (CH), 136.5 (CH), 145.6 (C), 145.7 (C); $^{29}Si\{^1H\}$ NMR (79.5 MHz, *o*-difluorobenzene) δ –16.6, –16.0; $^{31}P\{^1H\}$ NMR (162 MHz, C_6D_6) δ 338.8 (s); HRMS(DART-negative), *m/z*: Found: 647.2149 ([**6_{Li}**]$^-$), calcd. For $C_{41}H_{40}PSi_3$ ([**6_{Li}**]$^-$): 647.2175

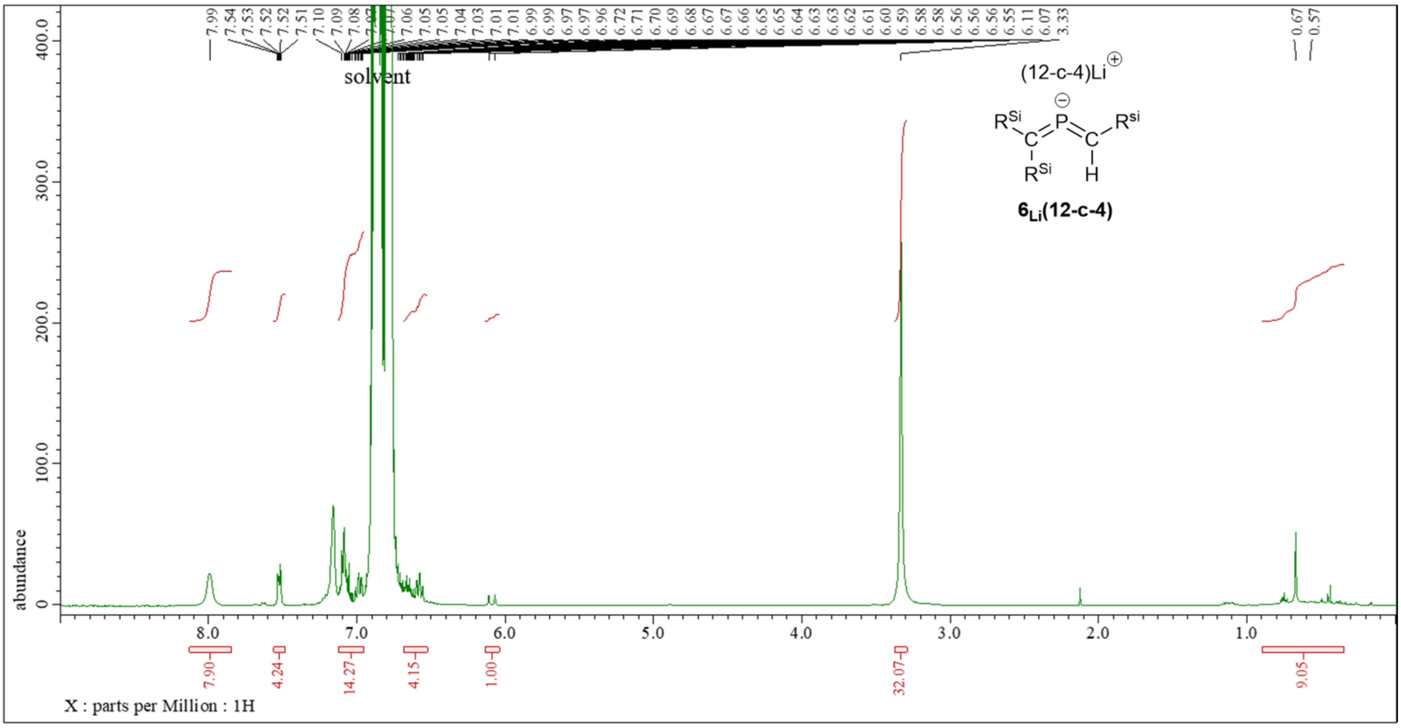

Figure S24. ^{1}H NMR spectrum of $\mathbf{6_{Li}{\cdot}(12\text{-}c\text{-}4)}$ in *o*-difluorobenzene.

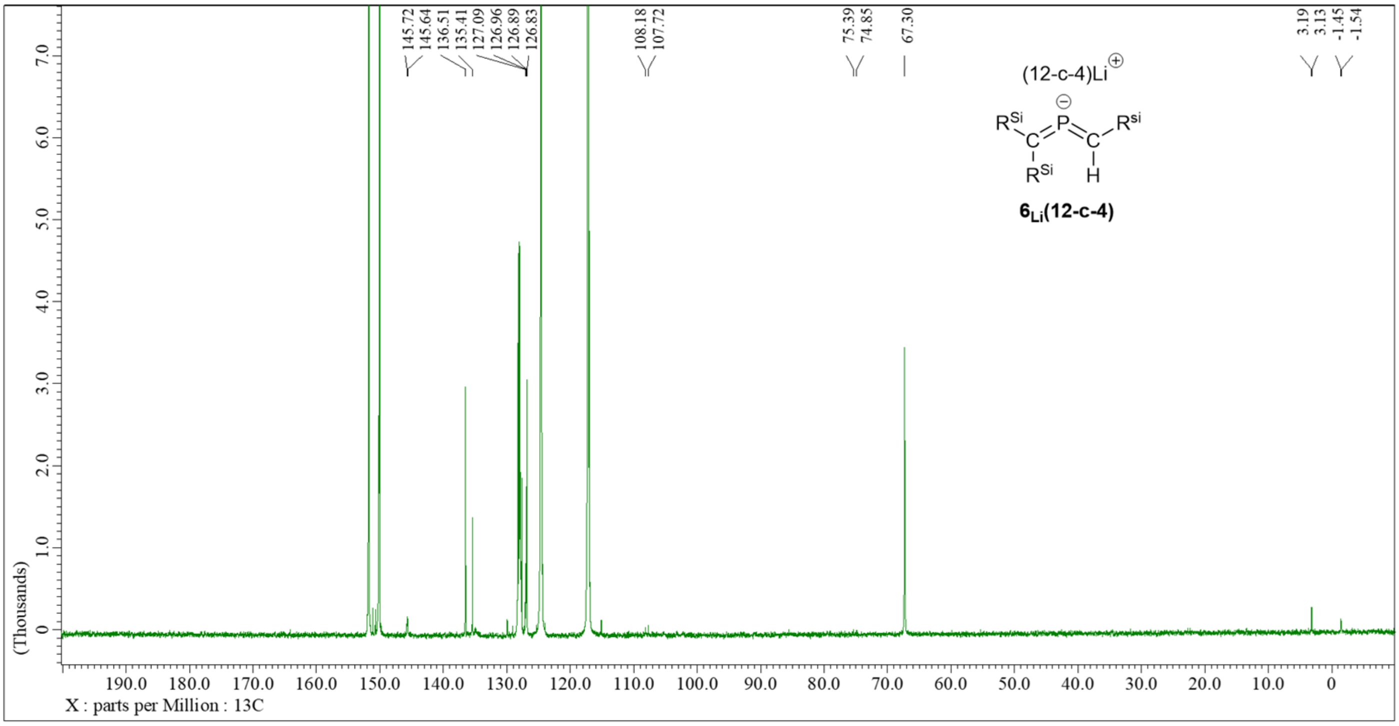

Figure S25. $^{13}C\{^{1}H\}$ NMR spectrum of $\mathbf{6_{Li}{\cdot}(12\text{-}c\text{-}4)}$ in *o*-difluorobenzene.

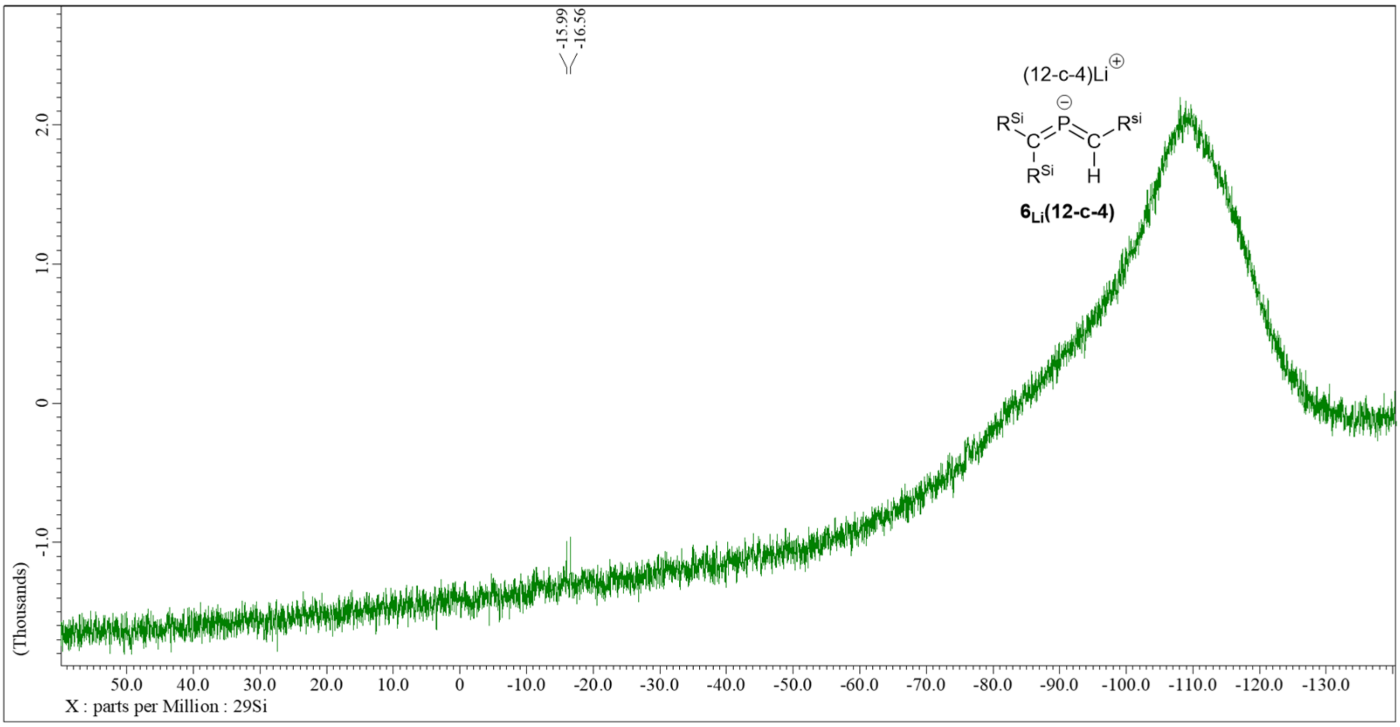

Figure S26. ^{29}Si NMR spectrum of $\mathbf{6_{Li}}$**·(12-c-4)** in *o*-difluorobenzene.

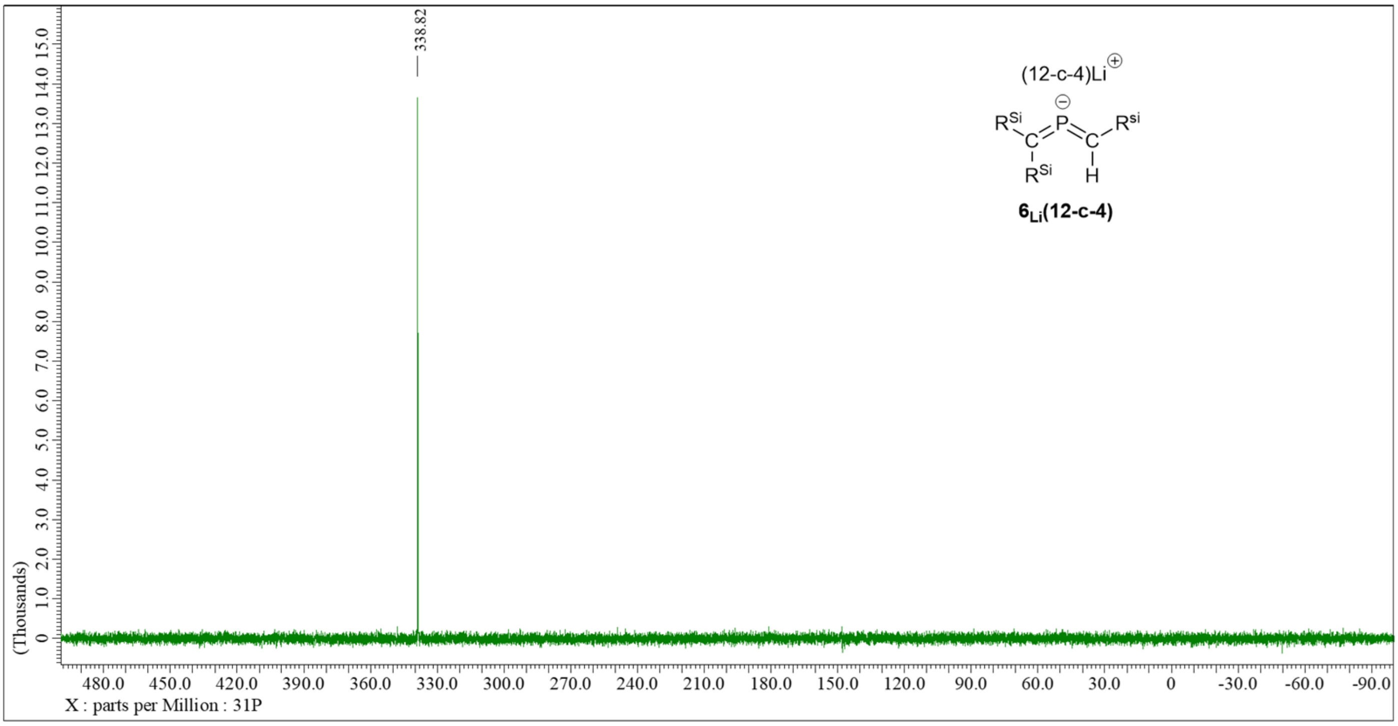

Figure S27. ^{31}P{^{1}H} NMR spectrum of $\mathbf{6_{Li}}$**·(12-c-4)** in *o*-difluorobenzene.

·Synthesis of bis(methylene)-λ^5-phosphane anion $\mathbf{6_{Na}}$·(18-c-6)

A mixture of **1** (106 mg, 0.125 mmol) and 18-crown-6 (67.3 mg, 0.255 mmol) was dissolved in toluene (1.0 mL) in a J Young Schlenk bottle at 60 °C. Then, a toluene solution of NaHMDS (0.4 mL, 0.6 M in toluene, 0.2 mmol) was added dropwise to the flask, and the reaction mixture was stirred at 60 °C for 3 d. Subsequently, stirring was stopped, and the upper layer was removed. Fresh toluene was added to the flask, resulting in a phase separation; the upper layer was removed, and this procedure was repeated five times, before all volatiles were then removed under reduced pressure to yield bis(methylene)-λ^5-

phosphane anion **6_{Na}·(18-c-6)** (90.4 mg, 77%) as a dark-red oil.

6_{Na}·(18-c-6): ^{1}H NMR (400 MHz, *o*-difluorobenzene) δ 0.58 (br, 6H), 0.65(3H), 3.28 (s, 24H), 6.06 (d, J_{PH} = 16.8 Hz, 1H), 6.48-7.11 (m, 18H), 7.48-7.52 (m, 4H), 7.98 (br, 8H); $^{13}C\{^{1}H\}$ NMR (151 MHz, *o*-difluorobenzene, 333 K) δ –1.5 (d, J_{CP} = 14 Hz, CH_3), 3.2 (d, J_{CP} = 9 Hz, CH_3), 68.9 (CH_2), 75.0 (d, J_{CP} = 84 Hz, C), 108.0 (d, J_{CP} = 69 Hz, CH), 126.8 (CH), 126.9(CH), 127.0 (CH), 127.1 (CH), 135.4 (CH), 136.5 (CH), 145.6 (C), 145.7 (C) ; $^{29}Si\{^{1}H\}$ NMR (79.5 MHz, *o*-difluorobenzene) δ –16.6, –16.0; $^{31}P\{^{1}H\}$ NMR (162 MHz, C_6D_6) δ 338.7 (s); HRMS(DART-negative), *m/z*: Found: 647.2149 ([6_{Na}]$^{-}$), calcd. For $C_{41}H_{40}PSi_3$ ([6_{Na}]$^{-}$): 647.2175.

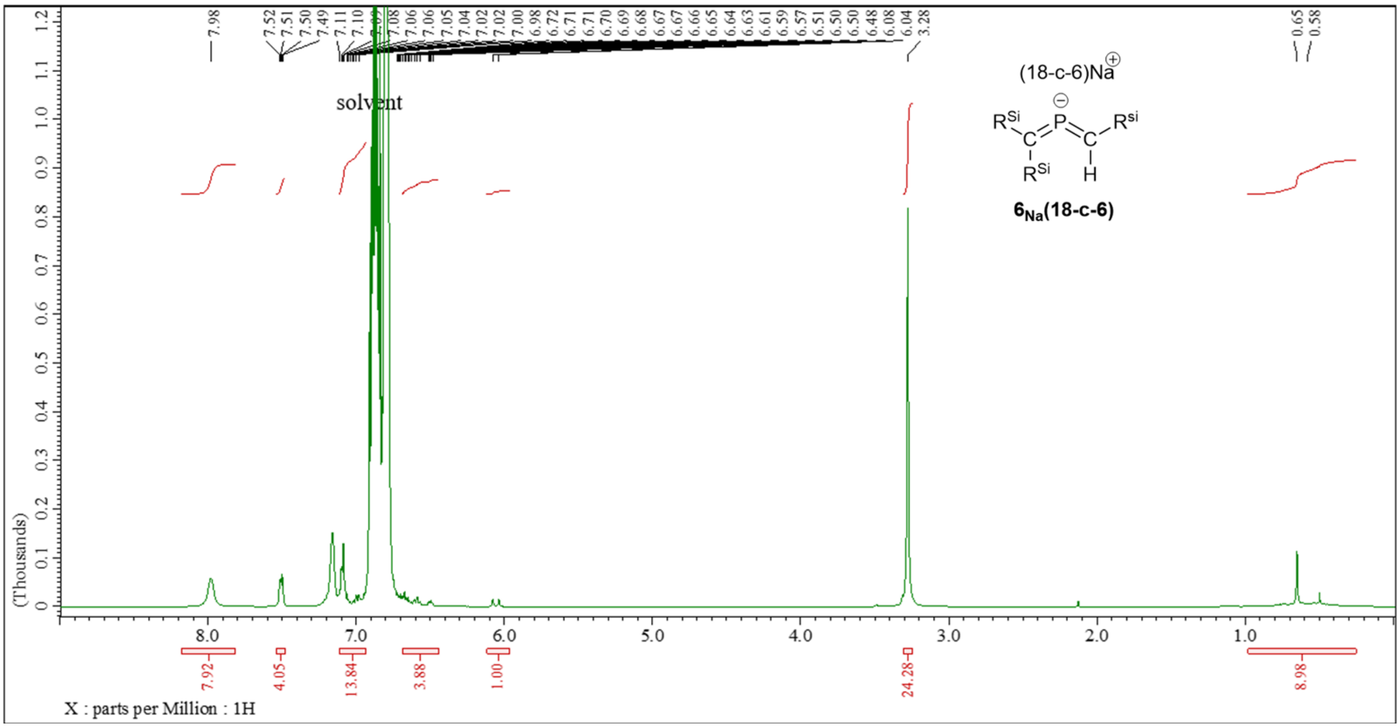

Figure S28. ^{1}H NMR spectrum of **6_{Na}·(18-c-6)** in *o*-difluorobenzene.

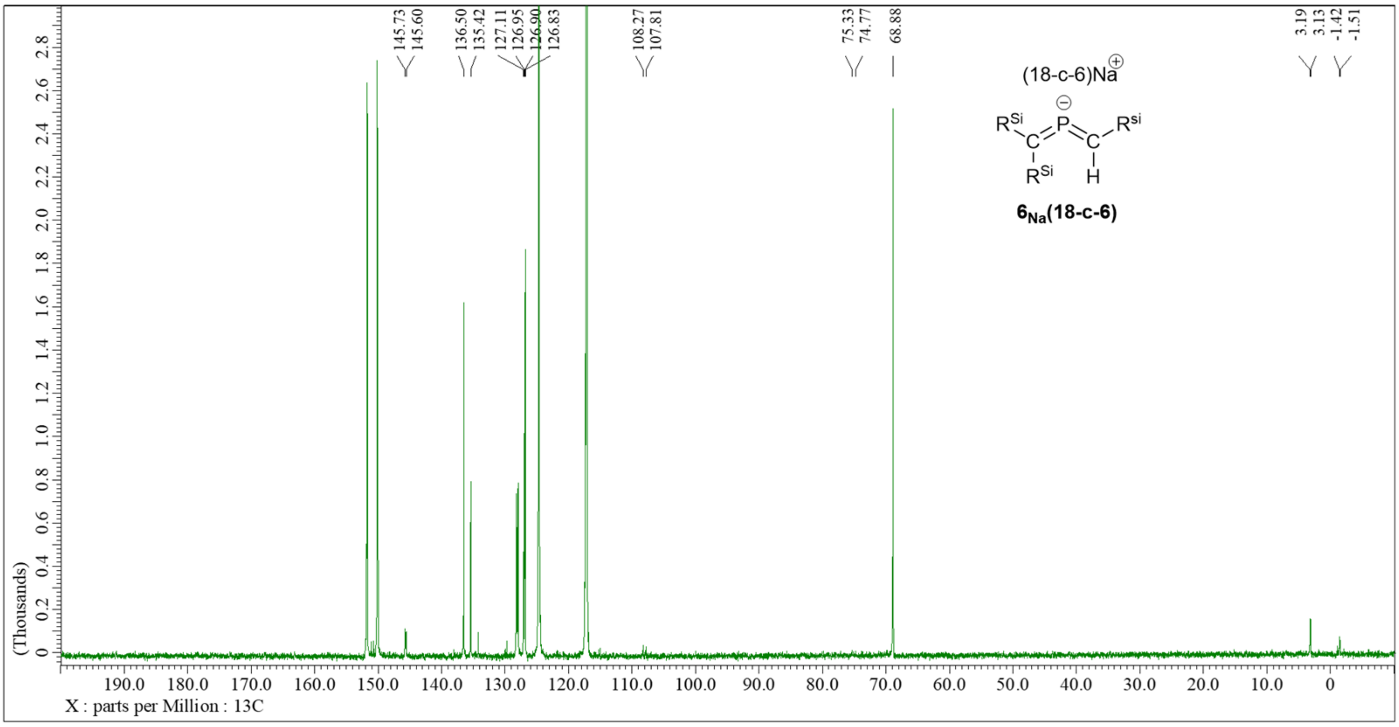

Figure S29. $^{13}C\{^{1}H\}$ NMR spectrum of **6_{Na}·(18-c-6)** in *o*-difluorobenzene.

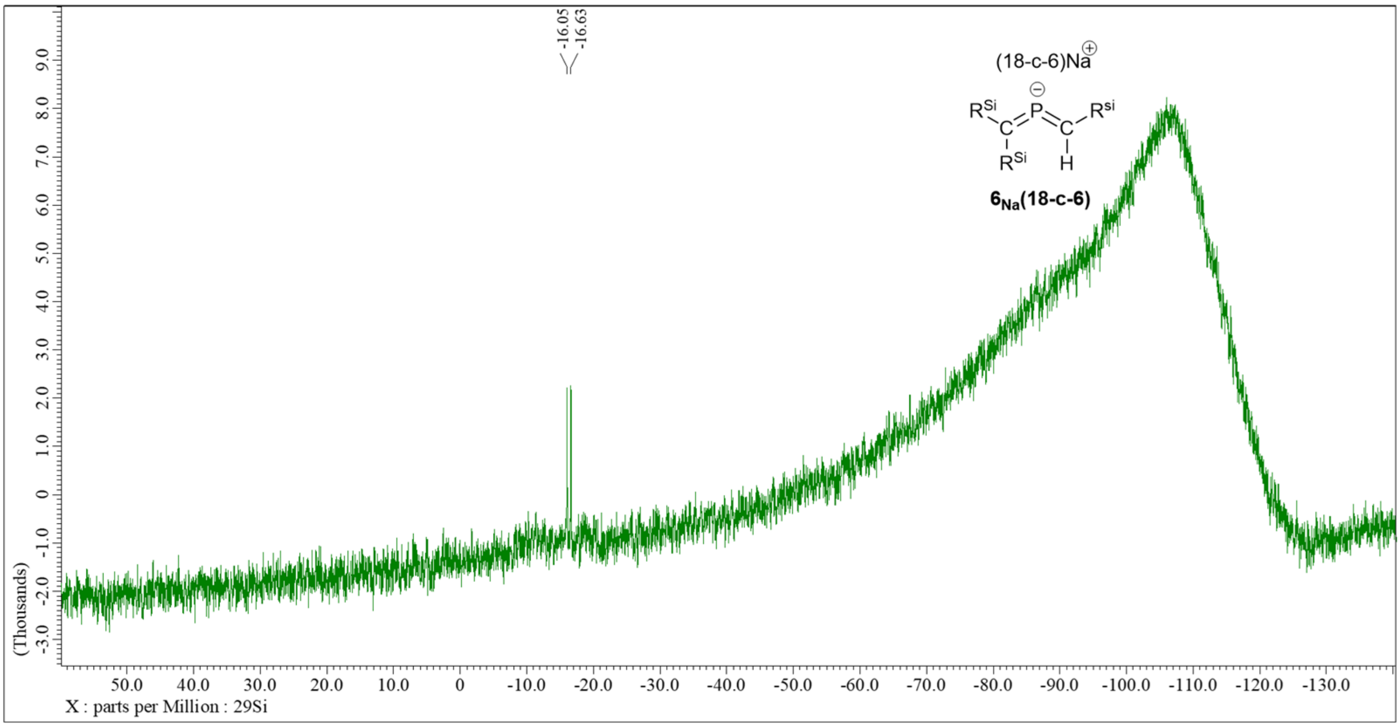

Figure S30. ^{29}Si{^{1}H} NMR spectrum of **6_{Na}·(18-c-6)** in *o*-difluorobenzene.

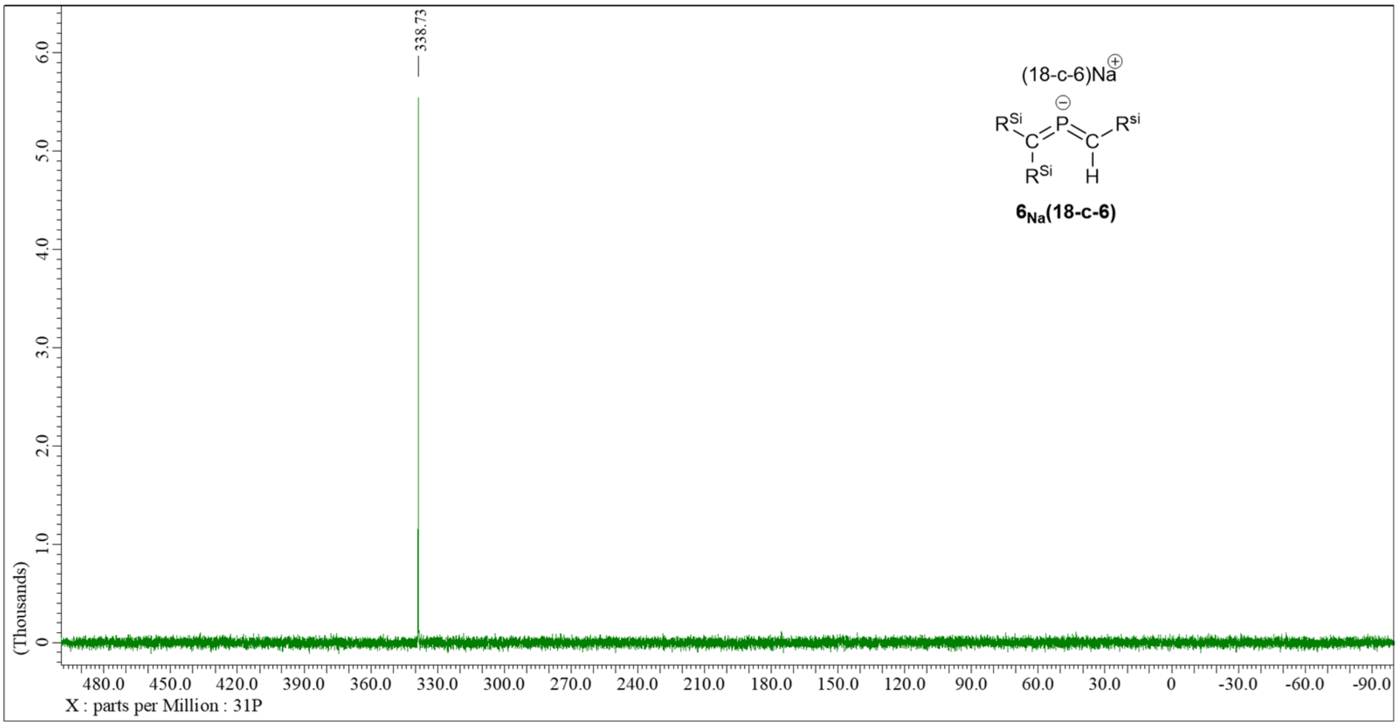

Figure S31. ^{31}P NMR spectrum of **6_{Na}·(18-c-6)** in *o*-difluorobenzene.

·**Reaction of 1 with *t*-BuOK**

To a mixture of compound **1** (109 mg, 0.129 mmol), 18-crown-6 (68.4 mg, 0.259 mmol), and *t*-BuOK (21.5 mg, 0.192 mmol) in a J Young Schlenk bottle was added toluene (1.0 mL) at 45 °C. The solution was stirred at same temperature for 43.5 h to form pale yellow precipitates, which was isolated by the decantation. The solids were washed twice with toluene, and all volatiles were removed under reduced pressure, yielding a pale yellow solid (78.2 mg).

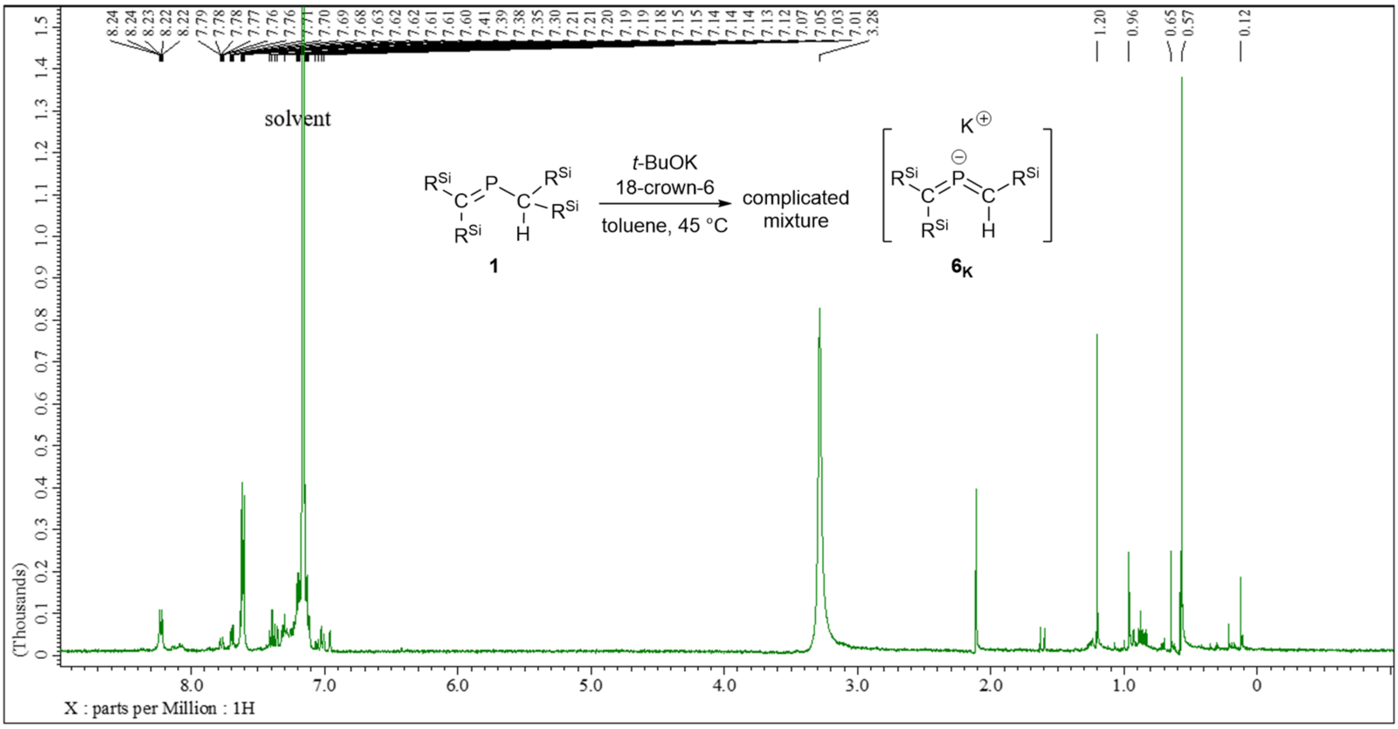

Figure S32. ^{1}H NMR spectrum of the crude product in C_6D_6.

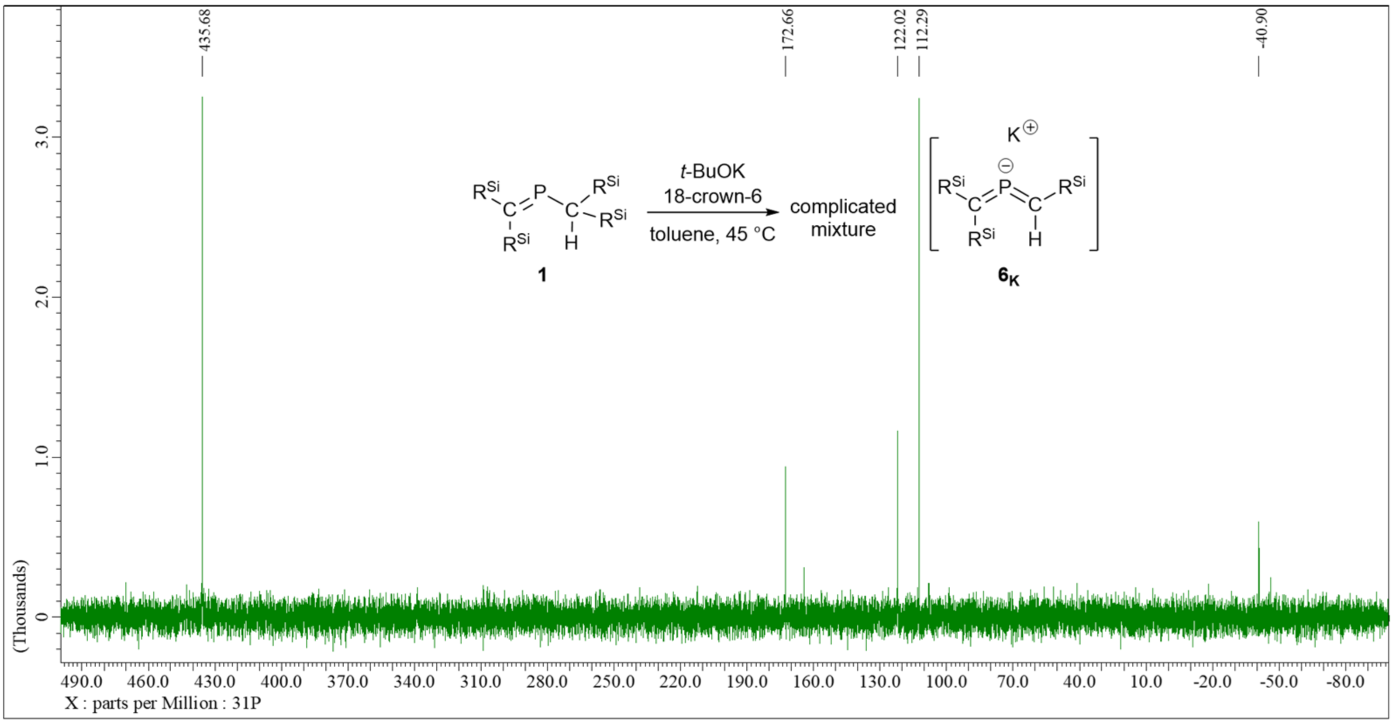

Figure S33. ^{31}P NMR spectrum of the toluene supernatant.

·Reaction of 6_K·(18-c-6) with *t*-Bu$_3$PHBF$_4$

A benzene (1 mL) solution of **6K·(18-c-6)** (54.8 mg, 0.0576 mmol) in a glass bottle was treated with *t*-Bu$_3$PHBF$_4$ (17.8 mg, 0.0614 mmol) at room temperature. After 3 h of stirring at this temperature, the mixture was filtered through a pad of Celite, before all volatiles were removed under reduced pressure to yield proton-adduct **9** as a pale-yellow oil (39.7 mg).

9: ^{1}H NMR (400 MHz, C_6D_6) δ 0.43 (s, 3H), 0.50 (s, 3H), 0.62 (s, 3H), 2.32 (d, J = 2.4 Hz, 2H), 7.06-7.22 (m, 18H), 7.31(ddd, J = 6.0 Hz, J = 1.6 Hz, J = 1.6 Hz, 4H), 7.41 (ddd, J = 6.0 Hz, J = 2.0 Hz, J = 2.0 Hz, 4H), 7.58 (ddd, J = 5.2 Hz, J = 2.0 Hz, J = 2.0 Hz, 4H); ^{13}C{^{1}H} NMR (101 MHz, C_6D_6) δ –2.8 (d, J_{CP} = 4 Hz, CH$_3$), –0.1 (d, J_{CP} = 12 Hz, CH$_3$), 1.23 (CH$_3$), 33.3 (d, J_{CP} = 73 Hz, CH$_2$), 127.9 (CH), 128.1 (CH), 128.2 (CH), 128.6 (CH), 129.3 (CH), 129.6 (CH), 135.2 (CH), 135.5 (CH), 135.8 (CH), 136.6 (C), 138.1 (C), 138.2 (C), 187.8 (d, J_{CP} = 89 Hz, C) ; ^{29}Si{^{1}H} NMR (79.5 MHz, C_6D_6) δ –43.3, –18.2 (d, J_{SiP} = 11 Hz), –9.6 (d, J_{SiP} = 9 Hz); ^{31}P{^{1}H} NMR (162 MHz, C_6D_6) δ 427.0 (s); HRMS(DART-positive), *m*/*z*: Found: 649.2321 ([**9**+H]$^+$), calcd. For $C_{41}H_{41}PSi_3$ ([**9**+H]$^+$): 649.2332.

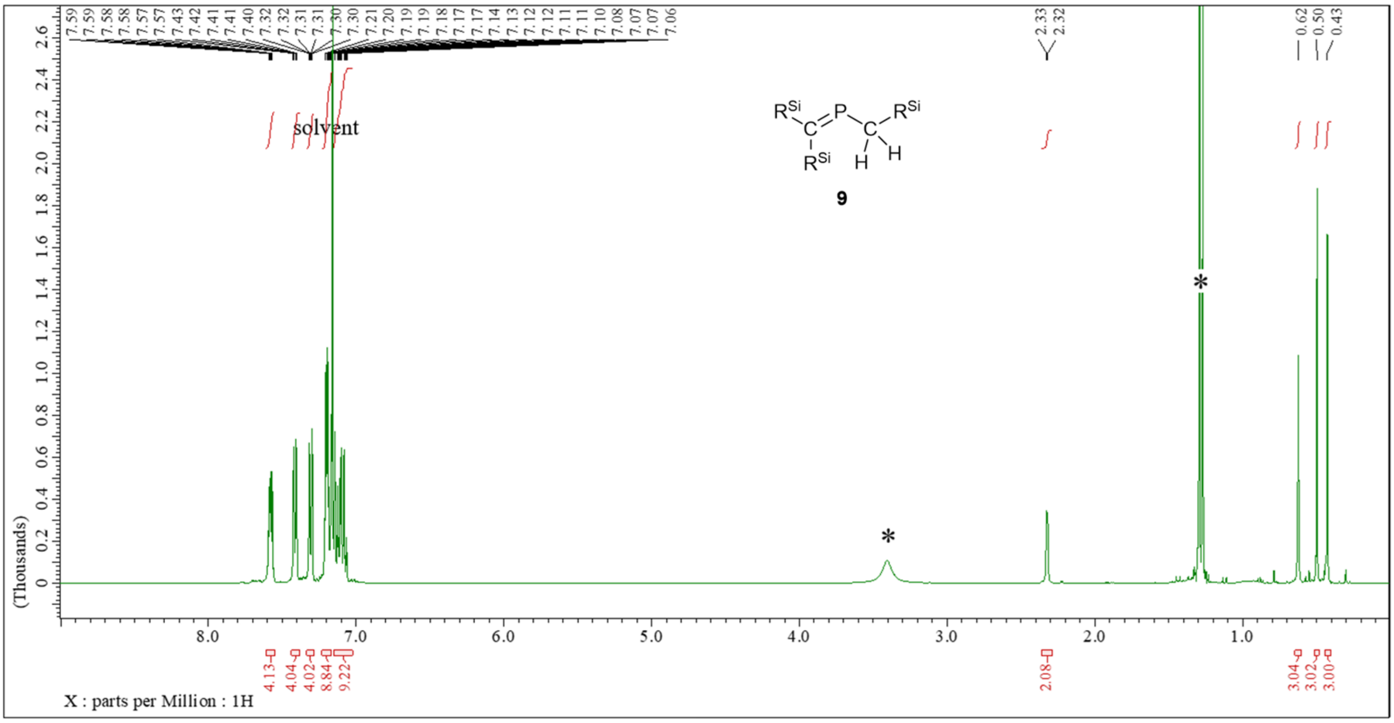

Figure S34. ^{1}H NMR spectrum of **9** in C_6D_6. (*K$^+$(18-c-6)·BF_4^-/(*t*-Bu)$_3$P)

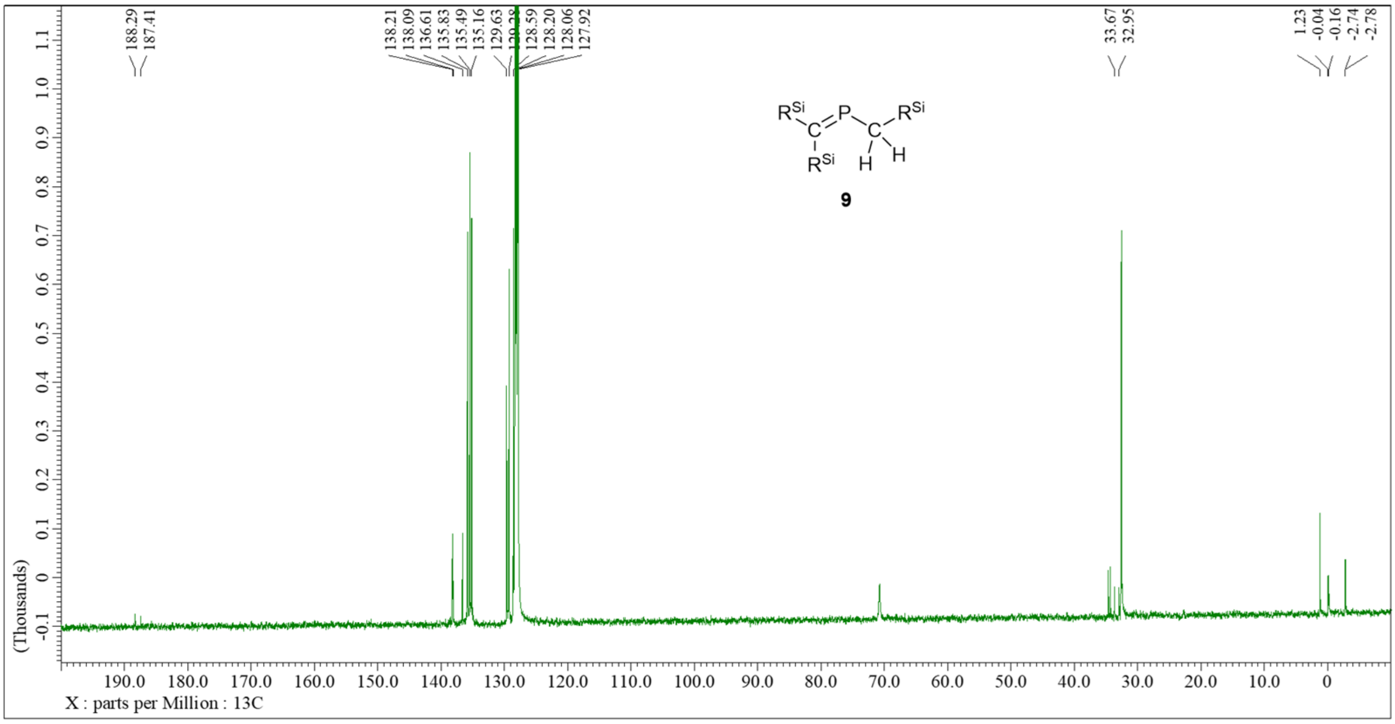

Figure S35. $^{13}C\{^{1}H\}$ NMR spectrum of **9** in C_6D_6.

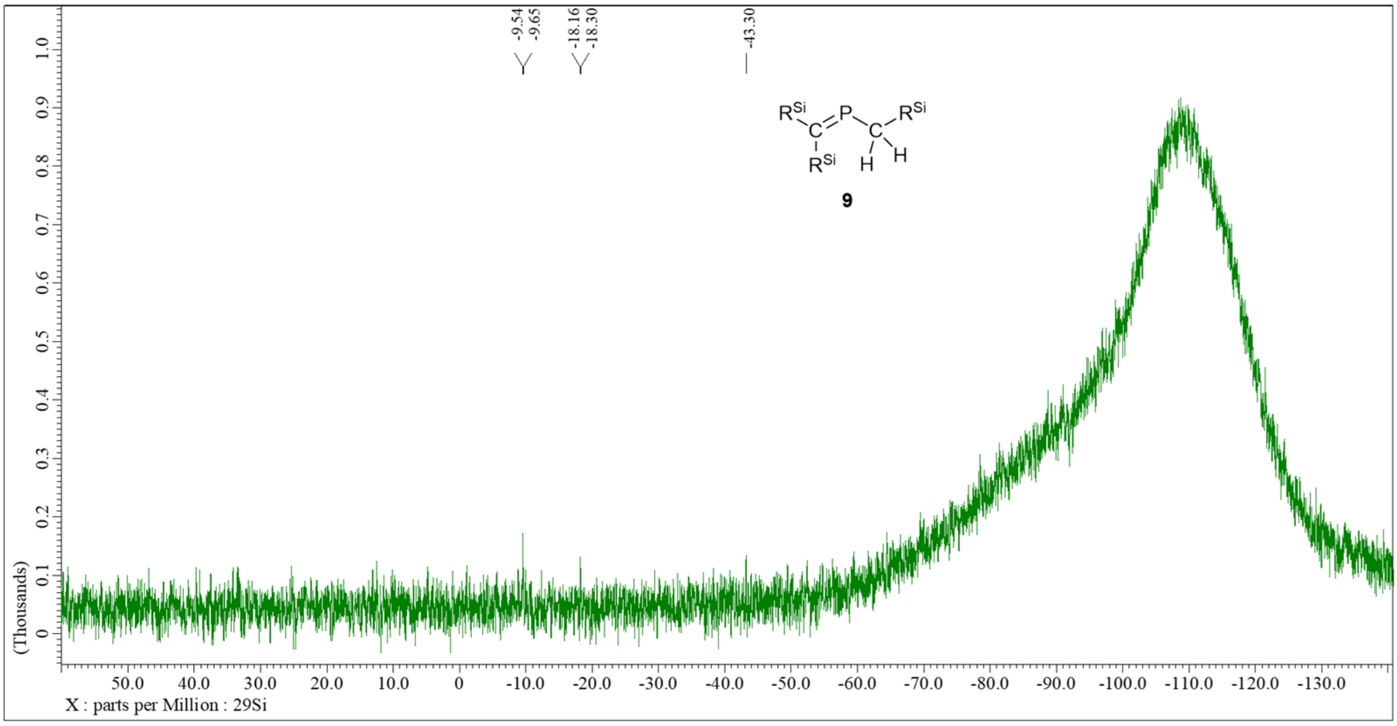

Figure S36. $^{29}Si\{^{1}H\}$ NMR spectrum of **9** in C_6D_6.

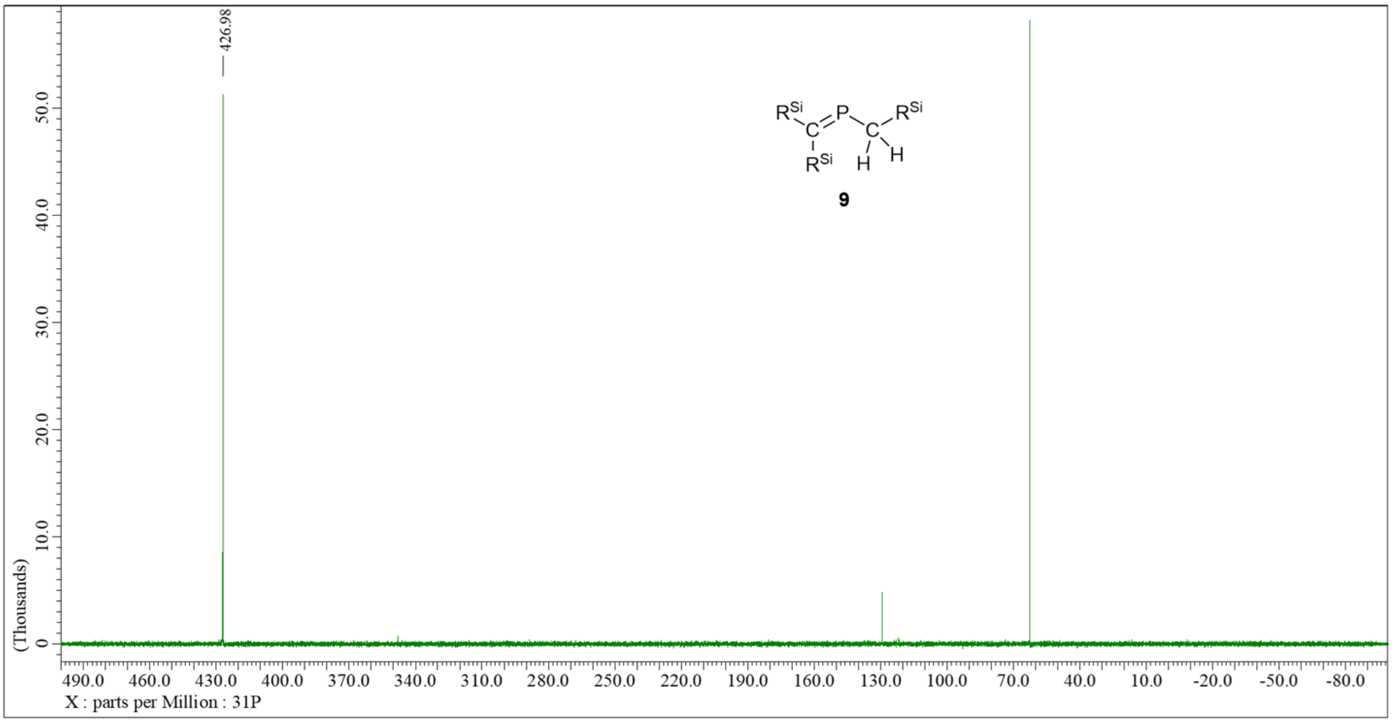

Figure S37. $^{31}P\{^{1}H\}$ NMR spectrum of **9** in C_6D_6.

·Reaction of $\mathbf{7_K}$·(18-c-6) with *t*-Bu$_3$PHBF$_4$

A benzene (0.5 mL) solution of $\mathbf{7_K}$**·(18-c-6)** (22.1 mg, 0.026 mmol) in a glass bottle was treated with *t*-Bu$_3$PHBF$_4$ (7.7 mg, 0.026 mmol) at room temperature. After stirring at this temperature for 17 h, the mixture was filtered through a pad of Celite, before all volatiles were removed under reduced pressure to yield proton-adduct **10** as a pale-yellow oil (13.2 mg).

10: ^{1}H NMR (400 MHz, C_6D_6) δ 0.58 (s, 3H), 0.70 (s, 3H), 3.35 (d, J = 3.6 Hz, 2H), 6.73 (d, J = 6.8 Hz, 2H), 6.92 (t, J = 7.3 Hz, 1H), 7.00 (dddd, J = 7.3 Hz, J = 7.3 Hz, J = 1.5 Hz, J = 1.5 Hz, 2H), 7.12-7.18 (m, 12H), 7.52 (ddd, J = 5.6 Hz, J = 2.0 Hz, J = 2.0 Hz) 4H), 7.59-7.62 (m, 4H); $^{13}C\{^{1}H\}$ NMR (101 MHz, C_6D_6) δ –0.4 (d, J_{CP} = 13 Hz, CH_3), 1.5 (CH_3), 48.5 (d, J_{CP} = 58 Hz, CH_2), 126.1 (CH), 128.0 (CH), 128.2 (CH),128.6(CH), 129.0 (CH), 129.3 (d, J_{CP} = 5 Hz, CH), 129.5 (d, J_{CP} = 7 Hz, CH), 135.5 (CH), 135.8 (CH), 137.4 (d, J_{CP} = 7 Hz, C), 137.7 (C), 138.1 (d, J_{CP} = 9 Hz, C), 191.6 (d, J_{CP} = 90 Hz, C) ; $^{29}Si\{^{1}H\}$ NMR (119 MHz, C_6D_6) δ –17.5 (d, J_{SiP} = 10 Hz), –9.8 (d, J_{SiP} = 33 Hz) ; $^{31}P\{^{1}H\}$ NMR (162 MHz, C_6D_6) δ 427.7 (s) ; HRMS(DART-positive), *m/z*: Found: 546.1926 ([**10**+H_2O]$^+$), calcd. For $C_{34}H_{35}OPSi_2$ ([**10**+H_2O]$^+$): 546.1964.

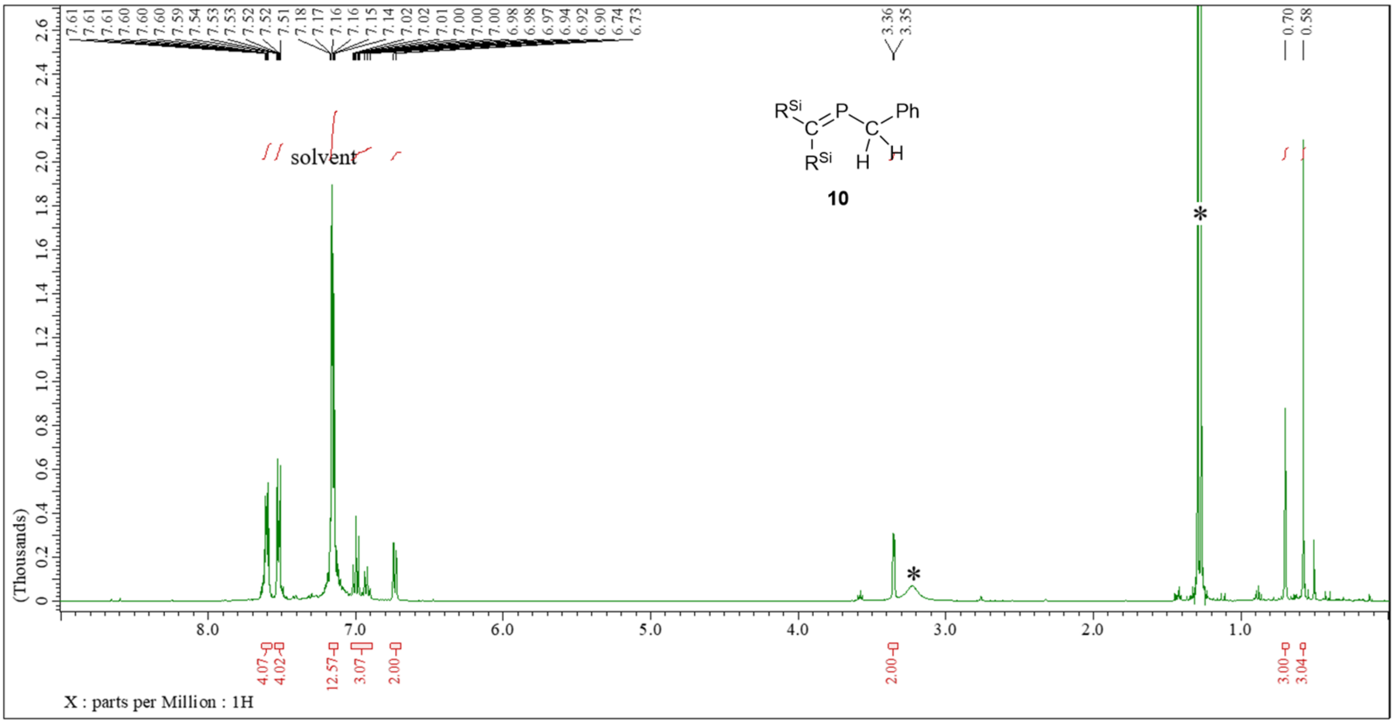

Figure S38. ^{1}H NMR spectrum of **10** in C_6D_6. (*K^+(18-c-6)·BF_4^-/(*t*-Bu)$_3$P)

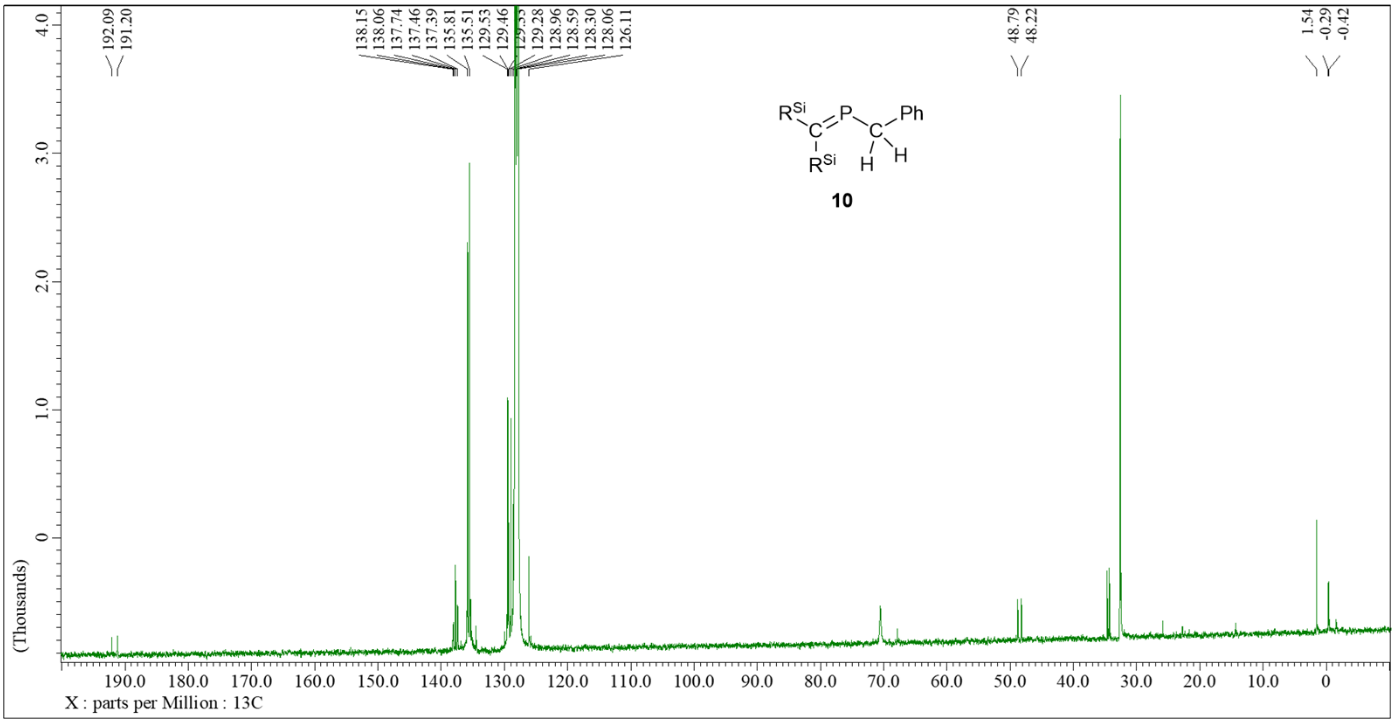

Figure S39. ^{13}C NMR spectrum of **10** in C_6D_6.

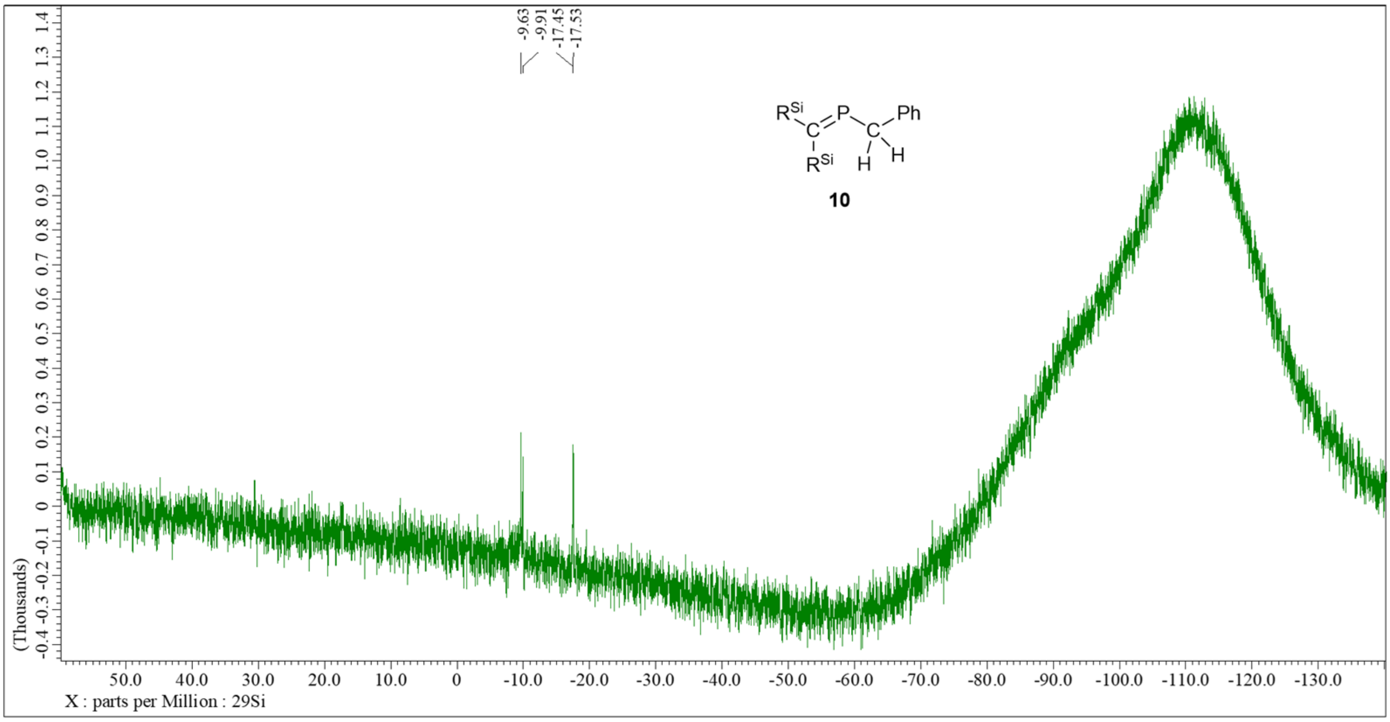

Figure S40. $^{29}Si\{^{1}H\}$ NMR spectrum of **10** in C_6D_6

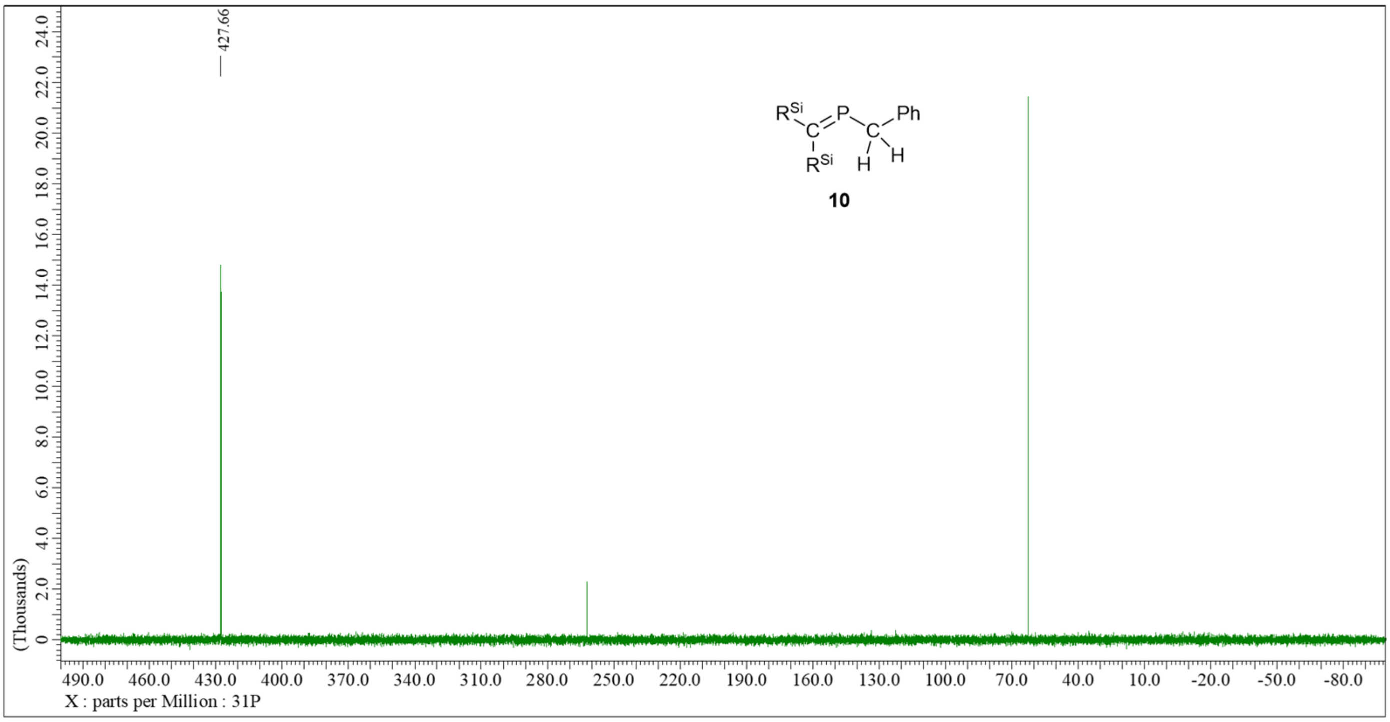

Figure S41. ^{31}P NMR spectrum of **10** in C_6D_6.

X-Ray Crystallographic Analysis

Single crystals of **1**, **6_K·(18-c-6)**, and **7_K·(18-c-6)** were obtained after recrystallization from toluene/hexane (**1**), benzene (**6_K·(18-c-6)**), or toluene/pentane (**6_K·(18-c-6)**) at room temperature. Intensity data of **1** and **6_K·(18-c-6)** were collected on a Bruker APEX-II system using Mo-Kα radiation (λ = 0.71073 Å), while those of **6_K·(18-c-6)** were collected on the BL02B1 beamline of SPring-8 (proposal numbers: 2023A1539, 2023A1771, 2023A1785, 2023A1794, 2023A1859, 2023A1925, 2023B1675, 2023B1806, 2023B1878, and 2024A1857) on a PILATUS3 X CdTe 1M camera using synchrotron radiation (λ = 0.4132 Å). The structures were solved using SHELXT-2018[S2] and refined by a full-matrix least-squares method (LSQ) on F^2 for all reflections using SHELXL-2018[S3] and the Yadokari-XG software package.[S4] All non-hydrogen atoms were refined anisotropically, while the positions of all hydrogen atoms were calculated geometrically and refined as riding models. Supplementary crystallographic data were deposited at the Cambridge Crystallographic Data Centre (CCDC) under deposition numbers CCDC-2384224 (**1**), 2384225 (**6_K·(18-c-6)**), and 2384226 (**6_K·(18-c-6)**); these can be obtained free of charge via www.ccdc.cam.ac.uk/data_request.cif.

Table S1. Crystal data and data collection of **1**, **6_K·(18-c-6)**, and **6_K·(18-c-6)**.

Compound	**1**	**6_K·(18-c-6)**	**7_K·(18-c-6)**
Formula	$C_{54}H_{53}PSi_4$	$C_{65}H_{76}KO_6PSi_3$	$C_{46}H_{56}KO_6PSi_2$
Molecular Weight	845.29	1107.59	831.15
Temperature / K	103	103	100
λ (Å)	0.71073	0.71073	0.4132
Crystal size / mm^3	0.200×0.150×0.100	0.120×0.060×0.030	0.100×0.050×0.020
Crystal system	Monoclinic	Triclinic	Triclinic
Space group	*C*2/*c* (#15)	*P*-1 (#2)	*P*-1 (#2)
a /Å	40.2431(4)	12.27460(10)	11.2805(2)
b /Å	10.30930(10)	12.7930(2)	15.6436(3)
c /Å	22.5509(2)	20.8875(2)	26.8108(4)
α /deg	90	80.77420(10)	87.8780(10)
β / deg	95.1220(10)	79.2490(10)	83.365(2)
γ / deg	90	73.9750(10)	71.306(2)
V /Å^3	9318.52(15)	3076.30(6)	4451.62(14)
Z	8	2	4
μ / mm^{-1}	0.198	0.220	0.069
$D_{calcd.}$ / g·cm^{-3}	1.205	1.196	1.240
θ_{max}	27.500	29.148	15.773
Refl./restr./param.	10678/0/532	15529/0/989	21179/319/1180
Completeness	99.8	99.8	99.7
GOF	1.112	1.026	1.065
R_1 (I>2σ(I))	0.0633	0.0310	0.0707
wR_2 (I>2σ(I))	0.1705	0.0801	0.1752
R_1 (all data)	0.0673	0.0350	0.0802
wR_2 (all data)	0.1741	0.0826	0.1803
Largest diff. peak and hole /e·Å^{-3}	1.143, –0.681	0.410, –0.212	0.597, –0.470
CCDC DOI:	10.5517/ccdc.csd.cc2l0zg2	10.5517/ccdc.csd.cc2l0zh3	10.5517/ccdc.csd.cc2l0zj4

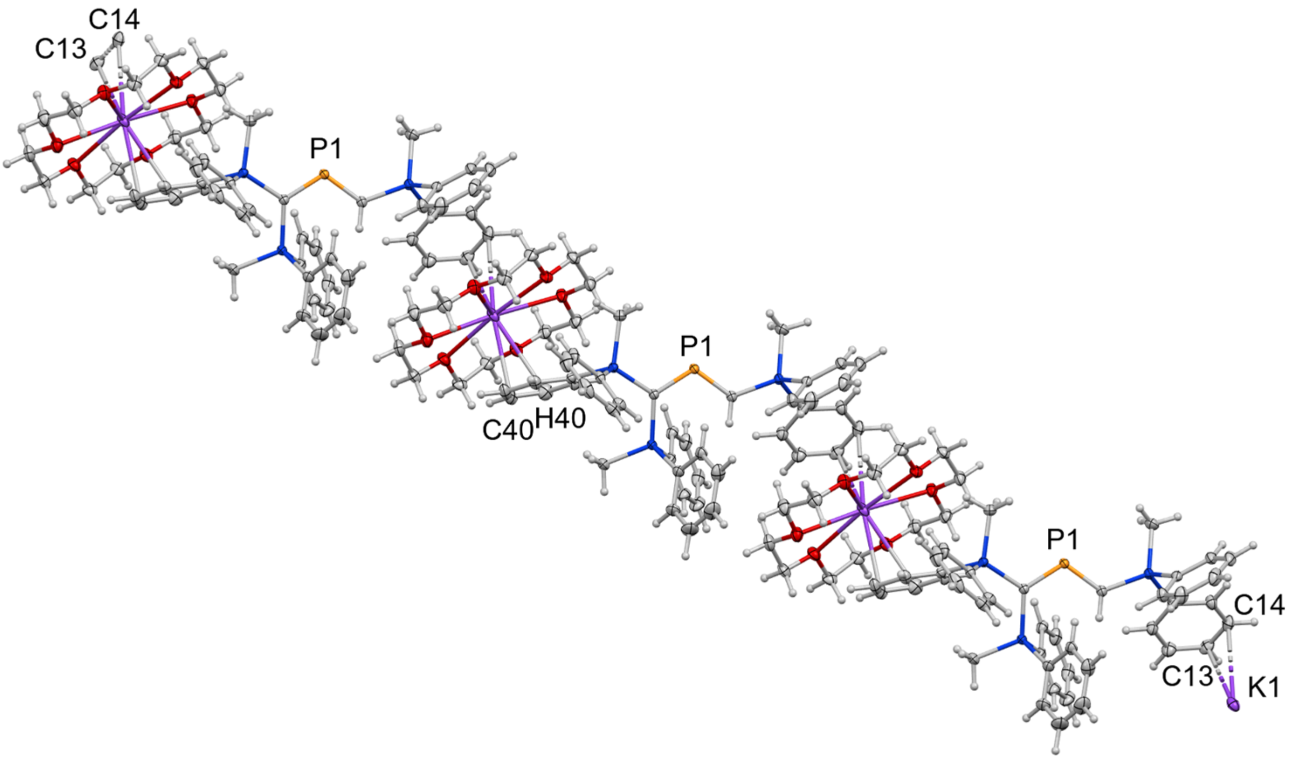

Figure S42. Packing structure of $\mathbf{6_K \cdot (18\text{-}c\text{-}6)}$.

Theoretical Calculations

Theoretical calculations for the geometry optimization and frequency calculations of **1**, **6_K·(18-c-6)**, **6**, **7**, **8**, **9**, **9'**, and model compounds (*cf.* Table S1) were carried out using the Gaussian 16 (Revision B.01 and C.01) program package.[S5] Geometry optimizations were performed at the B3PW91-D3(BJ) level using basis sets of 6-311G(3d) for P, Si, C, and H. Minimum energies for the optimized structures were confirmed by frequency calculations. GIAO calculations were performed at the GIAO-B3PW91-D3(BJ) level using basis sets of 6-311G(3d) for P, Si, C, and H. Minimum energies for the optimized structures were confirmed by frequency calculations. Energies were corrected for the zero-point energy (ZPE) term at the optimization level (E_{zero}). Computational time was generously provided by the Supercomputer Laboratory at the Institute for Chemical Research (Kyoto University). Computations were also carried out using resources of the Research Center for Computational Science, Okazaki, Japan (Projects: 24-IMS-C377/24-IMS-C397). The coordinates of the optimized structures are included in the corresponding .xyz files as supporting information.

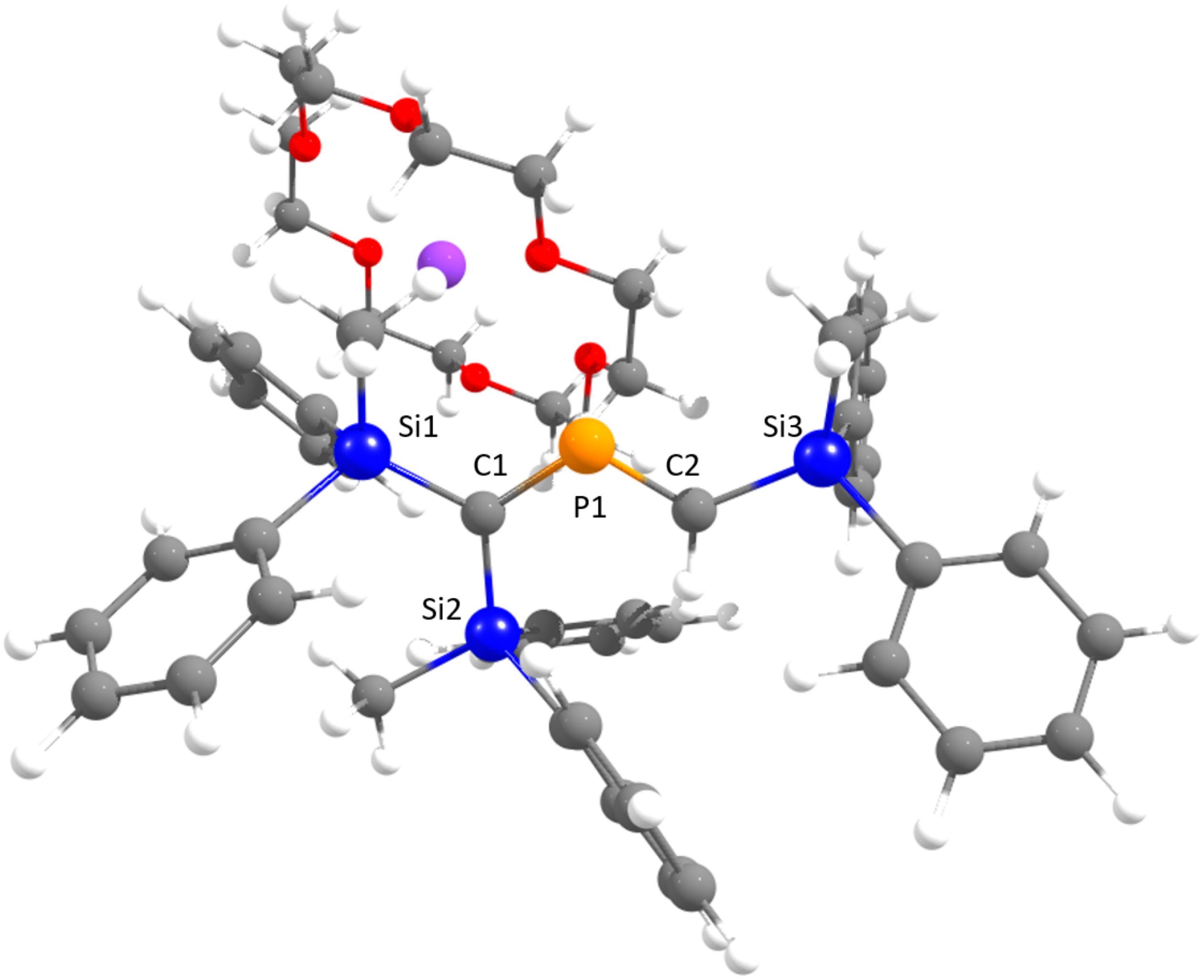

Figure S43. Optimized structure of **6_K·(18-c-6)**.

Table S2. Experimental and calculated values of the bond parameters for **6_K·(18-c-6)**.

	Experimental values (Å, deg)	Calculated values (Å, deg)
P1–C1	1.723(1)	1.724
P1–C2	1.694(1)	1.683
C1–Si1	1.8200(9)	1.810

C1–Si2	1.832(1)	1.829
C2–Si3	1.813(1)	1.812
C1–P1–C2	112.23(5)	111.8

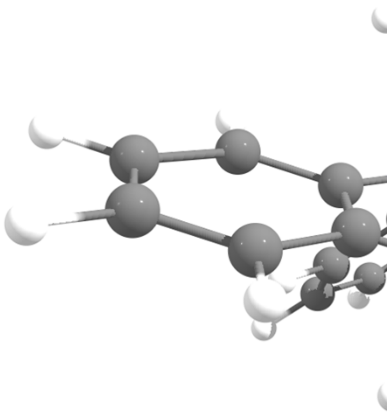

Figure S44. Optimized structure of **6.**

Table S2. Experimental and calculated values of the bond parameters for **6**.

	Experimental values (Å, deg)	Calculated values (Å, deg)
P1–C1	1.723(1)	1.726
P1–C2	1.694(1)	1.687
C1–Si1	1.8200(9)	1.805
C1–Si2	1.832(1)	1.817
C2–Si3	1.813(1)	1.805
C1–P1–C2	112.23(5)	110.3

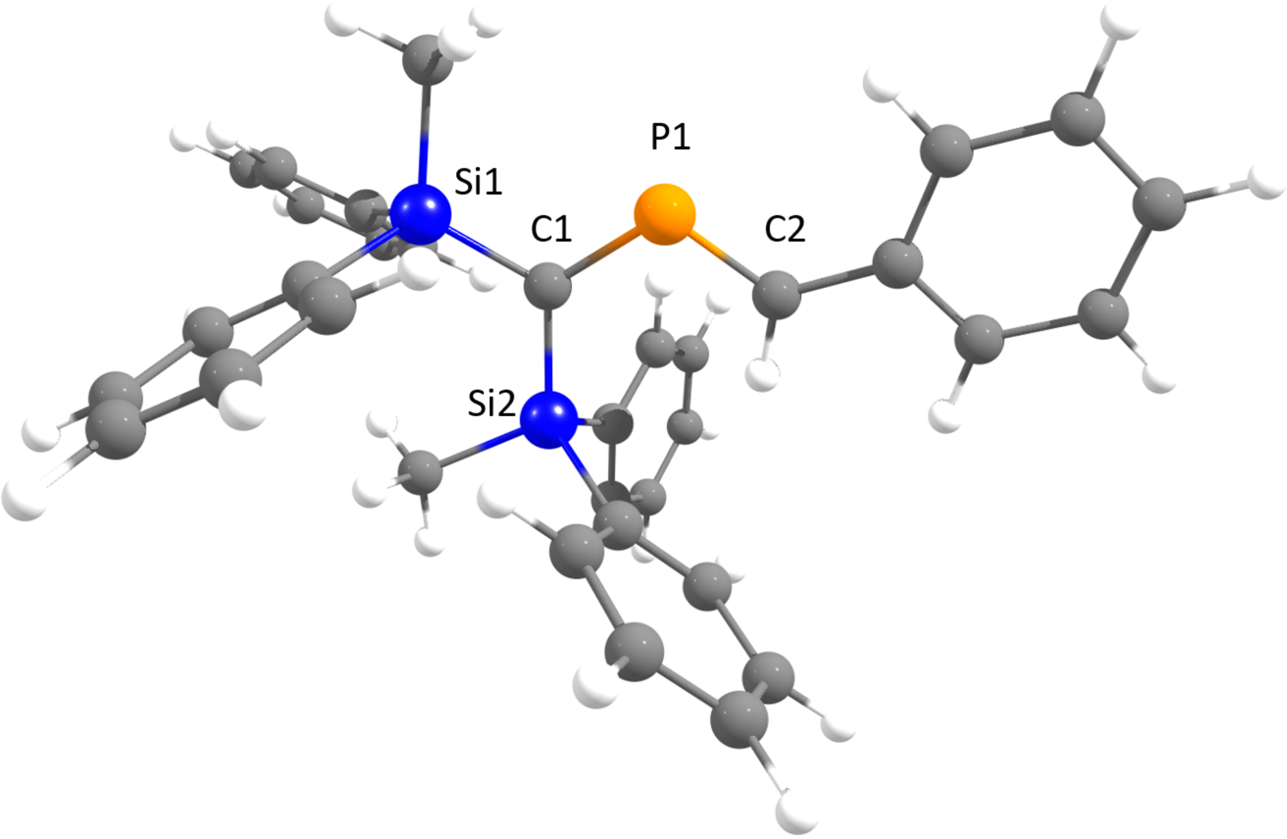

Figure S45. Optimized structure of **7**.

Table S3. Experimental and calculated values of the bond parameters for **7**.

	Experimental values (Å, deg)	Calculated values (Å, deg)
P1–C1	1.717(2)	1.729
P1–C2	1.690(3)	1.698
C1–Si1	1.818(3)	1.808
C1–Si2	1.827(3)	1.818
C1–P1–C2	112.6(1)	109.9

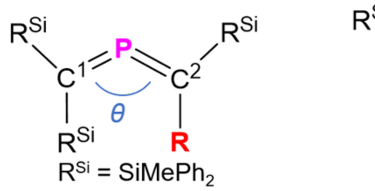

**R**	H (**6**)	Me	$SiMe_3$	$SiMe_2Ph$ (**8**)
θ (°)	110.27	115.96	123.50	114.01
φ (deg)	2.55	19.19	42.67	61.62

Figure S46. tructura 1 compar isons of bis(methylene)-λ^5-phosphane anions.

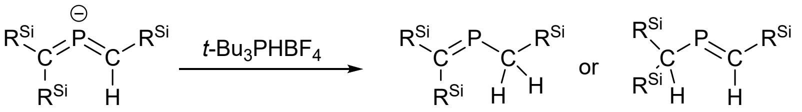

9 **9'**

Compound	9	9’
ΔE_{Zero} (a.u.)	-2796.205298	-2796.199865
ΔE_{Zero} (kcal·mol^{-1})	–3.4	0
Zero-point correction (a.u.)	0.693336	0.692410
Thermal correction to Gibbs Free Energy (a.u.)	0.610604	0.608531

Figure S47. elative energy between proton-adducts **9** and **9’**.

Natural-Resonance-Theory (NRT) calculations [S6]

To show the contributions of the considerable canonical structures of a bis(methylene)-λ^5-phosphane anion for the readers, we carried out the Natural Resonance Theory (NRT) calculations using H- and H_3Si- substituted model compounds **V** and **VI**, respectively. NRT analysis revealed two degenerate resonance structures derived from C-anionic structures for compound **V** (each contributing 46%) as shown in Figure S48. In contrast, **VI** exhibited four degenerate anion-delocalized resonance contributors, with an NRT weight of 6.4% each (totaling approximately 25%), and two degenerate C-anionic-type structures, with an NRT weight of 11.1% each (totaling 22%) (Figure S49). On the basis of the results of NRT calculations, the silyl substituents would stabilize the π-electron/negative charge delocalized electronic structure, which cause the spreading π–conjugation. On the consideration of the sum of resonance structures with the highly π-electron delocalized canonical structures, we can conclude that the obtained bis(methylene)-λ^5-phosphane anions **6** and **7** should exhibit the allene-type electronic structures with C=P=C cumulative π-bonds by delocalizing the negative charge through Si-hyperconjugation.

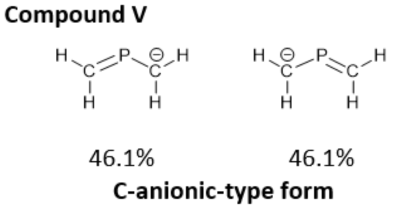

Figure S48. Resonance contributors of H-substituted model compound **V**.

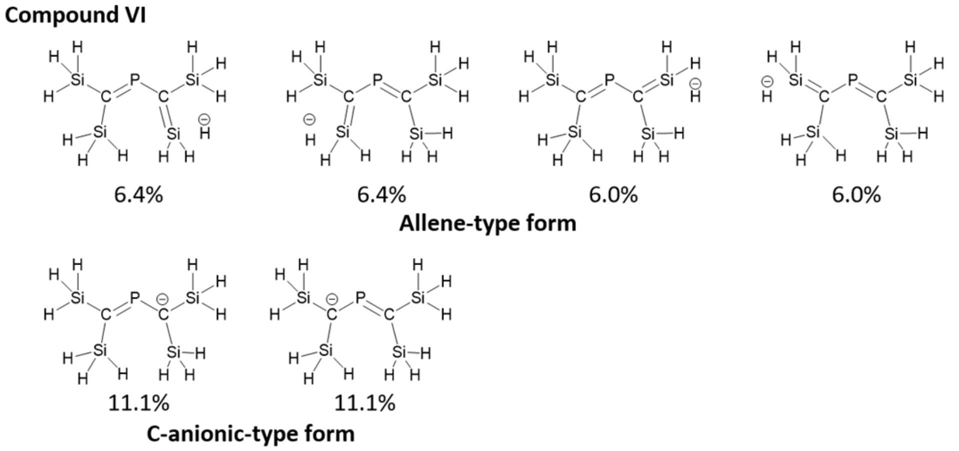

Figure S49. Resonance contributors of H_3Si-substituted model compound **VI**.

Estimation for ^{31}P NMR chemical shifts

All calculated ^{31}P shielding constants σ_{calc} were converted to ^{31}P NMR chemical shifts δ_{calc} (ppm, 85% aqueous solution of H_3PO_4) using Eq. (1),

$$\delta_{calc} = \sigma_{calc}(PMe_3) - \sigma_{calc} - 62 \text{ ppm} \qquad (1)$$

where $\sigma_{calc}(PMe_3)$ is the absolute ^{31}P NMR shielding constant of trimethylphosphine (PMe_3) calculated at the same level of theory.

Estimation of the rotation barriers for 6a and 7a

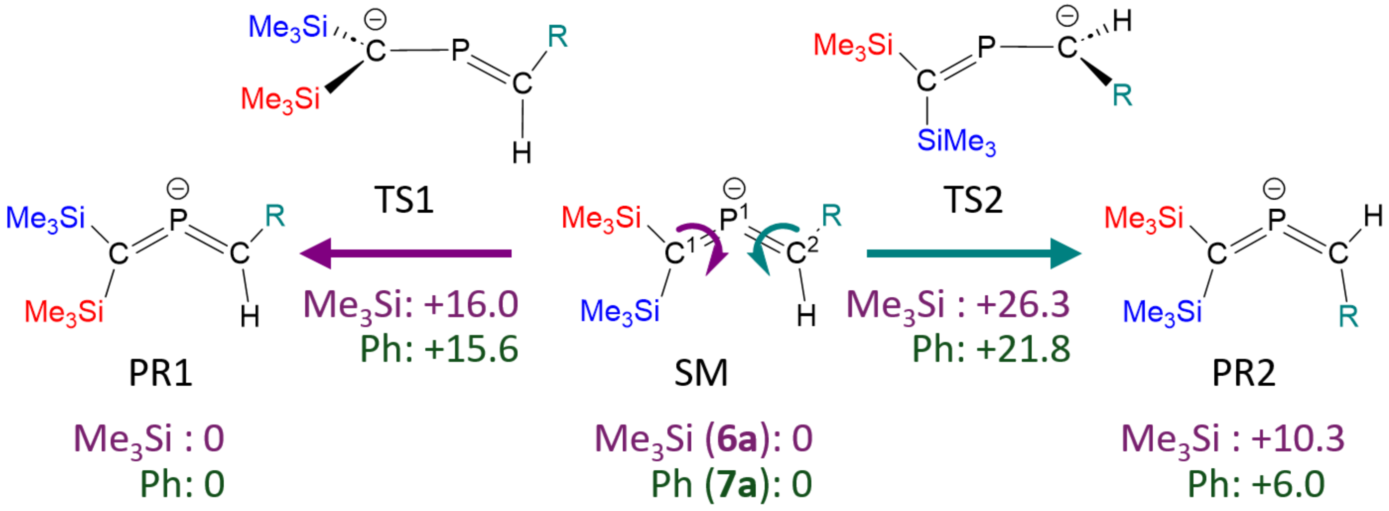

Figure S50. Activation barriers (kcal mol^{-1}) for the rotation of the C=P bond in bis(methylene)-λ^5-phosphane anions **6a** and **7a**. Geometric optimizations were carried out at the B3PW91-D3(BJ)/6-31G(d) level.

The ^{1}H NMR spectra of **6_K·(18-c-6)** in *o*-difluorobenzene at room temperature showed a highly broadened signal for the $(Ph_2MeSi)_2C$= moiety, which coalesced into one signal upon heating to 343 K (Figure S12), suggesting restricted rotation around the C1=P1 bond at room temperature similar to other bis(methylene)-λ^4-chalcogenanes. Comparable VT-NMR behavior was observed in the ^{1}H NMR spectra of **7_K·(18-c-6)** in C_6D_6 (Figure S18). Theoretical calculations for model compounds **6a** and **7a**, which bear Me_3Si groups instead of Ph_2MeSi groups, suggested rotation barriers of 16.0 kcal·mol^{-1} and 15.6 kcal·mol^{-1} for their C1=P1 bonds, and 26.3 kcal·mol^{-1} and 21.8 kcal·mol^{-1} for their P1=C2 bonds, respectively. These results suggest that the C1=P1 bond can rotate slowly, even at room temperature (Figure S50). The smaller rotation barriers of the P1=C1 bonds in **6a** and **7a** relative to those of P1=C2 also support the unsymmetric contribution of the resonance structures **6_A** and **7_A** (Fig. 4). These rotation barriers are almost similar to those of the C=S bonds in previously reported bis(methylene)-λ^4-sulfanes.

Theoretical calculations were carried out at the B3PW91-D3(bj)/6-31G(d) level for model compounds **6a** and **7a**, which contain Me_3Si groups instead of Ph_2MeSi groups. All calculations included the solvent effect with the scrf method. The data for *o*-difluorobenzene for the calculations are shown in table S4. To locate the transition-state structures, the synchronous transit-guided quasi-Newton (STQN) method was employed in Gaussian using the QST3 keywords. After identifying the predicted transition-state structures, these structures were further optimized using the eigenvector-following method with the Opt=TS keywords. Finally, an internal-reaction-coordinate (IRC) calculation was performed. Activation barriers were estimated as the energy differences between transition states (TS) and the grand state of products (PR) and

starting materials (SM). These calculations provided the energy diagram for the rotation barriers of **6a** and **7a** as shown in Figure S48.

Table S4

***o*-difluorobenzene**	Eps	Epsinf	R_{Solv}	Density
MW: 114.09, D = 1.16 g/mL	13.8	2.082	3.99	0.006125

Eps=x: Specifies the static (or zero-frequency) dielectric constant of the solvent.

EpsInf=x: Specifies the dynamic (or optical) dielectric constant of the solvent.

R_{Solv}=x: Specifies the solvent radius (in Angstroms).

Density=x: Density: 1.16 g/mL Indeed, if ρ, D, and MW to be density in g/mL, density in $particle$/Å^3, and molecular weight in gr/mol of the desirable solvent, then: $D = \rho/(1.66 \times MW)$.

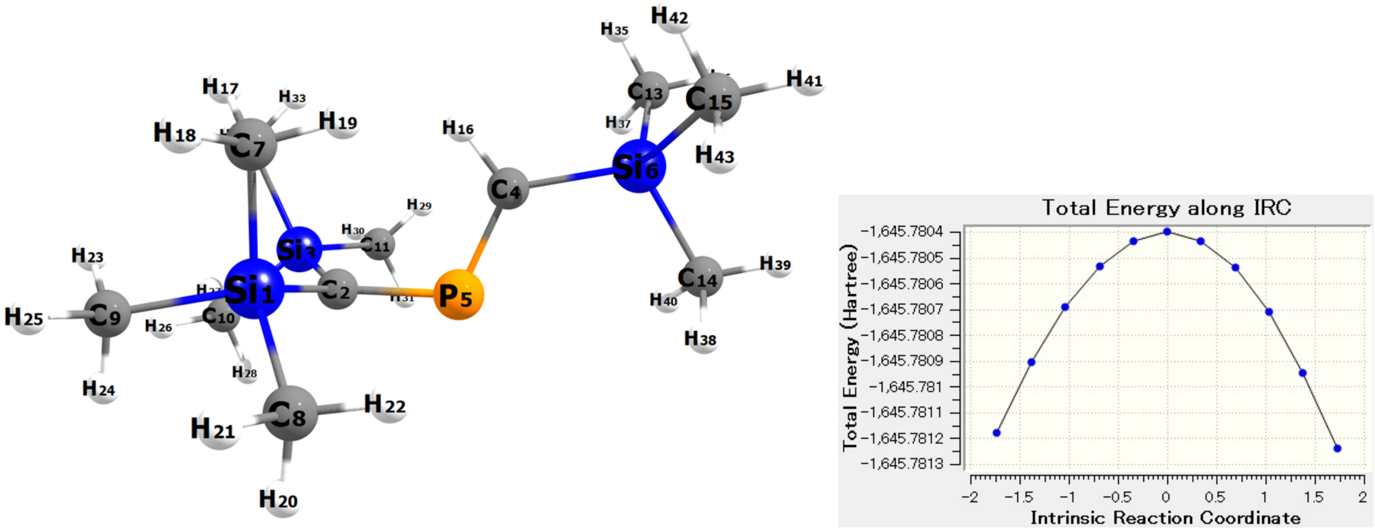

Figure S51. Structure of a transition state between **6a** and **Pr1** with a negative frequency of -122.2623 and its IRC path.

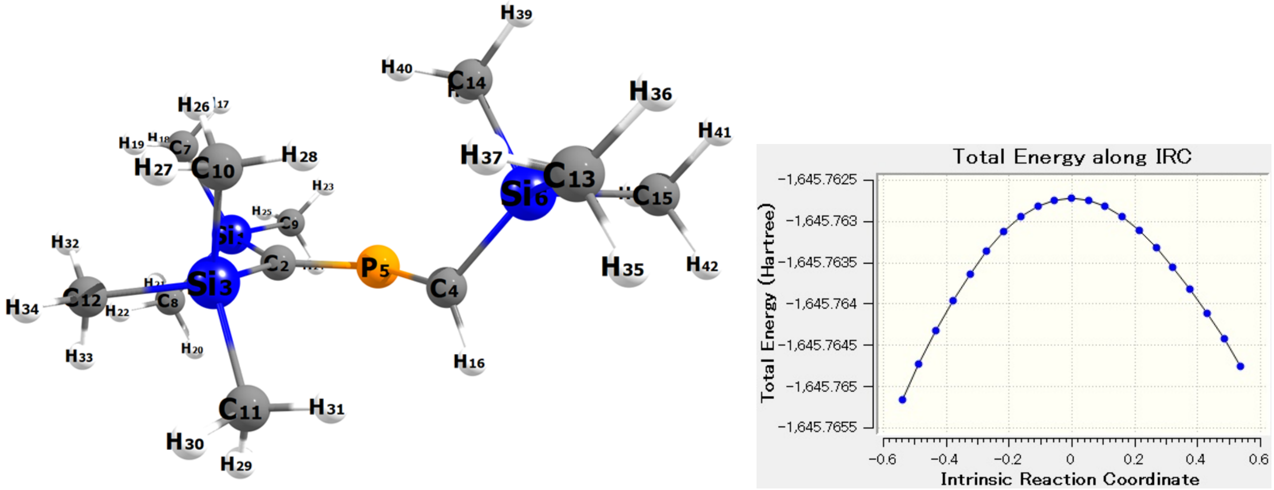

Figure S52. Structure of a transition state between **6a** and **Pr2** with a negative frequency of -688.7312 and its IRC path.

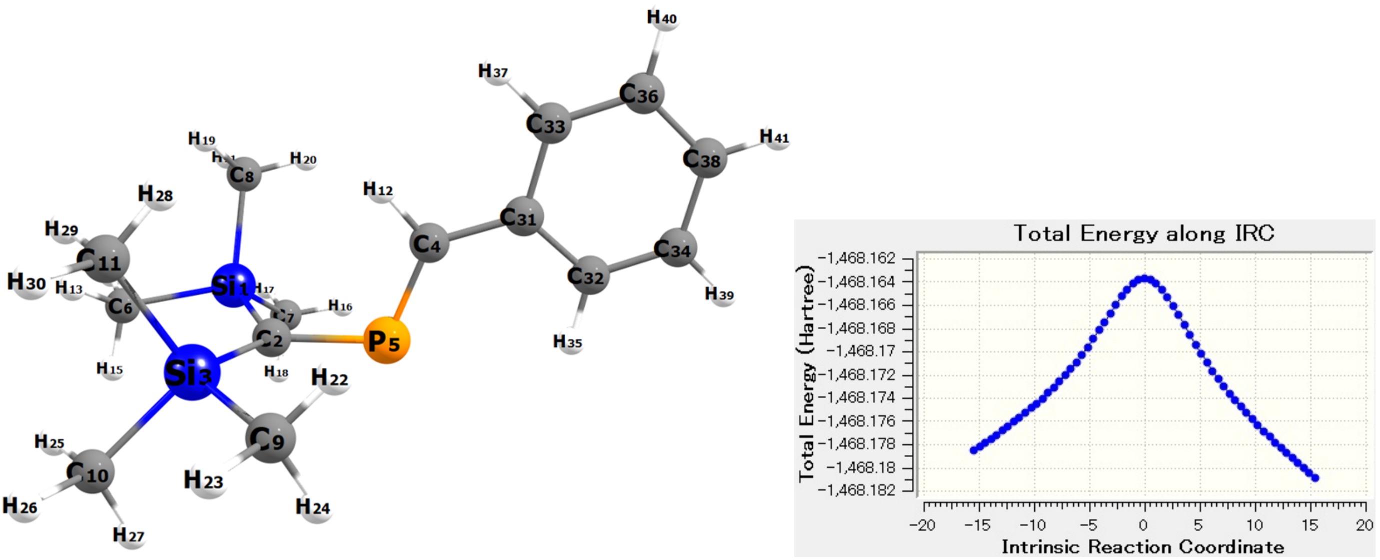

Figure S53. Structure of a transition state between **7a** and **Pr1** with a negative frequency of -141.4047 and its IRC path.

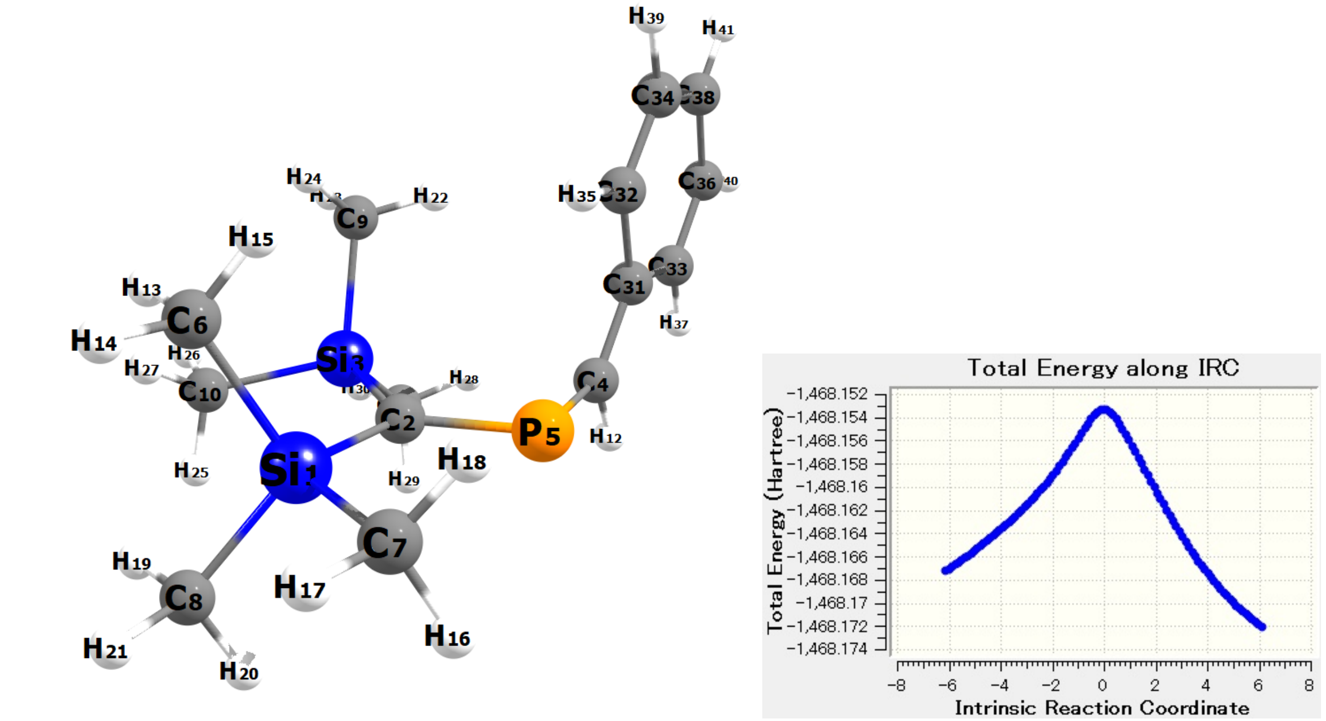

Figure S54. Structure of a transition state between **7a** and **Pr2** with a negative frequency of -473.7128 and its IRC path.

·Comparison of ΔE_{ZERO} between the desilylation and deprotonation reaction of 1.

To investigate the reaction mechanism for desilylation reaction, we performed theoretical calculations on the potential energy surface of both a deprotonation reaction (upper) and a desilylation reaction of the phosphaalekene (**SM**) as showns in Figure S55. The reaction barrier for the deprotonation product via **TS1** is smaller than that of desilylation product via **TS2**, while the product of desilylation reaction (**Pr2**) is significantly stable compared to that of deprotonation reaction (**Pr1**). As the result, it was found that the formation of bis(methylene)-λ^5-phosphane anion **6** is thermodynamically favored reactions.

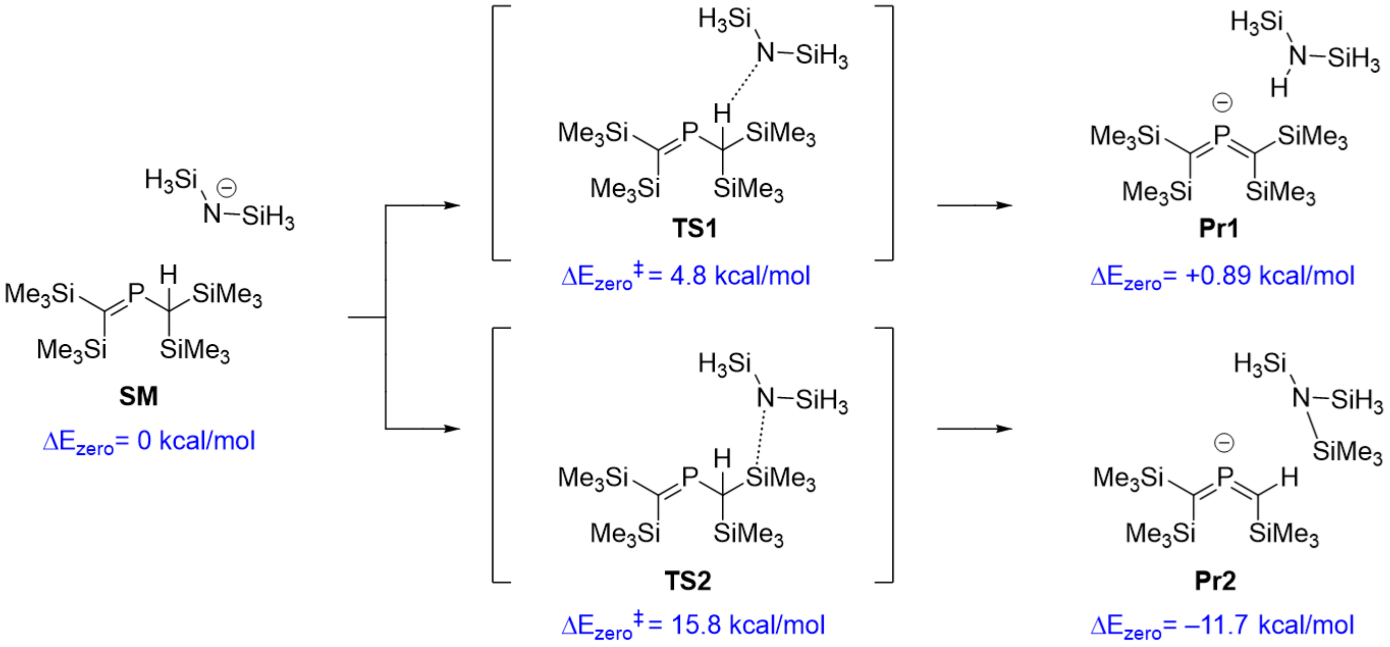

Figure S55. DFT-derived energy diagram for the desilylation and the deprotonation pathways, calculated at the B3PW91-D3(BJ)/6-31G(d) level.

·Atom-in-molecules analyses of bis(methylene)-λ^5-phosphane anions and bis(methylene)-λ^4-sulfanes.

Topological analysis of the electron density was performed in MultiWFN (version 3.7).[S7] Bond paths and bond critical point were visualized in VMD.[S8]

As a result of the atoms-in-molecule (AIM) analysis of compound 6, the C—P bonds are characterized by a positive electron density (ρ_{BCP}): 0.136 eÅ^{-3} for C2—P1 and 0.142 eÅ^{-3} for C4—P1, as well as a small positive Laplacian ($\nabla^2\rho_{BCP}$): 0.774 eÅ^{-5} for C2—P1 and 0.629 eÅ^{-5} for C4—P1 at the bond critical points (BCPs). The bond ellipticity at the BCP of the C—P bonds was 0.070 for C2—P1 and 0.021 for P1—C4. For comparison, the corresponding parameter for the C—C bond in a benzene ring ranges from 0.23 to 0.28, while for a C—Si single bond, it ranges from 0.0063 to 0.013. The AIM analysis of compound **7** reveals similar characteristics: the C—P bonds display a positive electron density (ρ_{BCP}): 0.139 e$^-$Å^{-3} for C3—P1 and 0.136 e$^-$Å^{-3} for P1—C2, along with a small positive Laplacian ($\nabla^2\rho_{BCP}$): 0.734 e$^-$Å^{-5} for C2—P1 and 0.619 eÅ^{-5} for C4—P1 at the BCPs. The bond ellipticity at the BCP of the C—P bonds was 0.040 for C2—P1 and 0.037 for P1—C4. In contrast, the bond ellipticities at the BCPs of C—S bonds (ranging from 0.18 to 0.24) in bis(methylene)-λ^4-sulfanes **VII** and **VIII** indicate significant π-bonding character (Figures S56–S59). Furthermore, we performed an AIM analysis of phosphaalkene **1**. The bond ellipticities of **1** were 0.199 for the C–P single bond and 0.0397 for the C=P double bond (Figure S60).

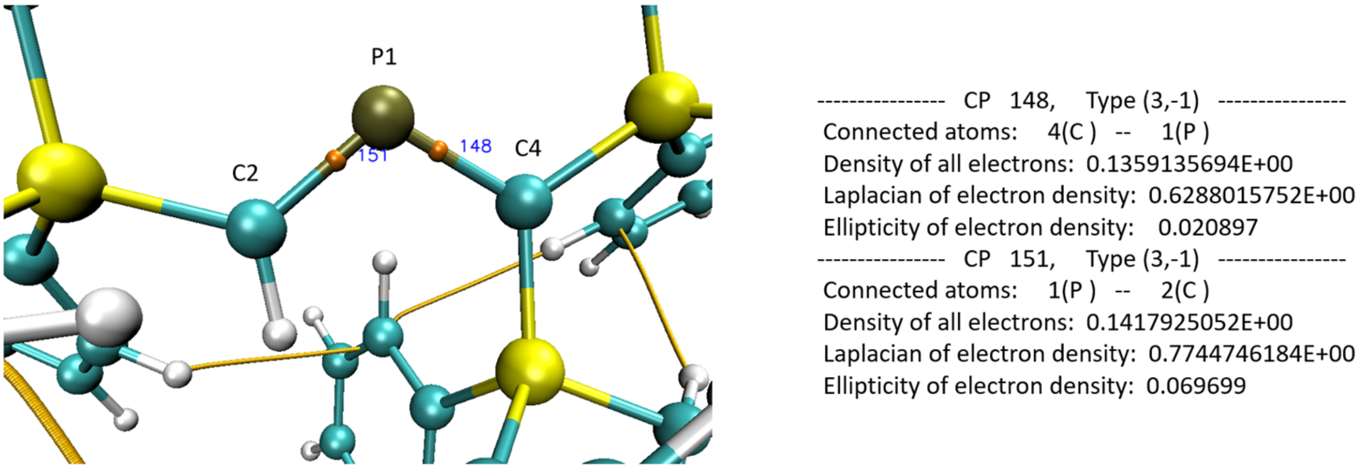

Figure S56. Bond critical points of C-P-C moiety and bond paths in **6**, calculated at the B3PW91-D3(BJ)/6-311G(d) level.

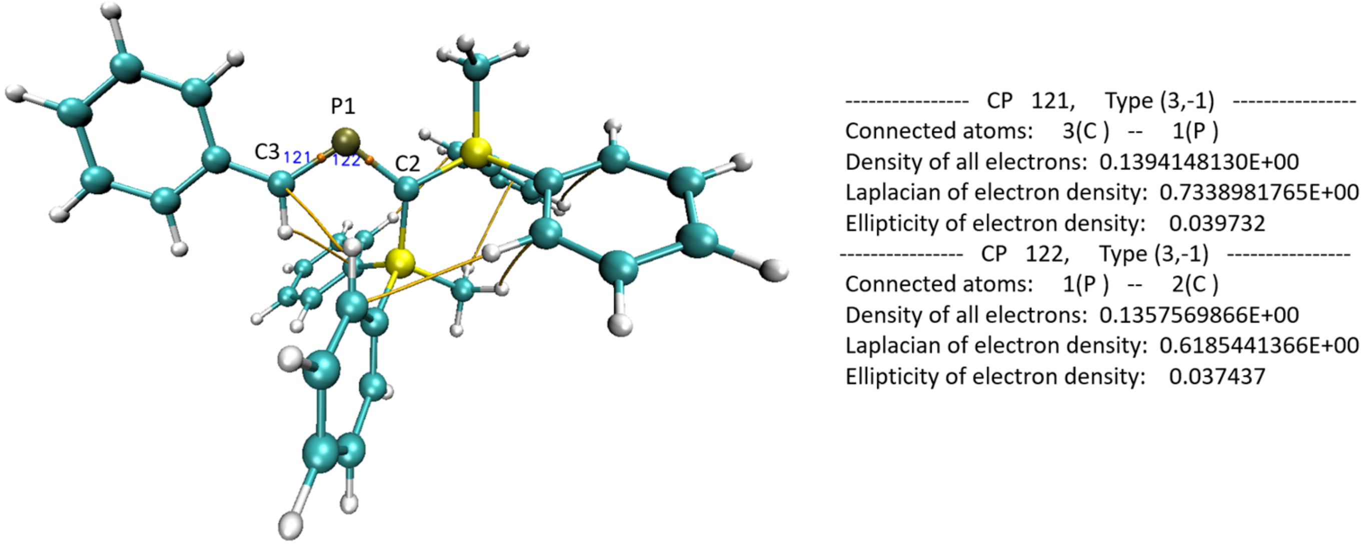

Figure S57. Bond critical points of C-P-C moiety and bond paths in **7**, calculated at the B3PW91-D3(BJ)/6-311G(d) level.

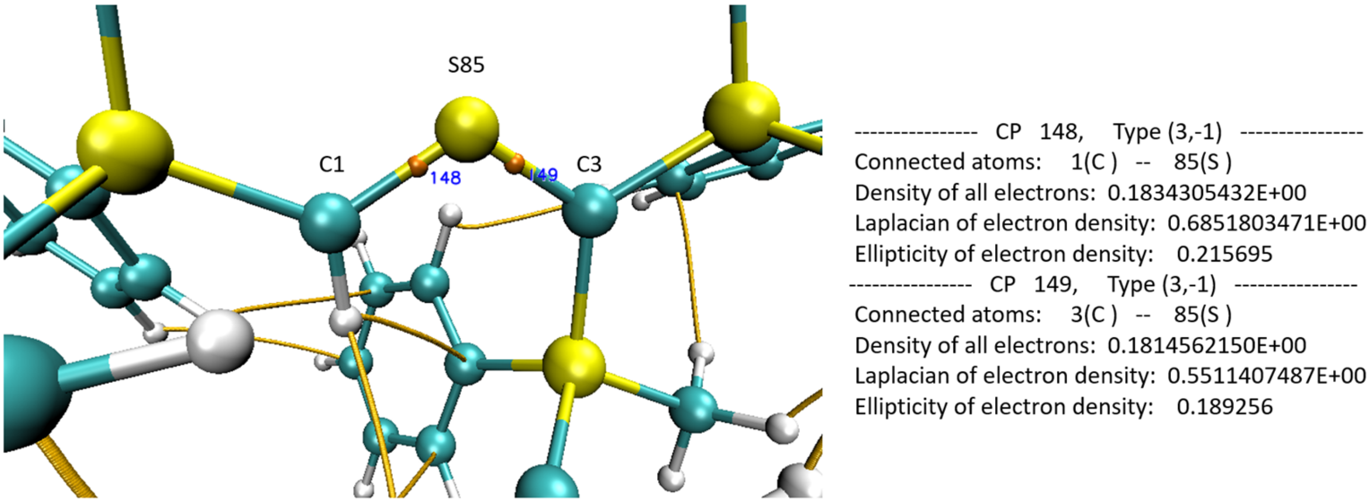

Figure S58. Bond critical points and bond paths of C-S-C moiety in bis(methylene)-λ-sulfane**VII**, calculated at the

B3PW91-D3(BJ)/6-311G(d) level.

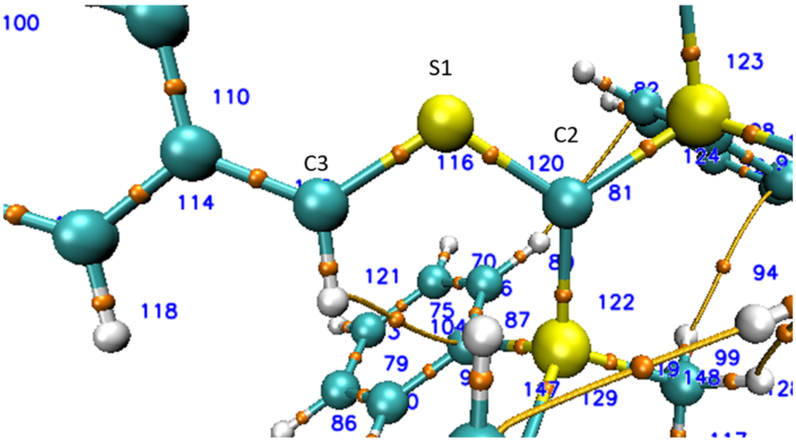

---------------- CP 116, Type (3,-1) ----------------
Connected atoms: 1(S) -- 3(C)
Density of all electrons: 0.1806611520E+00
Laplacian of electron density: 0.6438772010E+00
Ellipticity of electron density: 0.180875
---------------- CP 120, Type (3,-1) ----------------
Connected atoms: 1(S) -- 2(C)
Density of all electrons: 0.1811393839E+00
Laplacian of electron density: 0.5378007380E+00
Ellipticity of electron density: 0.237114

Figure S59. Bond critical points and bond paths of C-S-C moiety in bis(methylene)-λ-sulfane **VIII**, calculated at the B3PW91-D3(BJ)/6-311G(d) level.

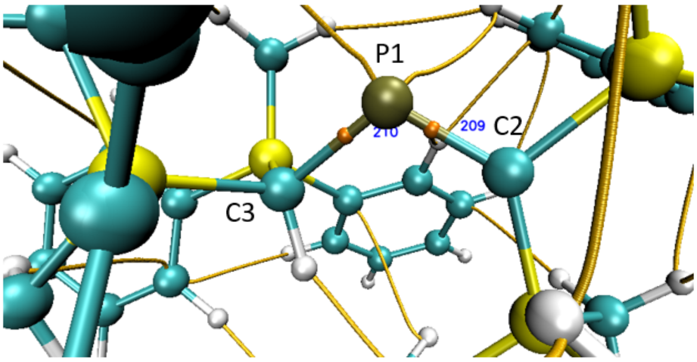

---------------- CP 209, Type (3,-1) ----------------
Connected atoms: 1(P) -- 2(C)
Density of all electrons: 0.1468366327E+00
Laplacian of electron density: 0.8286015272E+00
Ellipticity of electron density: 0.099304
---------------- CP 210, Type (3,-1) ----------------
Connected atoms: 1(P) -- 3(C)
Density of all electrons: 0.1225423953E+00
Laplacian of electron density: 0.3255458106E+00
Ellipticity of electron density: 0.198677

Figure S60. Bond critical points and bond paths of C-P-C moiety in phosphaalkene **1**, calculated at the B3PW91-D3(BJ)/6-311G(d) level.

References

S1) A. Inoue, J. Kondo, H. Shinokubo, K. Oshima, *Chem. Lett.* **2001**, *30*, 956–957.

S2) G. M. Sheldrick, *Acta Cryst.,* **2015**, *A71*, 3-8.

S3) G. M. Sheldrick, *Acta Cryst.,* **2015**, *C71*, 3-8.

S4) Wakita, K. (2001). Yadokari-XG. Software for Crystal Structure Analyses. Release of Software (Yadokari-XG 2009) for Crystal Structure Analyses, Kabuto, C., Akine, S., Nemoto, T. and Kwon, E. *J. Cryst. Soc. Jpn.*, **2009,** *51*, 218-224.

S5) Gaussian 16, Revision B.01 and C.01, M. J. Frisch, G. W. Trucks, H. B. Schlegel, G. E. Scuseria, M. A. Robb, J. R. Cheeseman, G. Scalmani, V. Barone, G. A. Petersson, H. Nakatsuji, X. Li, M. Caricato, A. V. Marenich, J. Bloino, B. G. Janesko, R. Gomperts, B. Mennucci, H. P. Hratchian, J. V. Ortiz, A. F. Izmaylov, J. L. Sonnenberg, D. Williams-Young, F. Ding, F. Lipparini, F. Egidi, J. Goings, B. Peng, A. Petrone, T. Henderson, D. Ranasinghe, V. G. Zakrzewski, J. Gao, N. Rega, G. Zheng, W. Liang, M. Hada, M. Ehara, K. Toyota, R. Fukuda, J. Hasegawa, M. Ishida, T. Nakajima, Y. Honda, O. Kitao, H. Nakai, T. Vreven, K. Throssell, J. A. Montgomery, Jr., J. E. Peralta, F. Ogliaro, M. J. Bearpark, J. J. Heyd, E. N. Brothers, K. N. Kudin, V. N. Staroverov, T. A. Keith, R. Kobayashi, J. Normand, K. Raghavachari, A. P. Rendell, J. C. Burant, S. S. Iyengar, J. Tomasi, M. Cossi, J. M. Millam, M. Klene, C. Adamo, R. Cammi, J. W. Ochterski, R. L. Martin, K. Morokuma, O. Farkas, J. B. Foresman, D. J. Fox, Gaussian, Inc., Wallingford CT, **2016**.

S6) NBO7 program: E. D. Glendening, C.R. Landis and G. Weinhold, *J. Comput. Chem.* 2019, **40**, 2234-2241.

S7) MultiWFM program: T. Lu, *J. Chem. Phys.*, 2024, **161**, 082503.

S8) VMD: program: W. Humphrey, A. Dalke and K. Schulten, *J. Mol. Graph.*, 1996, **14**, 33–8, 27–8.
